# Datasets on the statistical and algebraic properties of primitive Pythagorean triples

**DOI:** 10.1016/j.dib.2017.08.021

**Published:** 2017-09-01

**Authors:** Hilary I. Okagbue, Muminu O. Adamu, Pelumi E. Oguntunde, Abiodun A. Opanuga, Enahoro A. Owoloko, Sheila A. Bishop

**Affiliations:** a Department of Mathematics, Covenant University, Canaanland, Ota, Nigeria; b Department of Mathematics, University of Lagos, Akoka, Lagos, Nigeria

**Keywords:** Pythagorean triples, Primitive Pythagorean triples, Correlation, Normality test, Skewness, Statistics

## Abstract

The data in this article was obtained from the algebraic and statistical analysis of the first 331 primitive Pythagorean triples. The ordered sample is a subset of the larger Pythagorean triples. A primitive Pythagorean triple consists of three integers a, b and c such that; $a^{2}+b^{2}=c^{2}$. A primitive Pythagorean triple is one which the greatest common divisor (gcd), that is; gcd(a,b,c)=1 or a, b and c are coprime, and pairwise coprime. The dataset describe the various algebraic and statistical manipulations of the integers a, b and c that constitute the primitive Pythagorean triples. The correlation between the integers at each analysis was included. The data analysis of the non-normal nature of the integers was also included in this article. The data is open to criticism, adaptation and detailed extended analysis.

**Specifications Table**

**Table:** 

Subject area	*Mathematics*
More specific subject area	*Number Statistics*
Type of data	*Tables and Figures*
How data was acquired	*The raw data is available in mathematical literature*.
Data format	*Analyzed*
Experimental factors	*Negative and non-primitive Pythagorean triples and negative were not considered*.
Experimental features	*Correlation coefficient, Normality tests*.
Data source location	*Covenant University Mathematics Laboratory, Ota, Nigeria*
Data accessibility	*All the data are in this data article*

**Value of the data**

• The data provides the descriptive statistics of the primitive Pythagorean triples
• The data when completely analyzed can provide insight on the various patterns that characterizes the primitive Pythagorean triples.
• The data analysis can be applied to other known numbers. That is the study of probability distribution of numbers.
• The data can provide more clues on the normal or non-normal nature of similar numbers.

## 1 Data

The data in this article is a description of some observed algebraic and statistical properties of the integers that constitute the primitive Pythagorean triples. Correlation between the pairs of the integers was investigated and different nature and strength of relationships were obtained. The line plots were used to visualize the patterns of distribution of variability of the integers.

The detailed description and the contents of the data are contained in different sub sections.

### 1.1 The descriptive statistics of the integers a, b and c

The description statistics and the differences between the ordered pairs of the integers that make up the primitive Pythagorean triples can be assessed as Supplementary Data 1.

Scatter plots of the three positive integers and the differences between each pair that constitute the primitive Pythagorean triples and the mean plots are shown in Supplementary Data 2. The mean is monotone increasing.

Variance is the measure of variability or deviation from the mean or median. The line plots of the variance and skewness of the primitive Pythagorean triples are shown in Supplementary Data 3. The variance is increasing as the ordered sample size increases.

Different types of correlation coefficients for the integers a, b and c of the primitive Pythagorean triples were obtained and shown in Table 1. There are strong positive correlations between b and c and moderate positive correlation between a and b, and a and c.

**Table 1:** Correlation coefficients of a, b and c.

	**Correlation coefficient**	**b**	**c**
a	Pearson correlation	0.535	0.682
	Kendall's tau	0.427	0.535
	Spearman's rho	0.583	0.699
			
b	Pearson correlation		0.981
	Kendall's tau		0.893
	Spearman's rho		0.983

Different types of correlation coefficients for the integers (b–a, c–b and c–a) of the primitive Pythagorean triples were obtained and shown in Table 2. Increase or decrease in (b–a) leads to decrease or increase in (c–b). However, (c–a) and (b–a) are strongly positively correlated.

**Table 2:** Correlation coefficients of b–a, c–b and c–a.

	**Correlation coefficient**	**c-b**	**c-a**
b–a	Pearson correlation	−0.297	0.965
	Kendall's tau	−0.150	0.826
	Spearman's rho	−0.201	0.940
c–b	Pearson correlation		−0.037
	Kendall's tau		0.042
	Spearman's rho		0.057

### 1.2 The trigonometric integers of the primitive Pythagorean triples

The trigonometric aspects of the integers a, b and c that constitute the primitive Pythagorean triples were considered. The details are shown in Supplementary Data 4.

The summary of scatter plots of the sine, cosine and tangent of a, b and c are shown in Supplementary Data 5.

Different types of correlation coefficients for the trigonometric values of integers a, b and c of the primitive Pythagorean triples were obtained and shown in Table 3, Table 4, Table 5. Weak correlations were the results.

**Table 3:** Correlation coefficients of sine a, sine b and sine c.

	**Correlation coefficient**	**sine b**	**sine c**
sine a	Pearson correlation	0.033	−0.021
	Kendall's tau	0.022	−0.025
	Spearman's rho	0.032	−0.038
			
sine b	Pearson correlation		0.400
	Kendall's tau		0.265
	Spearman's rho		0.378

**Table 4:** Correlation coefficients of cosine a, cosine b and cosine c.

	**Correlation coefficient**	**cosine b**	**cosine c**
cosine a	Pearson correlation	0.005	−0.036
	Kendall's tau	0.008	−0.016
	Spearman's rho	0.009	−0.025
			
cosine b	Pearson correlation		0.341
	Kendall's tau		0.240
	Spearman's rho		0.333

**Table 5:** Correlation coefficients of tangent a, tangent b and tangent c.

	**Correlation coefficient**	**tangent b**	**tangent c**
tangent a	Pearson correlation	−0.016	−0.064
	Kendall's tau	−0.039	0.000
	Spearman's rho	−0.059	0.007
			
tangent b	Pearson correlation		0.011
	Kendall's tau		0.212
	Spearman's rho		0.282

### 1.3 The hyperbolic transformations of integers of the primitive Pythagorean triples

The hyperbolic aspects of the integers a, b and c that constitute the Primitive Pythagorean triples were considered. The details are shown in Supplementary Data 6.

The summary of scatter plots of the sinh, cosh and tanh of a, b and c are shown in Supplementary Data 7.

Different types of correlation coefficient for the hyperbolic values of integers a, b and c of the primitive Pythagorean triples were obtained and shown in Table 6, Table 7, Table 8. The correlations are weak with the exception of hyperbolic of b and c.

**Table 6:** Correlation coefficients of sinh a, sinh b and sinh c.

	**Correlation coefficient**	**sinh b**	**sinh c**
sin h a	Pearson correlation	−0.015	0.323
	Kendall's tau	0.427	0.535
	Spearman's rho	0.583	0.699
			
sin h b	Pearson correlation		0.468
	Kendall's tau		0.893
	Spearman's rho		0.983

**Table 7:** Correlation coefficients of cosh a, cosh b and cosh c.

	**Correlation coefficient**	**cosh b**	**cosh c**
cos h a	Pearson correlation	−0.015	0.323
	Kendall's tau	0.427	0.535
	Spearman's rho	0.583	0.699
			
cos h b	Pearson correlation		0.468
	Kendall's tau		0.893
	Spearman's rho		0.983

**Table 8:** Correlation coefficients of tan h a, tan h b and tan h c.

	**Correlation coefficient**	**tan h b**	**tan h c**
tan h a	Pearson correlation	0.640	0.638
	Kendall's tau	0.505	0.536
	Spearman's rho	0.615	0.645
			
tan h b	Pearson correlation		0.995
	Kendall's tau		0.935
	Spearman's rho		0.962

### 1.4 The logarithmic and exponential transformations of integers of the primitive Pythagorean triples

The logarithmic and exponential aspects of the integers a, b and c that constitute the Primitive Pythagorean triples were considered. The details are shown in Supplementary Data 8.

The summary of scatter plots of the log, natural log and exponential of the inverse of a, b and c are shown in Supplementary Data 9.

Different types of correlation coefficient for the logarithmic, natural log and exponential values of integers a, b and c of the primitive Pythagorean triples were obtained and shown in Tables 9–11. Strong positive correlations are the results.

**Table 9:** Correlation coefficients of log a, log b and log c.

	**Correlation coefficient**	**log b**	**log c**
log a	Pearson correlation	0.708	0.766
	Kendall's tau	0.427	0.535
	Spearman's rho	0.583	0.699
			
log b	Pearson correlation		0.995
	Kendall's tau		0.893
	Spearman's rho		0.983

**Table 10:** Correlation coefficients of ln a, ln b and ln c.

	**Correlation coefficient**	**ln b**	**ln c**
ln a	Pearson correlation	0.708	0.766
	Kendall's tau	0.427	0.535
	Spearman's rho	0.583	0.699
			
ln b	Pearson correlation		0.995
	Kendall's tau		0.893
	Spearman's rho		0.983

**Table 11:** Correlation coefficients of exp 1/a, exp 1/b and exp 1/c.

	**Correlation coefficient**	**exp 1/b**	**exp 1/c**
exp 1/a	Pearson correlation	0.893	0.920
	Kendall's tau	0.427	0.535
	Spearman's rho	0.583	0.699
			
exp 1/b	Pearson correlation		0.998
	Kendall's tau		0.893
	Spearman's rho		0.983

### 1.5 The digital sum and digital root (iterative digits sum) of the integers of the primitive Pythagorean triples

The digital sum and iterative digits sum of the integers that constitute the primitive Pythagorean triples were considered. The details are shown in Supplementary Data 10.

The summary of scatter plots of the digital sum and iterative digits sum of a, b and c is shown in Supplementary Data 11.

Different types of correlation coefficient for the digital sum and iterative digits sum values of integers a, b and c of the primitive Pythagorean triples were obtained and shown in Table 12, Table 13. Weak correlations are the main results here.

**Table 12:** Correlation coefficients of digital sum of a, b and c.

	**Correlation coefficient**	**Digits sum b**	**Digits sum c**
Digits sum a	Pearson correlation	0.147	0.139
	Kendall's tau	0.120	0.098
	Spearman's rho	0.165	0.139
			
Digits sum b	Pearson correlation		0.283
	Kendall's tau		0.225
	Spearman's rho		0.294

**Table 13:** Correlation coefficients of Iterative digits sum of a, b and c.

	**Correlation coefficient**	**Iterative digits sum b**	**Iterative digits sum c**
Iterative digits sum a	Pearson correlation	−0.081	0.007
	Kendall's tau	−0.062	0.008
	Spearman's rho	−0.083	0.010
			
Iterative digits sum b	Pearson correlation		0.028
	Kendall's tau		0.024
	Spearman's rho		0.026

### 1.6 Test of normality for a, b and c

Normality tests are conducted to show how well the given data is fitted by normal distribution and the likelihood of the random variables that defined the given data is normally distributed. The data was subjected to some frequentist tests and the results are shown in Table 14, Table 15, Table 16. The null hypothesis implies normality while the alternative implies otherwise.

**Table 14:** Test of normality for a.

Test	Details	Decision
Kolmogorov-Smirnov test	Statistic=0.123,pvalue=0.000	Accept alternative hypothesis
Shapiro-Wilk test	Statistic=0.902,pvalue=0.000	Accept alternative hypothesis
Jarque-Bera Normality test	$JB=45.216>4.605=\chi_{0.01,2}^{2}$	Accept alternative hypothesis
D’Agostino Skewness test	skew=0.90526,Z=5.96690	Accept alternative hypothesis, data have a skewness
	p−value=0.0000	
Geary Kurtosis test	0.8258283≠0.7979	Accept alternative hypothesis
Anscombe-Glynn kurtosis test	kurtosis=2.97770,Z=0.10578	Accept alternative hypothesis, kurtosis is not equal to 3
	p−value=0.9158	
Anderson-Darling test	p−value<0.001	Accept alternative hypothesis
Lilliefors-van Soest test	p−value<0.01	Accept alternative hypothesis
Cramer-von Mises test	p−value<0.005	Accept alternative hypothesis
Ryan-Joiner test	p−value<0.010	Accept alternative hypothesis

**Table 15:** Test of normality for b.

Test	Details	Decision
Kolmogorov-Smirnov test	Statistic=0.065,pvalue=0.002	Accept alternative hypothesis
Shapiro-Wilk test	Statistic=0.963,pvalue=0.000	Accept alternative hypothesis
Jarque-Bera Normality test	$JB=17.231>4.605=\chi_{0.01,2}^{2}$	Accept alternative hypothesis
D’Agostino Skewness test	skew=0.077656,Z=0.588370	Accept alternative hypothesis, data have a skewness
	p−value=0.5563	
Geary Kurtosis test	0.8588865≠0.7979	Accept alternative hypothesis
Anscombe-Glynn kurtosis test	kurtosis=1.8931,Z=−10.3490	Accept alternative hypothesis, kurtosis is not equal to 3
	p−value=0.0000	
Anderson-Darling test	p−value<0.001	Accept alternative hypothesis
Lilliefors-van Soest test	p−value<0.01	Accept alternative hypothesis
Cramer-von Mises test	p−value<0.005	Accept alternative hypothesis
Ryan-Joiner test	p−value<0.010	Accept alternative hypothesis

**Table 16:** Test of normality for c.

Test	Details	Decision
Kolmogorov-Smirnov test	Statistic=0.065,pvalue=0.002	Accept alternative hypothesis
Shapiro-Wilk test	Statistic=0.955,pvalue=0.000	Accept alternative hypothesis
Jarque-Bera Normality test	$JB=19.681>4.605=\chi_{0.01,2}^{2}$	Accept alternative hypothesis
D’Agostino Skewness test	skew=0.0012575,Z=0.0095410	Accept alternative hypothesis, data have a skewness
	p−value=0.9924	
Geary Kurtosis test	0.8643199≠0.7979	Accept alternative hypothesis
Anscombe-Glynn kurtosis test	kurtosis=1.8054,Z=−13.3610	Accept alternative hypothesis, kurtosis is not equal to 3
	p−value=0.0000	
Anderson-Darling test	p−value<0.001	Accept alternative hypothesis
Lilliefors-van Soest test	p−value<0.01	Accept alternative hypothesis
Cramer-von Mises test	p−value<0.005	Accept alternative hypothesis
Ryan-Joiner test	p−value<0.010	Accept alternative hypothesis

## 2 Experimental design, materials and methods

Primitive Pythagorean triples are one of the most popular number sequences in number theory which has been studied over time [1], [2], [3], [4], [5], [6], [7], [8], [9], [10], [11], [12].

### 2.1 Descriptive statistics

The mean, skewness, range and variance distribution was obtained for the first 331 terms of the sequence. The same statistics were obtained for the trigonometric, hyperbolic, logarithm, natural logarithm, exponential, digital root and iterative digits sum of the integers. Different data was obtained for each of the process. The descriptive analysis of the digital sum and iterative digits sum can be obtained from the analysis. Similar pattern of analysis of digits sum can be seen in [13], [14], [15], [16]. In addition, the algebraic properties were also analyzed.

### 2.2 Correlation

Three different types of correlation coefficient were computed for all integers at the different processes. They are; Pearson product moment correlation coefficient [17], Kendall's tau correlation coefficient [18] and Spearman rank correlation coefficient [19]. In addition, three dimensional scatter plots were obtained for all the difference between the integers that constitute the primitive Pythagorean triples.

### 2.3 Tests of normality

Normality tests were conducted for the integers a, b and c of the first 331 Primitive Pythagorean triples. Normality tests indicated non-normality but with different degrees. Normality tests used are: Kolmogorov-Smirnov test [20], Shapiro-Wilk test [21], Jarque-Bera Normality test [22], D’Agostino Skewness test [23], Geary Kurtosis test [24], Anscombe-Glynn kurtosis test [25], Anderson-Darling test [26], Lilliefors-van Soest test [27], [28], Cramer-von Mises test [29], and Ryan-Joiner test [30]. The summary of the analysis is available in [31].

Similar analysis can be obtained for the sum of digits of cubed integers, sum of winning integers in lotto and other numbers such as Fibonacci, Lucas, Happy, Weird, magic, Niven, Sophie Germain and so on [32], [33], [34], [35], [36], [37], [38], [39].
