# Supplementary material for: Datasets on the statistical and algebraic properties of primitive Pythagorean triples
Source: Data Brief. 2017 Sep 1;14:686–94. doi: 10.1016/j.dib.2017.08.021 (PMC5596336; doi:10.1016/j.dib.2017.08.021)
Supplement: Supplementary file 1 — Transparency document [file mmc2.zip › Supplementary Data 8.docx]

**Supplementary Data 8:** The tabulations of log and natural log of a, b and c and exponent of the inverse of a, b and c.

|  | log a | log b | log c | ln a | ln b | ln c | exp 1/a | exp 1/b | exp 1/c |
| --- | --- | --- | --- | --- | --- | --- | --- | --- | --- |
| 1 | 0.4771 | 0.6021 | 0.699 | 1.0986 | 1.3863 | 1.6094 | 1.3956 | 1.284 | 1.2214 |
| 2 | 0.699 | 1.0792 | 1.1139 | 1.6094 | 2.4849 | 2.5649 | 1.2214 | 1.0869 | 1.08 |
| 3 | 0.9031 | 1.1761 | 1.2304 | 2.0794 | 2.7081 | 2.8332 | 1.1331 | 1.0689 | 1.0606 |
| 4 | 0.8451 | 1.3802 | 1.3979 | 1.9459 | 3.1781 | 3.2189 | 1.1536 | 1.0425 | 1.0408 |
| 5 | 1.301 | 1.3222 | 1.4624 | 2.9957 | 3.0445 | 3.3673 | 1.0513 | 1.0488 | 1.0351 |
| 6 | 1.0792 | 1.5441 | 1.5682 | 2.4849 | 3.5553 | 3.6109 | 1.0869 | 1.029 | 1.0274 |
| 7 | 0.9542 | 1.6021 | 1.6128 | 2.1972 | 3.6889 | 3.7136 | 1.1175 | 1.0253 | 1.0247 |
| 8 | 1.4472 | 1.6532 | 1.7243 | 3.3322 | 3.8067 | 3.9703 | 1.0364 | 1.0225 | 1.019 |
| 9 | 1.0414 | 1.7782 | 1.7853 | 2.3979 | 4.0943 | 4.1109 | 1.0952 | 1.0168 | 1.0165 |
| 10 | 1.2041 | 1.7993 | 1.8129 | 2.7726 | 4.1431 | 4.1744 | 1.0645 | 1.016 | 1.0155 |
| 11 | 1.5185 | 1.7482 | 1.8129 | 3.4965 | 4.0254 | 4.1744 | 1.0308 | 1.018 | 1.0155 |
| 12 | 1.6812 | 1.7404 | 1.8633 | 3.8712 | 4.0073 | 4.2905 | 1.0211 | 1.0183 | 1.0138 |
| 13 | 1.1139 | 1.9243 | 1.9294 | 2.5649 | 4.4308 | 4.4427 | 1.08 | 1.012 | 1.0118 |
| 14 | 1.5563 | 1.8865 | 1.9294 | 3.5835 | 4.3438 | 4.4427 | 1.0282 | 1.0131 | 1.0118 |
| 15 | 1.5911 | 1.9031 | 1.9494 | 3.6636 | 4.382 | 4.4886 | 1.026 | 1.0126 | 1.0113 |
| 16 | 1.8129 | 1.8573 | 1.9868 | 4.1744 | 4.2767 | 4.5747 | 1.0155 | 1.014 | 1.0104 |
| 17 | 1.301 | 1.9956 | 2.0043 | 2.9957 | 4.5951 | 4.6151 | 1.0513 | 1.0102 | 1.01 |
| 18 | 1.7782 | 1.959 | 2.0374 | 4.0943 | 4.5109 | 4.6913 | 1.0168 | 1.011 | 1.0092 |
| 19 | 1.1761 | 2.0492 | 2.0531 | 2.7081 | 4.7185 | 4.7274 | 1.0689 | 1.009 | 1.0089 |
| 20 | 1.6435 | 2.0682 | 2.0969 | 3.7842 | 4.7622 | 4.8283 | 1.023 | 1.0086 | 1.008 |
| 21 | 1.9445 | 2.0212 | 2.1367 | 4.4773 | 4.654 | 4.92 | 1.0114 | 1.0096 | 1.0073 |
| 22 | 1.2304 | 2.1584 | 2.1614 | 2.8332 | 4.9698 | 4.9767 | 1.0606 | 1.007 | 1.0069 |
| 23 | 1.3802 | 2.1553 | 2.1614 | 3.1781 | 4.9628 | 4.9767 | 1.0425 | 1.007 | 1.0069 |
| 24 | 1.7076 | 2.1461 | 2.1732 | 3.9318 | 4.9416 | 5.0039 | 1.0198 | 1.0072 | 1.0067 |
| 25 | 1.9294 | 2.1206 | 2.1959 | 4.4427 | 4.8828 | 5.0562 | 1.0118 | 1.0076 | 1.0064 |
| 26 | 2.0755 | 2.0792 | 2.2279 | 4.7791 | 4.7875 | 5.1299 | 1.0084 | 1.0084 | 1.0059 |
| 27 | 1.716 | 2.2175 | 2.238 | 3.9512 | 5.1059 | 5.1533 | 1.0194 | 1.0061 | 1.0058 |
| 28 | 1.2788 | 2.2553 | 2.2577 | 2.9444 | 5.193 | 5.1985 | 1.054 | 1.0056 | 1.0055 |
| 29 | 1.7559 | 2.2455 | 2.2672 | 4.0431 | 5.1705 | 5.2204 | 1.0177 | 1.0057 | 1.0054 |
| 30 | 2.017 | 2.1847 | 2.2672 | 4.6444 | 5.0304 | 5.2204 | 1.0097 | 1.0066 | 1.0054 |
| 31 | 1.9777 | 2.2253 | 2.2856 | 4.5539 | 5.124 | 5.2627 | 1.0106 | 1.006 | 1.0052 |
| 32 | 1.4472 | 2.29 | 2.2945 | 3.3322 | 5.273 | 5.2832 | 1.0364 | 1.0051 | 1.0051 |
| 33 | 1.9243 | 2.2718 | 2.3118 | 4.4308 | 5.2311 | 5.323 | 1.012 | 1.0054 | 1.0049 |
| 34 | 2.1239 | 2.1931 | 2.3118 | 4.8903 | 5.0499 | 5.323 | 1.0075 | 1.0064 | 1.0049 |
| 35 | 1.3222 | 2.3424 | 2.3444 | 3.0445 | 5.3936 | 5.3982 | 1.0488 | 1.0046 | 1.0045 |
| 36 | 2.1461 | 2.233 | 2.3444 | 4.9416 | 5.1417 | 5.3982 | 1.0072 | 1.0059 | 1.0045 |
| 37 | 1.7782 | 2.3444 | 2.3598 | 4.0943 | 5.3982 | 5.4337 | 1.0168 | 1.0045 | 1.0044 |
| 38 | 2.0212 | 2.3181 | 2.3674 | 4.654 | 5.3375 | 5.451 | 1.0096 | 1.0048 | 1.0043 |
| 39 | 2.0792 | 2.3201 | 2.382 | 4.7875 | 5.3423 | 5.4848 | 1.0084 | 1.0048 | 1.0042 |
| 40 | 1.5051 | 2.4065 | 2.4099 | 3.4657 | 5.5413 | 5.5491 | 1.0317 | 1.0039 | 1.0039 |
| 41 | 1.3617 | 2.4216 | 2.4232 | 3.1355 | 5.5759 | 5.5797 | 1.0444 | 1.0038 | 1.0038 |
| 42 | 1.9823 | 2.3927 | 2.4232 | 4.5643 | 5.5094 | 5.5797 | 1.0105 | 1.0041 | 1.0038 |
| 43 | 1.8388 | 2.415 | 2.4298 | 4.2341 | 5.5607 | 5.5947 | 1.0146 | 1.0039 | 1.0037 |
| 44 | 2.0607 | 2.4014 | 2.4425 | 4.7449 | 5.5294 | 5.624 | 1.0087 | 1.004 | 1.0036 |
| 45 | 2.2041 | 2.3636 | 2.4487 | 5.0752 | 5.4424 | 5.6384 | 1.0063 | 1.0043 | 1.0036 |
| 46 | 2.2068 | 2.3802 | 2.4609 | 5.0814 | 5.4806 | 5.6664 | 1.0062 | 1.0042 | 1.0035 |
| 47 | 1.8325 | 2.4548 | 2.4669 | 4.2195 | 5.6525 | 5.6802 | 1.0148 | 1.0035 | 1.0034 |
| 48 | 2.1335 | 2.4362 | 2.4843 | 4.9127 | 5.6095 | 5.7203 | 1.0074 | 1.0037 | 1.0033 |
| 49 | 2.316 | 2.3502 | 2.4843 | 5.3327 | 5.4116 | 5.7203 | 1.0048 | 1.0045 | 1.0033 |
| 50 | 1.3979 | 2.4942 | 2.4955 | 3.2189 | 5.743 | 5.7462 | 1.0408 | 1.0032 | 1.0032 |
| 51 | 1.8751 | 2.4886 | 2.5011 | 4.3175 | 5.7301 | 5.7589 | 1.0134 | 1.0033 | 1.0032 |
| 52 | 1.5563 | 2.5092 | 2.5119 | 3.5835 | 5.7777 | 5.7838 | 1.0282 | 1.0031 | 1.0031 |
| 53 | 2.3096 | 2.4031 | 2.5119 | 5.3181 | 5.5334 | 5.7838 | 1.0049 | 1.004 | 1.0031 |
| 54 | 2.243 | 2.4594 | 2.5276 | 5.1648 | 5.663 | 5.8201 | 1.0057 | 1.0035 | 1.003 |
| 55 | 2.2553 | 2.4757 | 2.5428 | 5.193 | 5.7004 | 5.8551 | 1.0056 | 1.0034 | 1.0029 |
| 56 | 2.3522 | 2.4346 | 2.5478 | 5.4161 | 5.6058 | 5.8665 | 1.0045 | 1.0037 | 1.0028 |
| 57 | 1.4314 | 2.5611 | 2.5623 | 3.2958 | 5.8972 | 5.8999 | 1.0377 | 1.0028 | 1.0027 |
| 58 | 1.8808 | 2.5527 | 2.5623 | 4.3307 | 5.8777 | 5.8999 | 1.0132 | 1.0028 | 1.0027 |
| 59 | 2.4014 | 2.4393 | 2.5717 | 5.5294 | 5.6168 | 5.9216 | 1.004 | 1.0036 | 1.0027 |
| 60 | 2.1303 | 2.5465 | 2.5763 | 4.9053 | 5.8636 | 5.9322 | 1.0074 | 1.0028 | 1.0027 |
| 61 | 2.1818 | 2.5378 | 2.5763 | 5.0239 | 5.8435 | 5.9322 | 1.0066 | 1.0029 | 1.0027 |
| 62 | 2.2765 | 2.5315 | 2.5899 | 5.2417 | 5.8289 | 5.9636 | 1.0053 | 1.0029 | 1.0026 |
| 63 | 2.3579 | 2.5119 | 2.5988 | 5.4293 | 5.7838 | 5.9839 | 1.0044 | 1.0031 | 1.0025 |
| 64 | 1.6021 | 2.601 | 2.6031 | 3.6889 | 5.989 | 5.994 | 1.0253 | 1.0025 | 1.0025 |
| 65 | 2.0792 | 2.5922 | 2.6117 | 4.7875 | 5.9687 | 6.0137 | 1.0084 | 1.0026 | 1.0024 |
| 66 | 1.4624 | 2.6232 | 2.6243 | 3.3673 | 6.0403 | 6.0426 | 1.0351 | 1.0024 | 1.0024 |
| 67 | 1.9395 | 2.6191 | 2.6284 | 4.4659 | 6.0307 | 6.0521 | 1.0116 | 1.0024 | 1.0024 |
| 68 | 2.4728 | 2.4829 | 2.6284 | 5.6937 | 5.717 | 6.0521 | 1.0034 | 1.0033 | 1.0024 |
| 69 | 2.1614 | 2.6107 | 2.6365 | 4.9767 | 6.0113 | 6.0707 | 1.0069 | 1.0025 | 1.0023 |
| 70 | 1.9243 | 2.6405 | 2.6484 | 4.4308 | 6.0799 | 6.0981 | 1.012 | 1.0023 | 1.0022 |
| 71 | 2.3075 | 2.5977 | 2.6484 | 5.3132 | 5.9814 | 6.0981 | 1.0049 | 1.0025 | 1.0022 |
| 72 | 2.4472 | 2.5453 | 2.6522 | 5.6348 | 5.8608 | 6.107 | 1.0036 | 1.0029 | 1.0022 |
| 73 | 2.2253 | 2.6284 | 2.6599 | 5.124 | 6.0521 | 6.1247 | 1.006 | 1.0024 | 1.0022 |
| 74 | 2.4166 | 2.5798 | 2.6637 | 5.5645 | 5.9402 | 6.1334 | 1.0038 | 1.0026 | 1.0022 |
| 75 | 1.4914 | 2.6812 | 2.6821 | 3.434 | 6.1738 | 6.1759 | 1.0328 | 1.0021 | 1.0021 |
| 76 | 2.5038 | 2.5563 | 2.6821 | 5.7652 | 5.8861 | 6.1759 | 1.0031 | 1.0028 | 1.0021 |
| 77 | 1.6435 | 2.6839 | 2.6857 | 3.7842 | 6.18 | 6.1841 | 1.023 | 1.0021 | 1.0021 |
| 78 | 1.9685 | 2.6776 | 2.6857 | 4.5326 | 6.1654 | 6.1841 | 1.0108 | 1.0021 | 1.0021 |
| 79 | 2.1206 | 2.6767 | 2.6928 | 4.8828 | 6.1633 | 6.2005 | 1.0076 | 1.0021 | 1.002 |
| 80 | 2.1903 | 2.6702 | 2.6928 | 5.0434 | 6.1485 | 6.2005 | 1.0065 | 1.0021 | 1.002 |
| 81 | 2.3365 | 2.659 | 2.7033 | 5.3799 | 6.1225 | 6.2246 | 1.0046 | 1.0022 | 1.002 |
| 82 | 2.5263 | 2.5763 | 2.7033 | 5.8171 | 5.9322 | 6.2246 | 1.003 | 1.0027 | 1.002 |
| 83 | 2.3424 | 2.6618 | 2.7067 | 5.3936 | 6.1291 | 6.2324 | 1.0046 | 1.0022 | 1.002 |
| 84 | 2.4456 | 2.6435 | 2.7168 | 5.6312 | 6.0868 | 6.2558 | 1.0036 | 1.0023 | 1.0019 |
| 85 | 1.9638 | 2.7202 | 2.7267 | 4.5218 | 6.2634 | 6.2785 | 1.0109 | 1.0019 | 1.0019 |
| 86 | 2.4886 | 2.6385 | 2.7267 | 5.7301 | 6.0753 | 6.2785 | 1.0033 | 1.0023 | 1.0019 |
| 87 | 2.5328 | 2.6232 | 2.7332 | 5.8319 | 6.0403 | 6.2934 | 1.0029 | 1.0024 | 1.0019 |
| 88 | 1.5185 | 2.7356 | 2.7364 | 3.4965 | 6.2989 | 6.3008 | 1.0308 | 1.0018 | 1.0018 |
| 89 | 2.2648 | 2.7101 | 2.7364 | 5.2149 | 6.2403 | 6.3008 | 1.0054 | 1.002 | 1.0018 |
| 90 | 2.2175 | 2.7259 | 2.7459 | 5.1059 | 6.2766 | 6.3226 | 1.0061 | 1.0019 | 1.0018 |
| 91 | 2.4409 | 2.6928 | 2.752 | 5.6204 | 6.2005 | 6.3368 | 1.0036 | 1.002 | 1.0018 |
| 92 | 2.5977 | 2.6053 | 2.752 | 5.9814 | 5.9989 | 6.3368 | 1.0025 | 1.0025 | 1.0018 |
| 93 | 2.3636 | 2.716 | 2.7551 | 5.4424 | 6.2538 | 6.3439 | 1.0043 | 1.0019 | 1.0018 |
| 94 | 1.6812 | 2.7597 | 2.7612 | 3.8712 | 6.3544 | 6.3578 | 1.0211 | 1.0017 | 1.0017 |
| 95 | 2.5658 | 2.6675 | 2.7731 | 5.9081 | 6.142 | 6.3852 | 1.0027 | 1.0022 | 1.0017 |
| 96 | 2.3802 | 2.7412 | 2.7789 | 5.4806 | 6.3117 | 6.3986 | 1.0042 | 1.0018 | 1.0017 |
| 97 | 1.5441 | 2.7868 | 2.7875 | 3.5553 | 6.4167 | 6.4184 | 1.029 | 1.0016 | 1.0016 |
| 98 | 2.0212 | 2.7839 | 2.7903 | 4.654 | 6.4102 | 6.4249 | 1.0096 | 1.0016 | 1.0016 |
| 99 | 2.5263 | 2.7218 | 2.7959 | 5.8171 | 6.2672 | 6.4378 | 1.003 | 1.0019 | 1.0016 |
| 100 | 2 | 2.7931 | 2.7987 | 4.6052 | 6.4313 | 6.4441 | 1.0101 | 1.0016 | 1.0016 |
| 101 | 2.6325 | 2.6628 | 2.7987 | 6.0615 | 6.1312 | 6.4441 | 1.0023 | 1.0022 | 1.0016 |
| 102 | 2.301 | 2.7846 | 2.8069 | 5.2983 | 6.4118 | 6.463 | 1.005 | 1.0016 | 1.0016 |
| 103 | 2.4983 | 2.7574 | 2.8149 | 5.7526 | 6.3491 | 6.4816 | 1.0032 | 1.0017 | 1.0015 |
| 104 | 2.4771 | 2.7701 | 2.8202 | 5.7038 | 6.3784 | 6.4938 | 1.0033 | 1.0017 | 1.0015 |
| 105 | 2.5855 | 2.7419 | 2.828 | 5.9532 | 6.3135 | 6.5117 | 1.0026 | 1.0018 | 1.0015 |
| 106 | 1.716 | 2.8293 | 2.8306 | 3.9512 | 6.5147 | 6.5177 | 1.0194 | 1.0015 | 1.0015 |
| 107 | 1.5682 | 2.8351 | 2.8357 | 3.6109 | 6.528 | 6.5294 | 1.0274 | 1.0015 | 1.0015 |
| 108 | 2.1931 | 2.8241 | 2.8357 | 5.0499 | 6.5028 | 6.5294 | 1.0064 | 1.0015 | 1.0015 |
| 109 | 2.0453 | 2.8325 | 2.8382 | 4.7095 | 6.5221 | 6.5352 | 1.009 | 1.0015 | 1.0015 |
| 110 | 2.6021 | 2.749 | 2.8382 | 5.9915 | 6.3297 | 6.5352 | 1.0025 | 1.0018 | 1.0015 |
| 111 | 2.2672 | 2.8274 | 2.8432 | 5.2204 | 6.5103 | 6.5468 | 1.0054 | 1.0015 | 1.0014 |
| 112 | 2.658 | 2.7226 | 2.8432 | 6.1203 | 6.2691 | 6.5468 | 1.0022 | 1.0019 | 1.0014 |
| 113 | 2.415 | 2.8136 | 2.8457 | 5.5607 | 6.4785 | 6.5525 | 1.0039 | 1.0015 | 1.0014 |
| 114 | 2.4133 | 2.8195 | 2.8506 | 5.5568 | 6.4922 | 6.5639 | 1.0039 | 1.0015 | 1.0014 |
| 115 | 2.5224 | 2.8089 | 2.8603 | 5.8081 | 6.4677 | 6.5862 | 1.003 | 1.0016 | 1.0014 |
| 116 | 2.5611 | 2.7973 | 2.8603 | 5.8972 | 6.4409 | 6.5862 | 1.0028 | 1.0016 | 1.0014 |
| 117 | 2.0334 | 2.8603 | 2.8651 | 4.6821 | 6.5862 | 6.5971 | 1.0093 | 1.0014 | 1.0014 |
| 118 | 2.3345 | 2.8531 | 2.8722 | 5.3753 | 6.5695 | 6.6134 | 1.0046 | 1.0014 | 1.0013 |
| 119 | 2.6096 | 2.7952 | 2.8722 | 6.0088 | 6.4362 | 6.6134 | 1.0025 | 1.0016 | 1.0013 |
| 120 | 2.6702 | 2.7745 | 2.8791 | 6.1485 | 6.3886 | 6.6294 | 1.0021 | 1.0017 | 1.0013 |
| 121 | 1.5911 | 2.8808 | 2.8814 | 3.6636 | 6.6333 | 6.6346 | 1.026 | 1.0013 | 1.0013 |
| 122 | 2.6821 | 2.7782 | 2.8859 | 6.1759 | 6.3969 | 6.6451 | 1.0021 | 1.0017 | 1.0013 |
| 123 | 2.29 | 2.8739 | 2.8882 | 5.273 | 6.6174 | 6.6503 | 1.0051 | 1.0013 | 1.0013 |
| 124 | 1.7482 | 2.8938 | 2.8949 | 4.0254 | 6.6631 | 6.6657 | 1.018 | 1.0013 | 1.0013 |
| 125 | 2.4362 | 2.8669 | 2.8949 | 5.6095 | 6.6012 | 6.6657 | 1.0037 | 1.0014 | 1.0013 |
| 126 | 2.2253 | 2.8893 | 2.8993 | 5.124 | 6.6529 | 6.6758 | 1.006 | 1.0013 | 1.0013 |
| 127 | 2.6355 | 2.8228 | 2.8993 | 6.0684 | 6.4998 | 6.6758 | 1.0023 | 1.0015 | 1.0013 |
| 128 | 2.7443 | 2.7574 | 2.9015 | 6.319 | 6.3491 | 6.6809 | 1.0018 | 1.0017 | 1.0013 |
| 129 | 2.4472 | 2.8802 | 2.9079 | 5.6348 | 6.632 | 6.6958 | 1.0036 | 1.0013 | 1.0012 |
| 130 | 2.6325 | 2.8451 | 2.9143 | 6.0615 | 6.5511 | 6.7105 | 1.0023 | 1.0014 | 1.0012 |
| 131 | 2.7324 | 2.7987 | 2.9186 | 6.2916 | 6.4441 | 6.7202 | 1.0019 | 1.0016 | 1.0012 |
| 132 | 1.6128 | 2.9243 | 2.9248 | 3.7136 | 6.7334 | 6.7346 | 1.0247 | 1.0012 | 1.0012 |
| 133 | 2.0645 | 2.9227 | 2.9269 | 4.7536 | 6.7298 | 6.7393 | 1.0087 | 1.0012 | 1.0012 |
| 134 | 2.0899 | 2.9222 | 2.9269 | 4.8122 | 6.7286 | 6.7393 | 1.0082 | 1.0012 | 1.0012 |
| 135 | 2.3118 | 2.918 | 2.9309 | 5.323 | 6.719 | 6.7488 | 1.0049 | 1.0012 | 1.0012 |
| 136 | 2.3655 | 2.9165 | 2.933 | 5.4467 | 6.7154 | 6.7534 | 1.0043 | 1.0012 | 1.0012 |
| 137 | 2.4579 | 2.9117 | 2.937 | 5.6595 | 6.7044 | 6.7627 | 1.0035 | 1.0012 | 1.0012 |
| 138 | 2.7024 | 2.847 | 2.937 | 6.2226 | 6.5554 | 6.7627 | 1.002 | 1.0014 | 1.0012 |
| 139 | 2.5416 | 2.9058 | 2.943 | 5.8522 | 6.6908 | 6.7765 | 1.0029 | 1.0012 | 1.0011 |
| 140 | 2.567 | 2.9031 | 2.945 | 5.9108 | 6.6846 | 6.7811 | 1.0027 | 1.0013 | 1.0011 |
| 141 | 1.7782 | 2.9538 | 2.9547 | 4.0943 | 6.8013 | 6.8035 | 1.0168 | 1.0011 | 1.0011 |
| 142 | 2.6542 | 2.8921 | 2.9547 | 6.1115 | 6.6593 | 6.8035 | 1.0022 | 1.0013 | 1.0011 |
| 143 | 2.6665 | 2.8904 | 2.9566 | 6.1399 | 6.6554 | 6.8079 | 1.0022 | 1.0013 | 1.0011 |
| 144 | 2.7896 | 2.8215 | 2.9566 | 6.4232 | 6.4968 | 6.8079 | 1.0016 | 1.0015 | 1.0011 |
| 145 | 1.6335 | 2.9657 | 2.9661 | 3.7612 | 6.8287 | 6.8298 | 1.0235 | 1.0011 | 1.0011 |
| 146 | 2.7267 | 2.8785 | 2.9661 | 6.2785 | 6.628 | 6.8298 | 1.0019 | 1.0013 | 1.0011 |
| 147 | 2.1106 | 2.9638 | 2.968 | 4.8598 | 6.8244 | 6.8341 | 1.0078 | 1.0011 | 1.0011 |
| 148 | 2.3324 | 2.96 | 2.9717 | 5.3706 | 6.8156 | 6.8427 | 1.0047 | 1.0011 | 1.0011 |
| 149 | 2.7634 | 2.8698 | 2.9736 | 6.363 | 6.608 | 6.8469 | 1.0017 | 1.0014 | 1.0011 |
| 150 | 2.4786 | 2.9542 | 2.9773 | 5.7071 | 6.8024 | 6.8554 | 1.0033 | 1.0011 | 1.0011 |
| 151 | 2.6232 | 2.9299 | 2.9773 | 6.0403 | 6.7464 | 6.8554 | 1.0024 | 1.0012 | 1.0011 |
| 152 | 2.7889 | 2.8621 | 2.9791 | 6.4216 | 6.5903 | 6.8596 | 1.0016 | 1.0014 | 1.001 |
| 153 | 2.0934 | 2.9809 | 2.9845 | 4.8203 | 6.8638 | 6.8721 | 1.0081 | 1.001 | 1.001 |
| 154 | 2.5877 | 2.9465 | 2.9845 | 5.9584 | 6.7845 | 6.8721 | 1.0026 | 1.0011 | 1.001 |
| 155 | 2.3945 | 2.9754 | 2.9899 | 5.5134 | 6.8512 | 6.8845 | 1.004 | 1.0011 | 1.001 |
| 156 | 2.6749 | 2.9365 | 2.9934 | 6.1591 | 6.7616 | 6.8926 | 1.0021 | 1.0012 | 1.001 |
| 157 | 2.8426 | 2.8432 | 2.9934 | 6.5453 | 6.5468 | 6.8926 | 1.0014 | 1.0014 | 1.001 |
| 158 | 2.5705 | 2.9661 | 2.9987 | 5.9189 | 6.8298 | 6.9048 | 1.0027 | 1.0011 | 1.001 |
| 159 | 2.7474 | 2.9243 | 3.0039 | 6.3261 | 6.7334 | 6.9167 | 1.0018 | 1.0012 | 1.001 |
| 160 | 1.6532 | 3.0052 | 3.0056 | 3.8067 | 6.9197 | 6.9207 | 1.0225 | 1.001 | 1.001 |
| 161 | 2.8195 | 2.8915 | 3.009 | 6.4922 | 6.658 | 6.9285 | 1.0015 | 1.0013 | 1.001 |
| 162 | 1.8062 | 3.0099 | 3.0107 | 4.1589 | 6.9305 | 6.9324 | 1.0157 | 1.001 | 1.001 |
| 163 | 2.6955 | 2.9528 | 3.0107 | 6.2066 | 6.7991 | 6.9324 | 1.002 | 1.0011 | 1.001 |
| 164 | 2.2833 | 3.0065 | 3.0141 | 5.2575 | 6.9226 | 6.9402 | 1.0052 | 1.001 | 1.001 |
| 165 | 2.4983 | 2.9948 | 3.0158 | 5.7526 | 6.8957 | 6.9441 | 1.0032 | 1.001 | 1.001 |
| 166 | 2.8096 | 2.9096 | 3.0158 | 6.4693 | 6.6995 | 6.9441 | 1.0016 | 1.0012 | 1.001 |
| 167 | 2.5051 | 2.9996 | 3.0208 | 5.7683 | 6.9068 | 6.9556 | 1.0031 | 1.001 | 1.001 |
| 168 | 2.7924 | 2.935 | 3.0257 | 6.4297 | 6.7581 | 6.967 | 1.0016 | 1.0012 | 1.0009 |
| 169 | 2.8639 | 2.8921 | 3.029 | 6.5944 | 6.6593 | 6.9745 | 1.0014 | 1.0013 | 1.0009 |
| 170 | 2.6513 | 2.989 | 3.0306 | 6.1048 | 6.8824 | 6.9782 | 1.0022 | 1.001 | 1.0009 |
| 171 | 2.6946 | 2.9786 | 3.0306 | 6.2046 | 6.8586 | 6.9782 | 1.002 | 1.0011 | 1.0009 |
| 172 | 2.1206 | 3.0354 | 3.0386 | 4.8828 | 6.9893 | 6.9967 | 1.0076 | 1.0009 | 1.0009 |
| 173 | 2.7672 | 2.9675 | 3.0402 | 6.3716 | 6.833 | 7.0003 | 1.0017 | 1.0011 | 1.0009 |
| 174 | 1.6721 | 3.043 | 3.0434 | 3.8501 | 7.0067 | 7.0076 | 1.0215 | 1.0009 | 1.0009 |
| 175 | 2.4216 | 3.0306 | 3.0434 | 5.5759 | 6.9782 | 7.0076 | 1.0038 | 1.0009 | 1.0009 |
| 176 | 2.7604 | 2.9745 | 3.0434 | 6.3561 | 6.8491 | 7.0076 | 1.0017 | 1.0011 | 1.0009 |
| 177 | 2.8716 | 2.9122 | 3.0434 | 6.612 | 6.7056 | 7.0076 | 1.0013 | 1.0012 | 1.0009 |
| 178 | 2.1492 | 3.0414 | 3.0449 | 4.9488 | 7.0031 | 7.0112 | 1.0071 | 1.0009 | 1.0009 |
| 179 | 2.3711 | 3.0382 | 3.0481 | 5.4596 | 6.9958 | 7.0184 | 1.0043 | 1.0009 | 1.0009 |
| 180 | 2.5172 | 3.0334 | 3.0527 | 5.7961 | 6.9847 | 7.0291 | 1.003 | 1.0009 | 1.0009 |
| 181 | 2.6263 | 3.0269 | 3.0588 | 6.0474 | 6.9698 | 7.0432 | 1.0024 | 1.0009 | 1.0009 |
| 182 | 2.8476 | 2.9557 | 3.0588 | 6.5568 | 6.8057 | 7.0432 | 1.0014 | 1.0011 | 1.0009 |
| 183 | 2.7226 | 3.0107 | 3.0618 | 6.2691 | 6.9324 | 7.0501 | 1.0019 | 1.001 | 1.0009 |
| 184 | 1.8325 | 3.0626 | 3.0633 | 4.2195 | 7.0519 | 7.0536 | 1.0148 | 1.0009 | 1.0009 |
| 185 | 2.8837 | 2.9385 | 3.0633 | 6.6399 | 6.7662 | 7.0536 | 1.0013 | 1.0012 | 1.0009 |
| 186 | 2.3096 | 3.0596 | 3.0663 | 5.3181 | 7.0449 | 7.0605 | 1.0049 | 1.0009 | 1.0009 |
| 187 | 2.7135 | 3.0187 | 3.0663 | 6.248 | 6.9508 | 7.0605 | 1.0019 | 1.001 | 1.0009 |
| 188 | 2.5315 | 3.0535 | 3.0722 | 5.8289 | 7.0309 | 7.0741 | 1.0029 | 1.0009 | 1.0008 |
| 189 | 2.786 | 3.0086 | 3.0752 | 6.4151 | 6.9276 | 7.0809 | 1.0016 | 1.001 | 1.0008 |
| 190 | 2.8195 | 2.9952 | 3.0752 | 6.4922 | 6.8967 | 7.0809 | 1.0015 | 1.001 | 1.0008 |
| 191 | 2.9201 | 2.932 | 3.0766 | 6.7238 | 6.7511 | 7.0842 | 1.0012 | 1.0012 | 1.0008 |
| 192 | 1.6902 | 3.0792 | 3.0795 | 3.8918 | 7.0901 | 7.0909 | 1.0206 | 1.0008 | 1.0008 |
| 193 | 2.1673 | 3.0777 | 3.081 | 4.9904 | 7.0867 | 7.0942 | 1.0068 | 1.0008 | 1.0008 |
| 194 | 2.6776 | 3.0441 | 3.081 | 6.1654 | 7.0094 | 7.0942 | 1.0021 | 1.0009 | 1.0008 |
| 195 | 2.3892 | 3.0748 | 3.0839 | 5.5013 | 7.08 | 7.1009 | 1.0041 | 1.0008 | 1.0008 |
| 196 | 2.8482 | 2.9965 | 3.0853 | 6.5582 | 6.8997 | 7.1041 | 1.0014 | 1.001 | 1.0008 |
| 197 | 2.1461 | 3.0867 | 3.0896 | 4.9416 | 7.1074 | 7.114 | 1.0072 | 1.0008 | 1.0008 |
| 198 | 2.7868 | 3.0314 | 3.0924 | 6.4167 | 6.9801 | 7.1204 | 1.0016 | 1.0009 | 1.0008 |
| 199 | 2.4472 | 3.0824 | 3.0938 | 5.6348 | 7.0975 | 7.1237 | 1.0036 | 1.0008 | 1.0008 |
| 200 | 2.6444 | 3.0645 | 3.0938 | 6.089 | 7.0562 | 7.1237 | 1.0023 | 1.0009 | 1.0008 |
| 201 | 2.9025 | 2.9823 | 3.0966 | 6.6834 | 6.8669 | 7.1301 | 1.0013 | 1.001 | 1.0008 |
| 202 | 2.6232 | 3.0752 | 3.1007 | 6.0403 | 7.0809 | 7.1397 | 1.0024 | 1.0008 | 1.0008 |
| 203 | 2.7316 | 3.0569 | 3.1007 | 6.2897 | 7.0388 | 7.1397 | 1.0019 | 1.0009 | 1.0008 |
| 204 | 2.8739 | 3.0149 | 3.1062 | 6.6174 | 6.9422 | 7.1523 | 1.0013 | 1.001 | 1.0008 |
| 205 | 2.8041 | 3.0477 | 3.1089 | 6.4568 | 7.0175 | 7.1585 | 1.0016 | 1.0009 | 1.0008 |
| 206 | 2.9509 | 2.9657 | 3.1089 | 6.7946 | 6.8287 | 7.1585 | 1.0011 | 1.0011 | 1.0008 |
| 207 | 2.7482 | 3.0648 | 3.1103 | 6.3279 | 7.057 | 7.1616 | 1.0018 | 1.0009 | 1.0008 |
| 208 | 1.8573 | 3.1123 | 3.1129 | 4.2767 | 7.1663 | 7.1678 | 1.014 | 1.0008 | 1.0008 |
| 209 | 1.7076 | 3.1139 | 3.1143 | 3.9318 | 7.1701 | 7.1709 | 1.0198 | 1.0008 | 1.0008 |
| 210 | 2.4065 | 3.1099 | 3.1183 | 5.5413 | 7.1608 | 7.1801 | 1.0039 | 1.0008 | 1.0008 |
| 211 | 2.8663 | 3.0366 | 3.1183 | 6.5999 | 6.9921 | 7.1801 | 1.0014 | 1.0009 | 1.0008 |
| 212 | 2.5563 | 3.1041 | 3.1209 | 5.8861 | 7.1476 | 7.1861 | 1.0028 | 1.0008 | 1.0008 |
| 213 | 2.5527 | 3.1059 | 3.1222 | 5.8777 | 7.1515 | 7.1892 | 1.0028 | 1.0008 | 1.0008 |
| 214 | 2.9465 | 2.9943 | 3.1222 | 6.7845 | 6.8947 | 7.1892 | 1.0011 | 1.001 | 1.0008 |
| 215 | 2.7024 | 3.0959 | 3.1287 | 6.2226 | 7.1285 | 7.2041 | 1.002 | 1.0008 | 1.0007 |
| 216 | 2.9206 | 3.0237 | 3.1287 | 6.725 | 6.9622 | 7.2041 | 1.0012 | 1.0009 | 1.0007 |
| 217 | 2.749 | 3.0934 | 3.1339 | 6.3297 | 7.1229 | 7.216 | 1.0018 | 1.0008 | 1.0007 |
| 218 | 2.9243 | 3.0338 | 3.1364 | 6.7334 | 6.9856 | 7.2218 | 1.0012 | 1.0009 | 1.0007 |
| 219 | 2.1703 | 3.1351 | 3.1377 | 4.9972 | 7.2189 | 7.2248 | 1.0068 | 1.0007 | 1.0007 |
| 220 | 2.9689 | 3.0086 | 3.1402 | 6.8363 | 6.9276 | 7.2306 | 1.0011 | 1.001 | 1.0007 |
| 221 | 2.4713 | 3.1313 | 3.1414 | 5.6904 | 7.2101 | 7.2335 | 1.0034 | 1.0007 | 1.0007 |
| 222 | 2.8215 | 3.0849 | 3.1414 | 6.4968 | 7.1033 | 7.2335 | 1.0015 | 1.0008 | 1.0007 |
| 223 | 1.7243 | 3.1474 | 3.1477 | 3.9703 | 7.2471 | 7.2478 | 1.019 | 1.0007 | 1.0007 |
| 224 | 2.6474 | 3.1248 | 3.1477 | 6.0958 | 7.1952 | 7.2478 | 1.0023 | 1.0008 | 1.0007 |
| 225 | 2.2014 | 3.1461 | 3.1489 | 5.0689 | 7.2442 | 7.2506 | 1.0063 | 1.0007 | 1.0007 |
| 226 | 2.4232 | 3.1436 | 3.1514 | 5.5797 | 7.2385 | 7.2563 | 1.0038 | 1.0007 | 1.0007 |
| 227 | 2.8987 | 3.07 | 3.1514 | 6.6746 | 7.069 | 7.2563 | 1.0013 | 1.0009 | 1.0007 |
| 228 | 2.5694 | 3.1399 | 3.155 | 5.9162 | 7.2298 | 7.2647 | 1.0027 | 1.0007 | 1.0007 |
| 229 | 2.7723 | 3.1156 | 3.1562 | 6.3835 | 7.174 | 7.2675 | 1.0017 | 1.0008 | 1.0007 |
| 230 | 1.8808 | 3.1593 | 3.1599 | 4.3307 | 7.2745 | 7.2759 | 1.0132 | 1.0007 | 1.0007 |
| 231 | 2.6785 | 3.1348 | 3.1599 | 6.1675 | 7.2182 | 7.2759 | 1.0021 | 1.0007 | 1.0007 |
| 232 | 2.3579 | 3.1569 | 3.1623 | 5.4293 | 7.2689 | 7.2814 | 1.0044 | 1.0007 | 1.0007 |
| 233 | 2.7657 | 3.1284 | 3.1658 | 6.3682 | 7.2034 | 7.2896 | 1.0017 | 1.0007 | 1.0007 |
| 234 | 2.9713 | 3.0519 | 3.1658 | 6.8416 | 7.0273 | 7.2896 | 1.0011 | 1.0009 | 1.0007 |
| 235 | 2.5798 | 3.152 | 3.167 | 5.9402 | 7.2577 | 7.2923 | 1.0026 | 1.0007 | 1.0007 |
| 236 | 2.8692 | 3.1035 | 3.167 | 6.6067 | 7.146 | 7.2923 | 1.0014 | 1.0008 | 1.0007 |
| 237 | 2.9863 | 3.0492 | 3.1706 | 6.8763 | 7.0211 | 7.3005 | 1.001 | 1.0009 | 1.0007 |
| 238 | 2.8382 | 3.1206 | 3.1729 | 6.5352 | 7.1854 | 7.3059 | 1.0015 | 1.0008 | 1.0007 |
| 239 | 2.7259 | 3.1446 | 3.1741 | 6.2766 | 7.2406 | 7.3085 | 1.0019 | 1.0007 | 1.0007 |
| 240 | 1.7404 | 3.1796 | 3.1798 | 4.0073 | 7.3212 | 7.3218 | 1.0183 | 1.0007 | 1.0007 |
| 241 | 2.9484 | 3.0881 | 3.1798 | 6.789 | 7.1107 | 7.3218 | 1.0011 | 1.0008 | 1.0007 |
| 242 | 2.2175 | 3.1784 | 3.181 | 5.1059 | 7.3185 | 7.3245 | 1.0061 | 1.0007 | 1.0007 |
| 243 | 2.9004 | 3.1113 | 3.181 | 6.6783 | 7.1639 | 7.3245 | 1.0013 | 1.0008 | 1.0007 |
| 244 | 2.1931 | 3.181 | 3.1833 | 5.0499 | 7.3245 | 7.3297 | 1.0064 | 1.0007 | 1.0007 |
| 245 | 2.8351 | 3.1345 | 3.1833 | 6.528 | 7.2174 | 7.3297 | 1.0015 | 1.0007 | 1.0007 |
| 246 | 2.4942 | 3.1775 | 3.1867 | 5.743 | 7.3165 | 7.3376 | 1.0032 | 1.0007 | 1.0007 |
| 247 | 2.5855 | 3.1726 | 3.1867 | 5.9532 | 7.3052 | 7.3376 | 1.0026 | 1.0007 | 1.0007 |
| 248 | 2.9547 | 3.1004 | 3.1901 | 6.8035 | 7.1389 | 7.3454 | 1.0011 | 1.0008 | 1.0006 |
| 249 | 2.6946 | 3.1679 | 3.1912 | 6.2046 | 7.2944 | 7.3479 | 1.002 | 1.0007 | 1.0006 |
| 250 | 2.9222 | 3.1216 | 3.1945 | 6.7286 | 7.1877 | 7.3556 | 1.0012 | 1.0008 | 1.0006 |
| 251 | 3.0154 | 3.0693 | 3.1945 | 6.9431 | 7.0673 | 7.3556 | 1.001 | 1.0009 | 1.0006 |
| 252 | 2.7952 | 3.1635 | 3.2 | 6.4362 | 7.2841 | 7.3683 | 1.0016 | 1.0007 | 1.0006 |
| 253 | 3.003 | 3.0878 | 3.2 | 6.9147 | 7.1099 | 7.3683 | 1.001 | 1.0008 | 1.0006 |
| 254 | 2.8543 | 3.1547 | 3.2033 | 6.5723 | 7.264 | 7.3759 | 1.0014 | 1.0007 | 1.0006 |
| 255 | 1.9031 | 3.2038 | 3.2044 | 4.382 | 7.3771 | 7.3784 | 1.0126 | 1.0006 | 1.0006 |
| 256 | 2.3802 | 3.2017 | 3.2066 | 5.4806 | 7.3721 | 7.3834 | 1.0042 | 1.0006 | 1.0006 |
| 257 | 2.9948 | 3.1055 | 3.2076 | 6.8957 | 7.1507 | 7.3859 | 1.001 | 1.0008 | 1.0006 |
| 258 | 2.8921 | 3.1526 | 3.2098 | 6.6593 | 7.2591 | 7.3908 | 1.0013 | 1.0007 | 1.0006 |
| 259 | 1.7559 | 3.2106 | 3.2109 | 4.0431 | 7.3926 | 7.3933 | 1.0177 | 1.0006 | 1.0006 |
| 260 | 3.0465 | 3.0734 | 3.2109 | 7.0148 | 7.0767 | 7.3933 | 1.0009 | 1.0008 | 1.0006 |
| 261 | 2.4548 | 3.2074 | 3.214 | 5.6525 | 7.3852 | 7.4006 | 1.0035 | 1.0006 | 1.0006 |
| 262 | 2.601 | 3.2041 | 3.2172 | 5.989 | 7.3778 | 7.4079 | 1.0025 | 1.0006 | 1.0006 |
| 263 | 2.7482 | 3.1906 | 3.2172 | 6.3279 | 7.3467 | 7.4079 | 1.0018 | 1.0006 | 1.0006 |
| 264 | 2.9708 | 3.1361 | 3.2193 | 6.8405 | 7.2211 | 7.4128 | 1.0011 | 1.0007 | 1.0006 |
| 265 | 3.0569 | 3.086 | 3.2225 | 7.0388 | 7.1058 | 7.42 | 1.0009 | 1.0008 | 1.0006 |
| 266 | 2.8573 | 3.1816 | 3.2256 | 6.5793 | 7.3258 | 7.4271 | 1.0014 | 1.0007 | 1.0006 |
| 267 | 2.2148 | 3.2245 | 3.2266 | 5.0999 | 7.4248 | 7.4295 | 1.0061 | 1.0006 | 1.0006 |
| 268 | 2.7973 | 3.1942 | 3.2266 | 6.4409 | 7.355 | 7.4295 | 1.0016 | 1.0006 | 1.0006 |
| 269 | 3.0191 | 3.1245 | 3.2287 | 6.9518 | 7.1944 | 7.4343 | 1.001 | 1.0008 | 1.0006 |
| 270 | 2.5159 | 3.2214 | 3.2297 | 5.793 | 7.4176 | 7.4366 | 1.0031 | 1.0006 | 1.0006 |
| 271 | 2.8698 | 3.1875 | 3.2327 | 6.608 | 7.3395 | 7.4437 | 1.0014 | 1.0006 | 1.0006 |
| 272 | 2.692 | 3.2162 | 3.2348 | 6.1985 | 7.4055 | 7.4483 | 1.002 | 1.0006 | 1.0006 |
| 273 | 3.0382 | 3.1222 | 3.2348 | 6.9958 | 7.1892 | 7.4483 | 1.0009 | 1.0008 | 1.0006 |
| 274 | 2.9445 | 3.17 | 3.2358 | 6.7799 | 7.2991 | 7.4507 | 1.0011 | 1.0007 | 1.0006 |
| 275 | 3.0626 | 3.1113 | 3.2388 | 7.0519 | 7.1639 | 7.4576 | 1.0009 | 1.0008 | 1.0006 |
| 276 | 1.7709 | 3.2405 | 3.2408 | 4.0775 | 7.4616 | 7.4622 | 1.0171 | 1.0006 | 1.0006 |
| 277 | 2.248 | 3.2395 | 3.2418 | 5.1761 | 7.4593 | 7.4645 | 1.0057 | 1.0006 | 1.0006 |
| 278 | 2.8169 | 3.2087 | 3.2418 | 6.4862 | 7.3883 | 7.4645 | 1.0015 | 1.0006 | 1.0006 |
| 279 | 2.4698 | 3.2375 | 3.2438 | 5.687 | 7.4547 | 7.4691 | 1.0034 | 1.0006 | 1.0006 |
| 280 | 1.9243 | 3.2463 | 3.2467 | 4.4308 | 7.4748 | 7.4759 | 1.012 | 1.0006 | 1.0006 |
| 281 | 2.616 | 3.2345 | 3.2467 | 6.0234 | 7.4478 | 7.4759 | 1.0024 | 1.0006 | 1.0006 |
| 282 | 2.9863 | 3.1703 | 3.2477 | 6.8763 | 7.2998 | 7.4782 | 1.001 | 1.0007 | 1.0006 |
| 283 | 3.017 | 3.1556 | 3.2477 | 6.947 | 7.2661 | 7.4782 | 1.001 | 1.0007 | 1.0006 |
| 284 | 3.0962 | 3.1021 | 3.2497 | 7.1293 | 7.1428 | 7.4827 | 1.0008 | 1.0008 | 1.0006 |
| 285 | 2.7251 | 3.2304 | 3.2507 | 6.2748 | 7.4384 | 7.4849 | 1.0019 | 1.0006 | 1.0006 |
| 286 | 2.9138 | 3.1989 | 3.2507 | 6.7093 | 7.3658 | 7.4849 | 1.0012 | 1.0006 | 1.0006 |
| 287 | 2.6232 | 3.2403 | 3.2526 | 6.0403 | 7.4611 | 7.4894 | 1.0024 | 1.0006 | 1.0006 |
| 288 | 2.8122 | 3.2253 | 3.2555 | 6.4754 | 7.4265 | 7.4961 | 1.0015 | 1.0006 | 1.0006 |
| 289 | 2.8848 | 3.2191 | 3.2613 | 6.6425 | 7.4122 | 7.5093 | 1.0013 | 1.0006 | 1.0005 |
| 290 | 2.993 | 3.1867 | 3.2613 | 6.8916 | 7.3376 | 7.5093 | 1.001 | 1.0007 | 1.0005 |
| 291 | 2.2355 | 3.266 | 3.2679 | 5.1475 | 7.5202 | 7.5246 | 1.0058 | 1.0005 | 1.0005 |
| 292 | 2.9469 | 3.2117 | 3.2679 | 6.7856 | 7.3951 | 7.5246 | 1.0011 | 1.0006 | 1.0005 |
| 293 | 1.7853 | 3.2695 | 3.2697 | 4.1109 | 7.5283 | 7.5289 | 1.0165 | 1.0005 | 1.0005 |
| 294 | 2.2625 | 3.2686 | 3.2707 | 5.2095 | 7.5262 | 7.531 | 1.0055 | 1.0005 | 1.0005 |
| 295 | 2.5366 | 3.2632 | 3.2707 | 5.8406 | 7.5137 | 7.531 | 1.0029 | 1.0005 | 1.0005 |
| 296 | 2.4843 | 3.2667 | 3.2725 | 5.7203 | 7.5219 | 7.5353 | 1.0033 | 1.0005 | 1.0005 |
| 297 | 3.0599 | 3.1717 | 3.2735 | 7.0458 | 7.3032 | 7.5374 | 1.0009 | 1.0007 | 1.0005 |
| 298 | 2.6304 | 3.2639 | 3.2753 | 6.0568 | 7.5153 | 7.5417 | 1.0023 | 1.0005 | 1.0005 |
| 299 | 2.7126 | 3.2584 | 3.2753 | 6.2461 | 7.5027 | 7.5417 | 1.0019 | 1.0006 | 1.0005 |
| 300 | 2.9657 | 3.2156 | 3.2753 | 6.8287 | 7.4043 | 7.5417 | 1.0011 | 1.0006 | 1.0005 |
| 301 | 3.0013 | 3.203 | 3.2753 | 6.9108 | 7.3753 | 7.5417 | 1.001 | 1.0006 | 1.0005 |
| 302 | 3.1176 | 3.1335 | 3.2762 | 7.1785 | 7.2152 | 7.5438 | 1.0008 | 1.0007 | 1.0005 |
| 303 | 2.7396 | 3.2601 | 3.279 | 6.3081 | 7.5066 | 7.5501 | 1.0018 | 1.0005 | 1.0005 |
| 304 | 2.8376 | 3.2516 | 3.2817 | 6.5338 | 7.4872 | 7.5564 | 1.0015 | 1.0006 | 1.0005 |
| 305 | 2.8267 | 3.2553 | 3.2835 | 6.5088 | 7.4955 | 7.5606 | 1.0015 | 1.0006 | 1.0005 |
| 306 | 3.0496 | 3.1931 | 3.2835 | 7.022 | 7.3524 | 7.5606 | 1.0009 | 1.0006 | 1.0005 |
| 307 | 3.0382 | 3.2028 | 3.2862 | 6.9958 | 7.3746 | 7.5668 | 1.0009 | 1.0006 | 1.0005 |
| 308 | 1.9445 | 3.2867 | 3.2871 | 4.4773 | 7.5679 | 7.5689 | 1.0114 | 1.0005 | 1.0005 |
| 309 | 3.1179 | 3.1538 | 3.2871 | 7.1793 | 7.2619 | 7.5689 | 1.0008 | 1.0007 | 1.0005 |
| 310 | 2.4216 | 3.2849 | 3.2889 | 5.5759 | 7.5637 | 7.573 | 1.0038 | 1.0005 | 1.0005 |
| 311 | 2.8993 | 3.2494 | 3.2889 | 6.6758 | 7.4821 | 7.573 | 1.0013 | 1.0006 | 1.0005 |
| 312 | 2.9345 | 3.2428 | 3.2898 | 6.7569 | 7.4668 | 7.5751 | 1.0012 | 1.0006 | 1.0005 |
| 313 | 2.6435 | 3.2813 | 3.2925 | 6.0868 | 7.5554 | 7.5812 | 1.0023 | 1.0005 | 1.0005 |
| 314 | 3.0931 | 3.1818 | 3.2925 | 7.1221 | 7.3265 | 7.5812 | 1.0008 | 1.0007 | 1.0005 |
| 315 | 2.9614 | 3.2425 | 3.2951 | 6.8189 | 7.4662 | 7.5873 | 1.0011 | 1.0006 | 1.0005 |
| 316 | 1.7993 | 3.2975 | 3.2978 | 4.1431 | 7.5929 | 7.5934 | 1.016 | 1.0005 | 1.0005 |
| 317 | 2.7896 | 3.2758 | 3.2978 | 6.4232 | 7.5427 | 7.5934 | 1.0016 | 1.0005 | 1.0005 |
| 318 | 3.0137 | 3.2317 | 3.2995 | 6.9393 | 7.4413 | 7.5974 | 1.001 | 1.0006 | 1.0005 |
| 319 | 2.4983 | 3.2949 | 3.3004 | 5.7526 | 7.5868 | 7.5994 | 1.0032 | 1.0005 | 1.0005 |
| 320 | 3.0158 | 3.2345 | 3.3021 | 6.9441 | 7.4478 | 7.6034 | 1.001 | 1.0006 | 1.0005 |
| 321 | 3.1326 | 3.1691 | 3.3021 | 7.213 | 7.2971 | 7.6034 | 1.0007 | 1.0007 | 1.0005 |
| 322 | 2.8987 | 3.2683 | 3.3047 | 6.6746 | 7.5256 | 7.6094 | 1.0013 | 1.0005 | 1.0005 |
| 323 | 2.2553 | 3.3056 | 3.3073 | 5.193 | 7.6113 | 7.6153 | 1.0056 | 1.0005 | 1.0005 |
| 324 | 2.5563 | 3.303 | 3.3098 | 5.8861 | 7.6054 | 7.6212 | 1.0028 | 1.0005 | 1.0005 |
| 325 | 3.0641 | 3.2253 | 3.3098 | 7.0553 | 7.4265 | 7.6212 | 1.0009 | 1.0006 | 1.0005 |
| 326 | 2.8407 | 3.2842 | 3.3107 | 6.541 | 7.5622 | 7.6232 | 1.0014 | 1.0005 | 1.0005 |
| 327 | 3.0806 | 3.2183 | 3.3107 | 7.0934 | 7.4103 | 7.6232 | 1.0008 | 1.0006 | 1.0005 |
| 328 | 3.1547 | 3.1688 | 3.3124 | 7.264 | 7.2964 | 7.6271 | 1.0007 | 1.0007 | 1.0005 |
| 329 | 2.9133 | 3.2788 | 3.3158 | 6.7081 | 7.5496 | 7.6348 | 1.0012 | 1.0005 | 1.0005 |
| 330 | 3.1075 | 3.2148 | 3.3183 | 7.1554 | 7.4025 | 7.6406 | 1.0008 | 1.0006 | 1.0005 |
| 331 | 2.8573 | 3.2925 | 3.3199 | 6.5793 | 7.5812 | 7.6444 | 1.0014 | 1.0005 | 1.0005 |
