# Supplementary material for: Datasets on the statistical and algebraic properties of primitive Pythagorean triples
Source: Data Brief. 2017 Sep 1;14:686–94. doi: 10.1016/j.dib.2017.08.021 (PMC5596336; doi:10.1016/j.dib.2017.08.021)
Supplement: Supplementary file 1 — Transparency document [file mmc2.zip › Supplementary Data 9.docx]

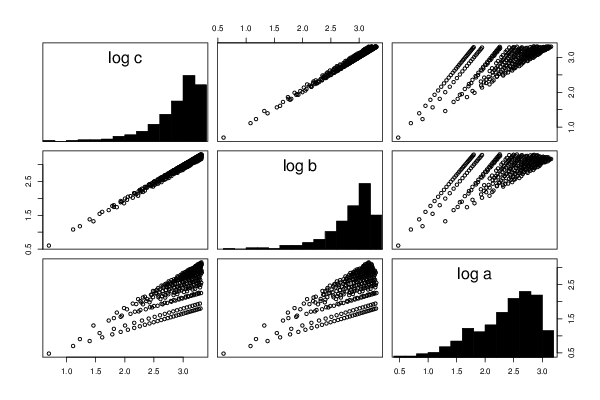


**Figure 17:** Summary of scatter plots of log a, log b and log c


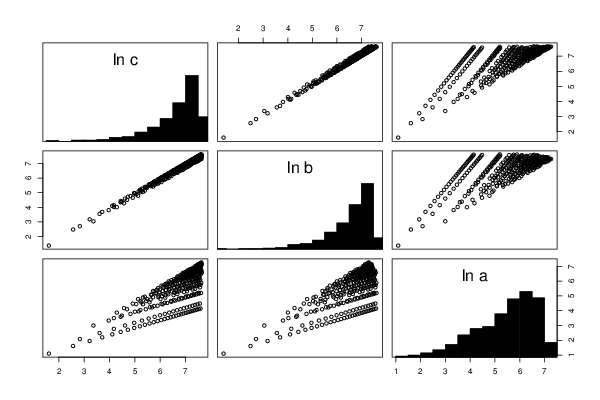


**Figure 18:** Summary of scatter plots of ln a, ln b and ln c


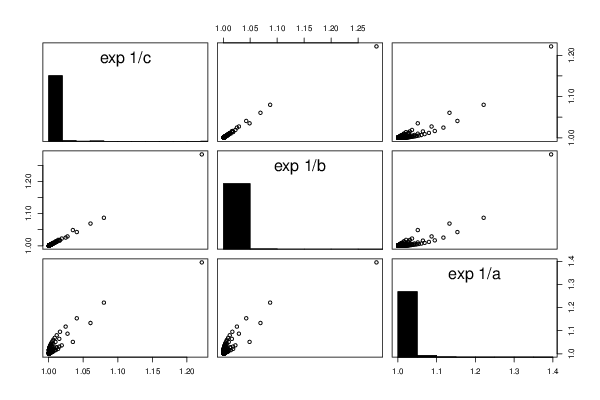


**Figure 19:** Summary of scatter plots of exp 1/a, exp 1/ b and exp 1/ c
