# Supplementary material for: Datasets on the statistical and algebraic properties of primitive Pythagorean triples
Source: Data Brief. 2017 Sep 1;14:686–94. doi: 10.1016/j.dib.2017.08.021 (PMC5596336; doi:10.1016/j.dib.2017.08.021)
Supplement: Supplementary file 1 — Transparency document [file mmc2.zip › Supplementary Data 10.docx]

**Supplementary Data 10:** The tabulations of the digital sum and iterative digits sum of a, b and c

|  | digital sum a | digital sum b | digital sum c | Iterative digits sum a | Iterative digits sum b | Iterative digits sum c |
| --- | --- | --- | --- | --- | --- | --- |
| 1 | 3 | 4 | 5 | 3 | 4 | 5 |
| 2 | 5 | 3 | 4 | 5 | 3 | 4 |
| 3 | 8 | 6 | 8 | 8 | 6 | 8 |
| 4 | 7 | 6 | 7 | 7 | 6 | 7 |
| 5 | 2 | 3 | 11 | 2 | 3 | 2 |
| 6 | 3 | 8 | 10 | 3 | 8 | 1 |
| 7 | 9 | 4 | 5 | 9 | 4 | 5 |
| 8 | 10 | 9 | 8 | 1 | 9 | 8 |
| 9 | 2 | 6 | 7 | 2 | 6 | 7 |
| 10 | 7 | 9 | 11 | 7 | 9 | 2 |
| 11 | 6 | 11 | 11 | 6 | 2 | 2 |
| 12 | 12 | 10 | 10 | 3 | 1 | 1 |
| 13 | 4 | 12 | 13 | 4 | 3 | 4 |
| 14 | 9 | 14 | 13 | 9 | 5 | 4 |
| 15 | 12 | 8 | 17 | 3 | 8 | 8 |
| 16 | 11 | 9 | 16 | 2 | 9 | 7 |
| 17 | 2 | 18 | 2 | 2 | 9 | 2 |
| 18 | 6 | 10 | 10 | 6 | 1 | 1 |
| 19 | 6 | 4 | 5 | 6 | 4 | 5 |
| 20 | 8 | 9 | 8 | 8 | 9 | 8 |
| 21 | 16 | 6 | 11 | 7 | 6 | 2 |
| 22 | 8 | 9 | 10 | 8 | 9 | 1 |
| 23 | 6 | 8 | 10 | 6 | 8 | 1 |
| 24 | 6 | 5 | 14 | 6 | 5 | 5 |
| 25 | 13 | 6 | 13 | 4 | 6 | 4 |
| 26 | 11 | 3 | 16 | 2 | 3 | 7 |
| 27 | 7 | 12 | 11 | 7 | 3 | 2 |
| 28 | 10 | 9 | 10 | 1 | 9 | 1 |
| 29 | 12 | 14 | 14 | 3 | 5 | 5 |
| 30 | 5 | 9 | 14 | 5 | 9 | 5 |
| 31 | 14 | 15 | 13 | 5 | 6 | 4 |
| 32 | 10 | 15 | 17 | 1 | 6 | 8 |
| 33 | 12 | 16 | 7 | 3 | 7 | 7 |
| 34 | 7 | 12 | 7 | 7 | 3 | 7 |
| 35 | 3 | 4 | 5 | 3 | 4 | 5 |
| 36 | 5 | 9 | 5 | 5 | 9 | 5 |
| 37 | 6 | 5 | 13 | 6 | 5 | 4 |
| 38 | 6 | 10 | 8 | 6 | 1 | 8 |
| 39 | 3 | 11 | 7 | 3 | 2 | 7 |
| 40 | 5 | 12 | 14 | 5 | 3 | 5 |
| 41 | 5 | 12 | 13 | 5 | 3 | 4 |
| 42 | 15 | 13 | 13 | 6 | 4 | 4 |
| 43 | 15 | 8 | 17 | 6 | 8 | 8 |
| 44 | 7 | 9 | 16 | 7 | 9 | 7 |
| 45 | 7 | 6 | 11 | 7 | 6 | 2 |
| 46 | 8 | 6 | 19 | 8 | 6 | 1 |
| 47 | 14 | 15 | 14 | 5 | 6 | 5 |
| 48 | 10 | 12 | 8 | 1 | 3 | 8 |
| 49 | 9 | 8 | 8 | 9 | 8 | 8 |
| 50 | 7 | 6 | 7 | 7 | 6 | 7 |
| 51 | 12 | 11 | 11 | 3 | 2 | 2 |
| 52 | 9 | 8 | 10 | 9 | 8 | 1 |
| 53 | 6 | 10 | 10 | 6 | 1 | 1 |
| 54 | 13 | 18 | 13 | 4 | 9 | 4 |
| 55 | 9 | 20 | 16 | 9 | 2 | 7 |
| 56 | 9 | 11 | 11 | 9 | 2 | 2 |
| 57 | 9 | 13 | 14 | 9 | 4 | 5 |
| 58 | 13 | 15 | 14 | 4 | 6 | 5 |
| 59 | 9 | 14 | 13 | 9 | 5 | 4 |
| 60 | 9 | 10 | 17 | 9 | 1 | 8 |
| 61 | 8 | 12 | 17 | 8 | 3 | 8 |
| 62 | 18 | 7 | 20 | 9 | 7 | 2 |
| 63 | 12 | 10 | 19 | 3 | 1 | 1 |
| 64 | 4 | 21 | 5 | 4 | 3 | 5 |
| 65 | 3 | 13 | 13 | 3 | 4 | 4 |
| 66 | 11 | 6 | 7 | 2 | 6 | 7 |
| 67 | 15 | 11 | 11 | 6 | 2 | 2 |
| 68 | 18 | 7 | 11 | 9 | 7 | 2 |
| 69 | 10 | 12 | 10 | 1 | 3 | 1 |
| 70 | 12 | 14 | 13 | 3 | 5 | 4 |
| 71 | 5 | 18 | 13 | 5 | 9 | 4 |
| 72 | 10 | 9 | 17 | 1 | 9 | 8 |
| 73 | 15 | 11 | 16 | 6 | 2 | 7 |
| 74 | 9 | 11 | 11 | 9 | 2 | 2 |
| 75 | 4 | 12 | 13 | 4 | 3 | 4 |
| 76 | 13 | 9 | 13 | 4 | 9 | 4 |
| 77 | 8 | 15 | 17 | 8 | 6 | 8 |
| 78 | 12 | 17 | 17 | 3 | 8 | 8 |
| 79 | 6 | 16 | 16 | 6 | 7 | 7 |
| 80 | 11 | 18 | 16 | 2 | 9 | 7 |
| 81 | 10 | 15 | 10 | 1 | 6 | 1 |
| 82 | 12 | 17 | 10 | 3 | 8 | 1 |
| 83 | 4 | 18 | 14 | 4 | 9 | 5 |
| 84 | 18 | 8 | 8 | 9 | 8 | 8 |
| 85 | 11 | 12 | 11 | 2 | 3 | 2 |
| 86 | 11 | 12 | 11 | 2 | 3 | 2 |
| 87 | 8 | 6 | 10 | 8 | 6 | 1 |
| 88 | 6 | 13 | 14 | 6 | 4 | 5 |
| 89 | 13 | 9 | 14 | 4 | 9 | 5 |
| 90 | 12 | 10 | 17 | 3 | 1 | 8 |
| 91 | 15 | 16 | 16 | 6 | 7 | 7 |
| 92 | 18 | 7 | 16 | 9 | 7 | 7 |
| 93 | 6 | 7 | 20 | 6 | 7 | 2 |
| 94 | 12 | 17 | 19 | 3 | 8 | 1 |
| 95 | 17 | 15 | 17 | 8 | 6 | 8 |
| 96 | 6 | 11 | 7 | 6 | 2 | 7 |
| 97 | 8 | 9 | 10 | 8 | 9 | 1 |
| 98 | 6 | 14 | 14 | 6 | 5 | 5 |
| 99 | 12 | 14 | 13 | 3 | 5 | 4 |
| 100 | 1 | 9 | 17 | 1 | 9 | 8 |
| 101 | 15 | 10 | 17 | 6 | 1 | 8 |
| 102 | 2 | 15 | 11 | 2 | 6 | 2 |
| 103 | 9 | 14 | 14 | 9 | 5 | 5 |
| 104 | 3 | 22 | 13 | 3 | 4 | 4 |
| 105 | 16 | 12 | 16 | 7 | 3 | 7 |
| 106 | 7 | 18 | 20 | 7 | 9 | 2 |
| 107 | 10 | 18 | 19 | 1 | 9 | 1 |
| 108 | 12 | 19 | 19 | 3 | 1 | 1 |
| 109 | 3 | 14 | 23 | 3 | 5 | 5 |
| 110 | 4 | 12 | 23 | 4 | 3 | 5 |
| 111 | 14 | 15 | 22 | 5 | 6 | 4 |
| 112 | 14 | 15 | 22 | 5 | 6 | 4 |
| 113 | 8 | 12 | 8 | 8 | 3 | 8 |
| 114 | 16 | 12 | 16 | 7 | 3 | 7 |
| 115 | 9 | 14 | 14 | 9 | 5 | 5 |
| 116 | 13 | 15 | 14 | 4 | 6 | 5 |
| 117 | 9 | 14 | 13 | 9 | 5 | 4 |
| 118 | 9 | 11 | 16 | 9 | 2 | 7 |
| 119 | 11 | 12 | 16 | 2 | 3 | 7 |
| 120 | 18 | 19 | 19 | 9 | 1 | 1 |
| 121 | 12 | 13 | 14 | 3 | 4 | 5 |
| 122 | 13 | 6 | 22 | 4 | 6 | 4 |
| 123 | 15 | 19 | 17 | 6 | 1 | 8 |
| 124 | 11 | 18 | 20 | 2 | 9 | 2 |
| 125 | 12 | 16 | 20 | 3 | 7 | 2 |
| 126 | 15 | 19 | 19 | 6 | 1 | 1 |
| 127 | 9 | 17 | 19 | 9 | 8 | 1 |
| 128 | 15 | 14 | 23 | 6 | 5 | 5 |
| 129 | 10 | 21 | 17 | 1 | 3 | 8 |
| 130 | 15 | 7 | 11 | 6 | 7 | 2 |
| 131 | 9 | 17 | 19 | 9 | 8 | 1 |
| 132 | 5 | 12 | 13 | 5 | 3 | 4 |
| 133 | 8 | 18 | 17 | 8 | 9 | 8 |
| 134 | 6 | 17 | 17 | 6 | 8 | 8 |
| 135 | 7 | 18 | 16 | 7 | 9 | 7 |
| 136 | 7 | 15 | 20 | 7 | 6 | 2 |
| 137 | 17 | 15 | 19 | 8 | 6 | 1 |
| 138 | 9 | 10 | 19 | 9 | 1 | 1 |
| 139 | 15 | 13 | 22 | 6 | 4 | 4 |
| 140 | 18 | 8 | 17 | 9 | 8 | 8 |
| 141 | 6 | 26 | 10 | 6 | 8 | 1 |
| 142 | 10 | 15 | 10 | 1 | 6 | 1 |
| 143 | 14 | 21 | 14 | 5 | 3 | 5 |
| 144 | 13 | 15 | 14 | 4 | 6 | 5 |
| 145 | 7 | 15 | 16 | 7 | 6 | 7 |
| 146 | 11 | 18 | 16 | 2 | 9 | 7 |
| 147 | 12 | 11 | 20 | 3 | 2 | 2 |
| 148 | 8 | 12 | 19 | 8 | 3 | 1 |
| 149 | 13 | 12 | 14 | 4 | 3 | 5 |
| 150 | 4 | 9 | 22 | 4 | 9 | 4 |
| 151 | 6 | 14 | 22 | 6 | 5 | 4 |
| 152 | 12 | 17 | 17 | 3 | 8 | 8 |
| 153 | 7 | 21 | 20 | 7 | 3 | 2 |
| 154 | 18 | 20 | 20 | 9 | 2 | 2 |
| 155 | 14 | 18 | 23 | 5 | 9 | 5 |
| 156 | 14 | 18 | 22 | 5 | 9 | 4 |
| 157 | 21 | 22 | 22 | 3 | 4 | 4 |
| 158 | 12 | 16 | 25 | 3 | 7 | 7 |
| 159 | 19 | 12 | 10 | 1 | 3 | 1 |
| 160 | 9 | 4 | 5 | 9 | 4 | 5 |
| 161 | 12 | 23 | 4 | 3 | 5 | 4 |
| 162 | 10 | 6 | 8 | 1 | 6 | 8 |
| 163 | 19 | 24 | 8 | 1 | 6 | 8 |
| 164 | 12 | 7 | 7 | 3 | 7 | 7 |
| 165 | 9 | 25 | 11 | 9 | 7 | 2 |
| 166 | 15 | 11 | 11 | 6 | 2 | 2 |
| 167 | 5 | 27 | 14 | 5 | 9 | 5 |
| 168 | 8 | 15 | 8 | 8 | 6 | 8 |
| 169 | 11 | 15 | 16 | 2 | 6 | 7 |
| 170 | 16 | 21 | 11 | 7 | 3 | 2 |
| 171 | 18 | 16 | 11 | 9 | 7 | 2 |
| 172 | 6 | 14 | 13 | 6 | 5 | 4 |
| 173 | 18 | 19 | 17 | 9 | 1 | 8 |
| 174 | 11 | 6 | 7 | 2 | 6 | 7 |
| 175 | 12 | 11 | 7 | 3 | 2 | 7 |
| 176 | 18 | 16 | 7 | 9 | 7 | 7 |
| 177 | 15 | 16 | 7 | 6 | 7 | 7 |
| 178 | 6 | 2 | 11 | 6 | 2 | 2 |
| 179 | 10 | 12 | 10 | 1 | 3 | 1 |
| 180 | 14 | 9 | 13 | 5 | 9 | 4 |
| 181 | 9 | 11 | 11 | 9 | 2 | 2 |
| 182 | 11 | 12 | 11 | 2 | 3 | 2 |
| 183 | 15 | 8 | 10 | 6 | 8 | 1 |
| 184 | 14 | 12 | 14 | 5 | 3 | 5 |
| 185 | 18 | 22 | 14 | 9 | 4 | 5 |
| 186 | 6 | 13 | 13 | 6 | 4 | 4 |
| 187 | 13 | 9 | 13 | 4 | 9 | 4 |
| 188 | 7 | 6 | 11 | 7 | 6 | 2 |
| 189 | 8 | 3 | 19 | 8 | 3 | 1 |
| 190 | 12 | 26 | 19 | 3 | 8 | 1 |
| 191 | 13 | 18 | 14 | 4 | 9 | 5 |
| 192 | 13 | 3 | 4 | 4 | 3 | 4 |
| 193 | 12 | 17 | 8 | 3 | 8 | 8 |
| 194 | 17 | 9 | 8 | 8 | 9 | 8 |
| 195 | 11 | 18 | 7 | 2 | 9 | 7 |
| 196 | 12 | 20 | 11 | 3 | 2 | 2 |
| 197 | 5 | 6 | 14 | 5 | 6 | 5 |
| 198 | 9 | 13 | 13 | 9 | 4 | 4 |
| 199 | 10 | 12 | 8 | 1 | 3 | 8 |
| 200 | 9 | 8 | 8 | 9 | 8 | 8 |
| 201 | 25 | 15 | 16 | 7 | 6 | 7 |
| 202 | 6 | 19 | 10 | 6 | 1 | 1 |
| 203 | 17 | 6 | 10 | 8 | 6 | 1 |
| 204 | 19 | 9 | 17 | 1 | 9 | 8 |
| 205 | 16 | 9 | 16 | 7 | 9 | 7 |
| 206 | 20 | 15 | 16 | 2 | 6 | 7 |
| 207 | 11 | 9 | 20 | 2 | 9 | 2 |
| 208 | 9 | 17 | 19 | 9 | 8 | 1 |
| 209 | 6 | 4 | 5 | 6 | 4 | 5 |
| 210 | 12 | 19 | 8 | 3 | 1 | 8 |
| 211 | 15 | 17 | 8 | 6 | 8 | 8 |
| 212 | 9 | 11 | 7 | 9 | 2 | 7 |
| 213 | 15 | 16 | 11 | 6 | 7 | 2 |
| 214 | 20 | 24 | 11 | 2 | 6 | 2 |
| 215 | 9 | 14 | 13 | 9 | 5 | 4 |
| 216 | 14 | 12 | 13 | 5 | 3 | 4 |
| 217 | 12 | 7 | 11 | 3 | 7 | 2 |
| 218 | 12 | 10 | 19 | 3 | 1 | 1 |
| 219 | 13 | 15 | 14 | 4 | 6 | 5 |
| 220 | 13 | 3 | 13 | 4 | 3 | 4 |
| 221 | 17 | 12 | 17 | 8 | 3 | 8 |
| 222 | 15 | 10 | 17 | 6 | 1 | 8 |
| 223 | 8 | 9 | 10 | 8 | 9 | 1 |
| 224 | 12 | 10 | 10 | 3 | 1 | 1 |
| 225 | 15 | 5 | 14 | 6 | 5 | 5 |
| 226 | 13 | 15 | 13 | 4 | 6 | 4 |
| 227 | 18 | 14 | 13 | 9 | 5 | 4 |
| 228 | 11 | 12 | 16 | 2 | 3 | 7 |
| 229 | 16 | 9 | 11 | 7 | 9 | 2 |
| 230 | 13 | 12 | 14 | 4 | 3 | 5 |
| 231 | 18 | 14 | 14 | 9 | 5 | 5 |
| 232 | 12 | 13 | 13 | 3 | 4 | 4 |
| 233 | 16 | 12 | 16 | 7 | 3 | 7 |
| 234 | 18 | 11 | 16 | 9 | 2 | 7 |
| 235 | 11 | 15 | 20 | 2 | 6 | 2 |
| 236 | 11 | 18 | 20 | 2 | 9 | 2 |
| 237 | 24 | 4 | 14 | 6 | 4 | 5 |
| 238 | 23 | 6 | 22 | 5 | 6 | 4 |
| 239 | 10 | 18 | 17 | 1 | 9 | 8 |
| 240 | 10 | 9 | 10 | 1 | 9 | 1 |
| 241 | 24 | 10 | 10 | 6 | 1 | 1 |
| 242 | 12 | 14 | 14 | 3 | 5 | 5 |
| 243 | 21 | 14 | 14 | 3 | 5 | 5 |
| 244 | 12 | 14 | 13 | 3 | 5 | 4 |
| 245 | 18 | 13 | 13 | 9 | 4 | 4 |
| 246 | 6 | 11 | 16 | 6 | 2 | 7 |
| 247 | 16 | 21 | 16 | 7 | 3 | 7 |
| 248 | 10 | 9 | 19 | 1 | 9 | 1 |
| 249 | 18 | 14 | 14 | 9 | 5 | 5 |
| 250 | 17 | 9 | 17 | 8 | 9 | 8 |
| 251 | 10 | 12 | 17 | 1 | 3 | 8 |
| 252 | 12 | 17 | 19 | 3 | 8 | 1 |
| 253 | 8 | 9 | 19 | 8 | 9 | 1 |
| 254 | 13 | 15 | 22 | 4 | 6 | 4 |
| 255 | 8 | 24 | 8 | 8 | 6 | 8 |
| 256 | 6 | 16 | 16 | 6 | 7 | 7 |
| 257 | 25 | 15 | 11 | 7 | 6 | 2 |
| 258 | 15 | 8 | 10 | 6 | 8 | 1 |
| 259 | 12 | 13 | 14 | 3 | 4 | 5 |
| 260 | 6 | 14 | 14 | 6 | 5 | 5 |
| 261 | 15 | 10 | 17 | 6 | 1 | 8 |
| 262 | 21 | 7 | 20 | 3 | 7 | 2 |
| 263 | 11 | 12 | 20 | 2 | 3 | 2 |
| 264 | 17 | 18 | 19 | 8 | 9 | 1 |
| 265 | 6 | 13 | 22 | 6 | 4 | 4 |
| 266 | 9 | 16 | 16 | 9 | 7 | 7 |
| 267 | 11 | 21 | 20 | 2 | 3 | 2 |
| 268 | 15 | 16 | 20 | 6 | 7 | 2 |
| 269 | 10 | 9 | 19 | 1 | 9 | 1 |
| 270 | 13 | 18 | 23 | 4 | 9 | 5 |
| 271 | 12 | 10 | 17 | 3 | 1 | 8 |
| 272 | 15 | 16 | 16 | 6 | 7 | 7 |
| 273 | 12 | 11 | 16 | 3 | 2 | 7 |
| 274 | 16 | 21 | 11 | 7 | 3 | 2 |
| 275 | 12 | 14 | 14 | 3 | 5 | 5 |
| 276 | 14 | 12 | 13 | 5 | 3 | 4 |
| 277 | 15 | 17 | 17 | 6 | 8 | 8 |
| 278 | 17 | 15 | 17 | 8 | 6 | 8 |
| 279 | 16 | 18 | 16 | 7 | 9 | 7 |
| 280 | 12 | 17 | 19 | 3 | 8 | 1 |
| 281 | 8 | 15 | 19 | 8 | 6 | 1 |
| 282 | 24 | 13 | 23 | 6 | 4 | 5 |
| 283 | 5 | 9 | 23 | 5 | 9 | 5 |
| 284 | 15 | 14 | 22 | 6 | 5 | 4 |
| 285 | 9 | 8 | 17 | 9 | 8 | 8 |
| 286 | 10 | 15 | 17 | 1 | 6 | 8 |
| 287 | 6 | 20 | 25 | 6 | 2 | 7 |
| 288 | 19 | 15 | 10 | 1 | 6 | 1 |
| 289 | 20 | 18 | 16 | 2 | 9 | 7 |
| 290 | 21 | 16 | 16 | 3 | 7 | 7 |
| 291 | 10 | 18 | 17 | 1 | 9 | 8 |
| 292 | 21 | 17 | 17 | 3 | 8 | 8 |
| 293 | 7 | 15 | 16 | 7 | 6 | 7 |
| 294 | 12 | 20 | 20 | 3 | 2 | 2 |
| 295 | 11 | 15 | 20 | 2 | 6 | 2 |
| 296 | 8 | 21 | 19 | 8 | 3 | 1 |
| 297 | 14 | 18 | 23 | 5 | 9 | 5 |
| 298 | 13 | 18 | 22 | 4 | 9 | 4 |
| 299 | 12 | 13 | 22 | 3 | 4 | 4 |
| 300 | 15 | 14 | 22 | 6 | 5 | 4 |
| 301 | 4 | 21 | 22 | 4 | 3 | 4 |
| 302 | 6 | 10 | 26 | 6 | 1 | 8 |
| 303 | 18 | 11 | 11 | 9 | 2 | 2 |
| 304 | 22 | 21 | 14 | 4 | 3 | 5 |
| 305 | 14 | 9 | 13 | 5 | 9 | 4 |
| 306 | 5 | 12 | 13 | 5 | 3 | 4 |
| 307 | 12 | 20 | 16 | 3 | 2 | 7 |
| 308 | 16 | 18 | 20 | 7 | 9 | 2 |
| 309 | 7 | 12 | 20 | 7 | 3 | 2 |
| 310 | 12 | 19 | 19 | 3 | 1 | 1 |
| 311 | 19 | 21 | 19 | 1 | 3 | 1 |
| 312 | 14 | 21 | 23 | 5 | 3 | 5 |
| 313 | 8 | 12 | 17 | 8 | 3 | 8 |
| 314 | 15 | 8 | 17 | 6 | 8 | 8 |
| 315 | 15 | 20 | 20 | 6 | 2 | 2 |
| 316 | 9 | 22 | 23 | 9 | 4 | 5 |
| 317 | 13 | 24 | 23 | 4 | 6 | 5 |
| 318 | 6 | 13 | 22 | 6 | 4 | 4 |
| 319 | 9 | 19 | 26 | 9 | 1 | 8 |
| 320 | 11 | 15 | 7 | 2 | 6 | 7 |
| 321 | 16 | 18 | 7 | 7 | 9 | 7 |
| 322 | 18 | 19 | 10 | 9 | 1 | 1 |
| 323 | 9 | 5 | 13 | 9 | 5 | 4 |
| 324 | 9 | 11 | 7 | 9 | 2 | 7 |
| 325 | 16 | 15 | 7 | 7 | 6 | 7 |
| 326 | 18 | 16 | 11 | 9 | 7 | 2 |
| 327 | 7 | 15 | 11 | 7 | 6 | 2 |
| 328 | 15 | 17 | 10 | 6 | 8 | 1 |
| 329 | 18 | 10 | 17 | 9 | 1 | 8 |
| 330 | 12 | 11 | 11 | 3 | 2 | 2 |
| 331 | 9 | 17 | 19 | 9 | 8 | 1 |
