# Supplementary material for: Datasets on the statistical and algebraic properties of primitive Pythagorean triples
Source: Data Brief. 2017 Sep 1;14:686–94. doi: 10.1016/j.dib.2017.08.021 (PMC5596336; doi:10.1016/j.dib.2017.08.021)
Supplement: Supplementary file 1 — Transparency document [file mmc2.zip › Supplementary Data 1.docx]

**Supplementary Data 1:** Descriptive statistics of the primitive Pythagorean Triples

|  | a | b | c | Mean | b-a | c-b | c-a = range | variance | skewness |
| --- | --- | --- | --- | --- | --- | --- | --- | --- | --- |
| 1 | 3 | 4 | 5 | 4 | 1 | 1 | 2 | 1 | 0 |
| 2 | 5 | 12 | 13 | 10 | 7 | 1 | 8 | 19 | -1.6301 |
| 3 | 8 | 15 | 17 | 13.3333 | 7 | 2 | 9 | 22.3333 | -1.3896 |
| 4 | 7 | 24 | 25 | 18.6667 | 17 | 1 | 18 | 102.3333 | -1.713 |
| 5 | 20 | 21 | 29 | 23.3333 | 1 | 8 | 9 | 24.3333 | 1.6523 |
| 6 | 12 | 35 | 37 | 28 | 23 | 2 | 25 | 193 | -1.6918 |
| 7 | 9 | 40 | 41 | 30 | 31 | 1 | 32 | 331 | -1.7262 |
| 8 | 28 | 45 | 53 | 42 | 17 | 8 | 25 | 163 | -0.999 |
| 9 | 11 | 60 | 61 | 44 | 49 | 1 | 50 | 817 | -1.7297 |
| 10 | 16 | 63 | 65 | 48 | 47 | 2 | 49 | 769 | -1.7219 |
| 11 | 33 | 56 | 65 | 51.3333 | 23 | 9 | 32 | 272.3333 | -1.1708 |
| 12 | 48 | 55 | 73 | 58.6667 | 7 | 18 | 25 | 166.3333 | 1.176 |
| 13 | 13 | 84 | 85 | 60.6667 | 71 | 1 | 72 | 1704.333 | -1.7309 |
| 14 | 36 | 77 | 85 | 66 | 41 | 8 | 49 | 691 | -1.5533 |
| 15 | 39 | 80 | 89 | 69.3333 | 41 | 9 | 50 | 710.3333 | -1.5125 |
| 16 | 65 | 72 | 97 | 78 | 7 | 25 | 32 | 283 | 1.4008 |
| 17 | 20 | 99 | 101 | 73.3333 | 79 | 2 | 81 | 2134.333 | -1.7284 |
| 18 | 60 | 91 | 109 | 86.6667 | 31 | 18 | 49 | 614.3333 | -0.7627 |
| 19 | 15 | 112 | 113 | 80 | 97 | 1 | 98 | 3169 | -1.7314 |
| 20 | 44 | 117 | 125 | 95.3333 | 73 | 8 | 81 | 1992.333 | -1.6697 |
| 21 | 88 | 105 | 137 | 110 | 17 | 32 | 49 | 619 | 0.8678 |
| 22 | 17 | 144 | 145 | 102 | 127 | 1 | 128 | 5419 | -1.7317 |
| 23 | 24 | 143 | 145 | 104 | 119 | 2 | 121 | 4801 | -1.7304 |
| 24 | 51 | 140 | 149 | 113.3333 | 89 | 9 | 98 | 2934.333 | -1.6784 |
| 25 | 85 | 132 | 157 | 124.6667 | 47 | 25 | 72 | 1336.333 | -0.8664 |
| 26 | 119 | 120 | 169 | 136 | 1 | 49 | 50 | 817 | 1.7297 |
| 27 | 52 | 165 | 173 | 130 | 113 | 8 | 121 | 4579 | -1.7049 |
| 28 | 19 | 180 | 181 | 126.6667 | 161 | 1 | 162 | 8694.333 | -1.7318 |
| 29 | 57 | 176 | 185 | 139.3333 | 119 | 9 | 128 | 5104.333 | -1.7012 |
| 30 | 104 | 153 | 185 | 147.3333 | 49 | 32 | 81 | 1664.333 | -0.613 |
| 31 | 95 | 168 | 193 | 152 | 73 | 25 | 98 | 2593 | -1.2743 |
| 32 | 28 | 195 | 197 | 140 | 167 | 2 | 169 | 9409 | -1.7312 |
| 33 | 84 | 187 | 205 | 158.6667 | 103 | 18 | 121 | 4262.333 | -1.5851 |
| 34 | 133 | 156 | 205 | 164.6667 | 23 | 49 | 72 | 1352.333 | 1.0016 |
| 35 | 21 | 220 | 221 | 154 | 199 | 1 | 200 | 13267 | -1.7319 |
| 36 | 140 | 171 | 221 | 177.3333 | 31 | 50 | 81 | 1670.333 | 0.6806 |
| 37 | 60 | 221 | 229 | 170 | 161 | 8 | 169 | 9091 | -1.7183 |
| 38 | 105 | 208 | 233 | 182 | 103 | 25 | 128 | 4603 | -1.4712 |
| 39 | 120 | 209 | 241 | 190 | 89 | 32 | 121 | 3931 | -1.2385 |
| 40 | 32 | 255 | 257 | 181.3333 | 223 | 2 | 225 | 16726.33 | -1.7316 |
| 41 | 23 | 264 | 265 | 184 | 241 | 1 | 242 | 19441 | -1.732 |
| 42 | 96 | 247 | 265 | 202.6667 | 151 | 18 | 169 | 8614.333 | -1.659 |
| 43 | 69 | 260 | 269 | 199.3333 | 191 | 9 | 200 | 12760.33 | -1.7197 |
| 44 | 115 | 252 | 277 | 214.6667 | 137 | 25 | 162 | 7606.333 | -1.5733 |
| 45 | 160 | 231 | 281 | 224 | 71 | 50 | 121 | 3697 | -0.5112 |
| 46 | 161 | 240 | 289 | 230 | 79 | 49 | 128 | 4171 | -0.6801 |
| 47 | 68 | 285 | 293 | 215.3333 | 217 | 8 | 225 | 16296.33 | -1.7244 |
| 48 | 136 | 273 | 305 | 238 | 137 | 32 | 169 | 8059 | -1.4878 |
| 49 | 207 | 224 | 305 | 245.3333 | 17 | 81 | 98 | 2742.333 | 1.529 |
| 50 | 25 | 312 | 313 | 216.6667 | 287 | 1 | 288 | 27552.33 | -1.732 |
| 51 | 75 | 308 | 317 | 233.3333 | 233 | 9 | 242 | 18822.33 | -1.7237 |
| 52 | 36 | 323 | 325 | 228 | 287 | 2 | 289 | 27649 | -1.7318 |
| 53 | 204 | 253 | 325 | 260.6667 | 49 | 72 | 121 | 3704.333 | 0.5579 |
| 54 | 175 | 288 | 337 | 266.6667 | 113 | 49 | 162 | 6902.333 | -1.0793 |
| 55 | 180 | 299 | 349 | 276 | 119 | 50 | 169 | 7537 | -1.1085 |
| 56 | 225 | 272 | 353 | 283.3333 | 47 | 81 | 128 | 4192.333 | 0.7635 |
| 57 | 27 | 364 | 365 | 252 | 337 | 1 | 338 | 37969 | -1.732 |
| 58 | 76 | 357 | 365 | 266 | 281 | 8 | 289 | 27091 | -1.7274 |
| 59 | 252 | 275 | 373 | 300 | 23 | 98 | 121 | 4129 | 1.4858 |
| 60 | 135 | 352 | 377 | 288 | 217 | 25 | 242 | 17713 | -1.6635 |
| 61 | 152 | 345 | 377 | 291.3333 | 193 | 32 | 225 | 14816.33 | -1.5984 |
| 62 | 189 | 340 | 389 | 306 | 151 | 49 | 200 | 10867 | -1.3116 |
| 63 | 228 | 325 | 397 | 316.6667 | 97 | 72 | 169 | 7192.333 | -0.4379 |
| 64 | 40 | 399 | 401 | 280 | 359 | 2 | 361 | 43201 | -1.7319 |
| 65 | 120 | 391 | 409 | 306.6667 | 271 | 18 | 289 | 26214.33 | -1.708 |
| 66 | 29 | 420 | 421 | 290 | 391 | 1 | 392 | 51091 | -1.732 |
| 67 | 87 | 416 | 425 | 309.3333 | 329 | 9 | 338 | 37094.33 | -1.7278 |
| 68 | 297 | 304 | 425 | 342 | 7 | 121 | 128 | 5179 | 1.7136 |
| 69 | 145 | 408 | 433 | 328.6667 | 263 | 25 | 288 | 25456.33 | -1.6843 |
| 70 | 84 | 437 | 445 | 322 | 353 | 8 | 361 | 42499 | -1.7291 |
| 71 | 203 | 396 | 445 | 348 | 193 | 49 | 242 | 16369 | -1.4506 |
| 72 | 280 | 351 | 449 | 360 | 71 | 98 | 169 | 7201 | 0.4719 |
| 73 | 168 | 425 | 457 | 350 | 257 | 32 | 289 | 25099 | -1.6529 |
| 74 | 261 | 380 | 461 | 367.3333 | 119 | 81 | 200 | 10120.33 | -0.5576 |
| 75 | 31 | 480 | 481 | 330.6667 | 449 | 1 | 450 | 67350.33 | -1.732 |
| 76 | 319 | 360 | 481 | 386.6667 | 41 | 121 | 162 | 7094.333 | 1.2819 |
| 77 | 44 | 483 | 485 | 337.3333 | 439 | 2 | 441 | 64534.33 | -1.7319 |
| 78 | 93 | 476 | 485 | 351.3333 | 383 | 9 | 392 | 50072.33 | -1.7289 |
| 79 | 132 | 475 | 493 | 366.6667 | 343 | 18 | 361 | 41382.33 | -1.7168 |
| 80 | 155 | 468 | 493 | 372 | 313 | 25 | 338 | 35473 | -1.6978 |
| 81 | 217 | 456 | 505 | 392.6667 | 239 | 49 | 288 | 23744.33 | -1.5371 |
| 82 | 336 | 377 | 505 | 406 | 41 | 128 | 169 | 7771 | 1.3202 |
| 83 | 220 | 459 | 509 | 396 | 239 | 50 | 289 | 23857 | -1.5301 |
| 84 | 279 | 440 | 521 | 413.3333 | 161 | 81 | 242 | 15174.33 | -0.9285 |
| 85 | 92 | 525 | 533 | 383.3333 | 433 | 8 | 441 | 63672.33 | -1.7301 |
| 86 | 308 | 435 | 533 | 425.3333 | 127 | 98 | 225 | 12726.33 | -0.3828 |
| 87 | 341 | 420 | 541 | 434 | 79 | 121 | 200 | 10147 | 0.6133 |
| 88 | 33 | 544 | 545 | 374 | 511 | 1 | 512 | 87211 | -1.732 |
| 89 | 184 | 513 | 545 | 414 | 329 | 32 | 361 | 39931 | -1.6822 |
| 90 | 165 | 532 | 557 | 418 | 367 | 25 | 392 | 48163 | -1.7068 |
| 91 | 276 | 493 | 565 | 444.6667 | 217 | 72 | 289 | 22632.33 | -1.2965 |
| 92 | 396 | 403 | 565 | 454.6667 | 7 | 162 | 169 | 9142.333 | 1.7216 |
| 93 | 231 | 520 | 569 | 440 | 289 | 49 | 338 | 33361 | -1.5929 |
| 94 | 48 | 575 | 577 | 400 | 527 | 2 | 529 | 92929 | -1.732 |
| 95 | 368 | 465 | 593 | 475.3333 | 97 | 128 | 225 | 12736.33 | 0.4086 |
| 96 | 240 | 551 | 601 | 464 | 311 | 50 | 361 | 38257 | -1.6056 |
| 97 | 35 | 612 | 613 | 420 | 577 | 1 | 578 | 111169 | -1.732 |
| 98 | 105 | 608 | 617 | 443.3333 | 503 | 9 | 512 | 85872.33 | -1.7302 |
| 99 | 336 | 527 | 625 | 496 | 191 | 98 | 289 | 21601 | -0.9069 |
| 100 | 100 | 621 | 629 | 450 | 521 | 8 | 529 | 91891 | -1.7307 |
| 101 | 429 | 460 | 629 | 506 | 31 | 169 | 200 | 11587 | 1.5718 |
| 102 | 200 | 609 | 641 | 483.3333 | 409 | 32 | 441 | 60464.33 | -1.6991 |
| 103 | 315 | 572 | 653 | 513.3333 | 257 | 81 | 338 | 31142.33 | -1.3307 |
| 104 | 300 | 589 | 661 | 516.6667 | 289 | 72 | 361 | 36504.33 | -1.4595 |
| 105 | 385 | 552 | 673 | 536.6667 | 167 | 121 | 288 | 20912.33 | -0.4718 |
| 106 | 52 | 675 | 677 | 468 | 623 | 2 | 625 | 129793 | -1.732 |
| 107 | 37 | 684 | 685 | 468.6667 | 647 | 1 | 648 | 139752.3 | -1.732 |
| 108 | 156 | 667 | 685 | 502.6667 | 511 | 18 | 529 | 90214.33 | -1.7251 |
| 109 | 111 | 680 | 689 | 493.3333 | 569 | 9 | 578 | 109654.3 | -1.7306 |
| 110 | 400 | 561 | 689 | 550 | 161 | 128 | 289 | 20971 | -0.3398 |
| 111 | 185 | 672 | 697 | 518 | 487 | 25 | 512 | 83323 | -1.7174 |
| 112 | 455 | 528 | 697 | 560 | 73 | 169 | 242 | 15409 | 1.083 |
| 113 | 260 | 651 | 701 | 537.3333 | 391 | 50 | 441 | 58310.33 | -1.6489 |
| 114 | 259 | 660 | 709 | 542.6667 | 401 | 49 | 450 | 60950.33 | -1.6556 |
| 115 | 333 | 644 | 725 | 567.3333 | 311 | 81 | 392 | 42824.33 | -1.4383 |
| 116 | 364 | 627 | 725 | 572 | 263 | 98 | 361 | 34849 | -1.2107 |
| 117 | 108 | 725 | 733 | 522 | 617 | 8 | 625 | 128563 | -1.7311 |
| 118 | 216 | 713 | 745 | 558 | 497 | 32 | 529 | 87979 | -1.7094 |
| 119 | 407 | 624 | 745 | 592 | 217 | 121 | 338 | 29329 | -0.8115 |
| 120 | 468 | 595 | 757 | 606.6667 | 127 | 162 | 289 | 20982.33 | 0.3601 |
| 121 | 39 | 760 | 761 | 520 | 721 | 1 | 722 | 173521 | -1.732 |
| 122 | 481 | 600 | 769 | 616.6667 | 119 | 169 | 288 | 20944.33 | 0.5114 |
| 123 | 195 | 748 | 773 | 572 | 553 | 25 | 578 | 106753 | -1.7206 |
| 124 | 56 | 783 | 785 | 541.3333 | 727 | 2 | 729 | 176662.3 | -1.732 |
| 125 | 273 | 736 | 785 | 598 | 463 | 49 | 512 | 79819 | -1.6736 |
| 126 | 168 | 775 | 793 | 578.6667 | 607 | 18 | 625 | 126566.3 | -1.7271 |
| 127 | 432 | 665 | 793 | 630 | 233 | 128 | 361 | 33499 | -0.8291 |
| 128 | 555 | 572 | 797 | 641.3333 | 17 | 225 | 242 | 18246.33 | 1.7012 |
| 129 | 280 | 759 | 809 | 616 | 479 | 50 | 529 | 85297 | -1.6751 |
| 130 | 429 | 700 | 821 | 650 | 271 | 121 | 392 | 40291 | -1.0514 |
| 131 | 540 | 629 | 829 | 666 | 89 | 200 | 289 | 21907 | 1.0546 |
| 132 | 41 | 840 | 841 | 574 | 799 | 1 | 800 | 213067 | -1.732 |
| 133 | 116 | 837 | 845 | 599.3333 | 721 | 8 | 729 | 175224.3 | -1.7313 |
| 134 | 123 | 836 | 845 | 601.3333 | 713 | 9 | 722 | 171622.3 | -1.7311 |
| 135 | 205 | 828 | 853 | 628.6667 | 623 | 25 | 648 | 134776.3 | -1.723 |
| 136 | 232 | 825 | 857 | 638 | 593 | 32 | 625 | 123883 | -1.716 |
| 137 | 287 | 816 | 865 | 656 | 529 | 49 | 578 | 102721 | -1.6866 |
| 138 | 504 | 703 | 865 | 690.6667 | 199 | 162 | 361 | 32694.33 | -0.3055 |
| 139 | 348 | 805 | 877 | 676.6667 | 457 | 72 | 529 | 82312.33 | -1.6101 |
| 140 | 369 | 800 | 881 | 683.3333 | 431 | 81 | 512 | 75744.33 | -1.5648 |
| 141 | 60 | 899 | 901 | 620 | 839 | 2 | 841 | 235201 | -1.732 |
| 142 | 451 | 780 | 901 | 710.6667 | 329 | 121 | 450 | 54230.33 | -1.221 |
| 143 | 464 | 777 | 905 | 715.3333 | 313 | 128 | 441 | 51472.33 | -1.1328 |
| 144 | 616 | 663 | 905 | 728 | 47 | 242 | 289 | 24049 | 1.5548 |
| 145 | 43 | 924 | 925 | 630.6667 | 881 | 1 | 882 | 259014.3 | -1.732 |
| 146 | 533 | 756 | 925 | 738 | 223 | 169 | 392 | 38659 | -0.4085 |
| 147 | 129 | 920 | 929 | 659.3333 | 791 | 9 | 800 | 210960.3 | -1.7313 |
| 148 | 215 | 912 | 937 | 688 | 697 | 25 | 722 | 167953 | -1.7248 |
| 149 | 580 | 741 | 941 | 754 | 161 | 200 | 361 | 32707 | 0.3218 |
| 150 | 301 | 900 | 949 | 716.6667 | 599 | 49 | 648 | 130184.3 | -1.6962 |
| 151 | 420 | 851 | 949 | 740 | 431 | 98 | 529 | 79201 | -1.4988 |
| 152 | 615 | 728 | 953 | 765.3333 | 113 | 225 | 338 | 29606.33 | 0.9304 |
| 153 | 124 | 957 | 965 | 682 | 833 | 8 | 841 | 233539 | -1.7315 |
| 154 | 387 | 884 | 965 | 745.3333 | 497 | 81 | 578 | 97942.33 | -1.6024 |
| 155 | 248 | 945 | 977 | 723.3333 | 697 | 32 | 729 | 169712.3 | -1.7203 |
| 156 | 473 | 864 | 985 | 774 | 391 | 121 | 512 | 71611 | -1.3423 |
| 157 | 696 | 697 | 985 | 792.6667 | 1 | 288 | 289 | 27744.33 | 1.732 |
| 158 | 372 | 925 | 997 | 764.6667 | 553 | 72 | 625 | 116936.3 | -1.6461 |
| 159 | 559 | 840 | 1009 | 802.6667 | 281 | 169 | 450 | 51670.33 | -0.7191 |
| 160 | 45 | 1012 | 1013 | 690 | 967 | 1 | 968 | 312019 | -1.732 |
| 161 | 660 | 779 | 1021 | 820 | 119 | 242 | 361 | 33841 | 0.9531 |
| 162 | 64 | 1023 | 1025 | 704 | 959 | 2 | 961 | 307201 | -1.732 |
| 163 | 496 | 897 | 1025 | 806 | 401 | 128 | 529 | 76171 | -1.3224 |
| 164 | 192 | 1015 | 1033 | 746.6667 | 823 | 18 | 841 | 230822.3 | -1.7293 |
| 165 | 315 | 988 | 1037 | 780 | 673 | 49 | 722 | 162769 | -1.7034 |
| 166 | 645 | 812 | 1037 | 831.3333 | 167 | 225 | 392 | 38696.33 | 0.438 |
| 167 | 320 | 999 | 1049 | 789.3333 | 679 | 50 | 729 | 165830.3 | -1.7027 |
| 168 | 620 | 861 | 1061 | 847.3333 | 241 | 200 | 441 | 48760.33 | -0.2774 |
| 169 | 731 | 780 | 1069 | 860 | 49 | 289 | 338 | 33361 | 1.5929 |
| 170 | 448 | 975 | 1073 | 832 | 527 | 98 | 625 | 112993 | -1.5679 |
| 171 | 495 | 952 | 1073 | 840 | 457 | 121 | 578 | 92929 | -1.4301 |
| 172 | 132 | 1085 | 1093 | 770 | 953 | 8 | 961 | 305299 | -1.7316 |
| 173 | 585 | 928 | 1097 | 870 | 343 | 169 | 512 | 68059 | -0.951 |
| 174 | 47 | 1104 | 1105 | 752 | 1057 | 1 | 1058 | 372769 | -1.732 |
| 175 | 264 | 1073 | 1105 | 814 | 809 | 32 | 841 | 227131 | -1.7233 |
| 176 | 576 | 943 | 1105 | 874.6667 | 367 | 162 | 529 | 73462.33 | -1.0624 |
| 177 | 744 | 817 | 1105 | 888.6667 | 73 | 288 | 361 | 36432.33 | 1.4514 |
| 178 | 141 | 1100 | 1109 | 783.3333 | 959 | 9 | 968 | 309464.3 | -1.7315 |
| 179 | 235 | 1092 | 1117 | 814.6667 | 857 | 25 | 882 | 252166.3 | -1.7272 |
| 180 | 329 | 1080 | 1129 | 846 | 751 | 49 | 800 | 201067 | -1.7088 |
| 181 | 423 | 1064 | 1145 | 877.3333 | 641 | 81 | 722 | 156454.3 | -1.6507 |
| 182 | 704 | 903 | 1145 | 917.3333 | 199 | 242 | 441 | 48774.33 | 0.2908 |
| 183 | 528 | 1025 | 1153 | 902 | 497 | 128 | 625 | 109003 | -1.4438 |
| 184 | 68 | 1155 | 1157 | 793.3333 | 1087 | 2 | 1089 | 394582.3 | -1.732 |
| 185 | 765 | 868 | 1157 | 930 | 103 | 289 | 392 | 41299 | 1.2451 |
| 186 | 204 | 1147 | 1165 | 838.6667 | 943 | 18 | 961 | 302182.3 | -1.73 |
| 187 | 517 | 1044 | 1165 | 908.6667 | 527 | 121 | 648 | 118712.3 | -1.4948 |
| 188 | 340 | 1131 | 1181 | 884 | 791 | 50 | 841 | 222577 | -1.7102 |
| 189 | 611 | 1020 | 1189 | 940 | 409 | 169 | 578 | 88321 | -1.1236 |
| 190 | 660 | 989 | 1189 | 946 | 329 | 200 | 529 | 71347 | -0.7057 |
| 191 | 832 | 855 | 1193 | 960 | 23 | 338 | 361 | 40849 | 1.7069 |
| 192 | 49 | 1200 | 1201 | 816.6667 | 1151 | 1 | 1152 | 441984.3 | -1.732 |
| 193 | 147 | 1196 | 1205 | 849.3333 | 1049 | 9 | 1058 | 369974.3 | -1.7316 |
| 194 | 476 | 1107 | 1205 | 929.3333 | 631 | 98 | 729 | 156534.3 | -1.6133 |
| 195 | 245 | 1188 | 1213 | 882 | 943 | 25 | 968 | 304483 | -1.7281 |
| 196 | 705 | 992 | 1217 | 971.3333 | 287 | 225 | 512 | 65856.33 | -0.36 |
| 197 | 140 | 1221 | 1229 | 863.3333 | 1081 | 8 | 1089 | 392424.3 | -1.7317 |
| 198 | 612 | 1075 | 1237 | 974.6667 | 463 | 162 | 625 | 105206.3 | -1.2588 |
| 199 | 280 | 1209 | 1241 | 910 | 929 | 32 | 961 | 297931 | -1.7254 |
| 200 | 441 | 1160 | 1241 | 947.3333 | 719 | 81 | 800 | 193920.3 | -1.6664 |
| 201 | 799 | 960 | 1249 | 1002.667 | 161 | 289 | 450 | 51990.33 | 0.8126 |
| 202 | 420 | 1189 | 1261 | 956.6667 | 769 | 72 | 841 | 217304.3 | -1.6857 |
| 203 | 539 | 1140 | 1261 | 980 | 601 | 121 | 722 | 149521 | -1.5432 |
| 204 | 748 | 1035 | 1277 | 1020 | 287 | 242 | 529 | 70129 | -0.2541 |
| 205 | 637 | 1116 | 1285 | 1012.667 | 479 | 169 | 648 | 112984.3 | -1.2526 |
| 206 | 893 | 924 | 1285 | 1034 | 31 | 361 | 392 | 47491 | 1.6927 |
| 207 | 560 | 1161 | 1289 | 1003.333 | 601 | 128 | 729 | 151504.3 | -1.5237 |
| 208 | 72 | 1295 | 1297 | 888 | 1223 | 2 | 1225 | 499393 | -1.732 |
| 209 | 51 | 1300 | 1301 | 884 | 1249 | 1 | 1250 | 520417 | -1.732 |
| 210 | 255 | 1288 | 1313 | 952 | 1033 | 25 | 1058 | 364513 | -1.7287 |
| 211 | 735 | 1088 | 1313 | 1045.333 | 353 | 225 | 578 | 84886.33 | -0.6449 |
| 212 | 360 | 1271 | 1321 | 984 | 911 | 50 | 961 | 292657 | -1.7154 |
| 213 | 357 | 1276 | 1325 | 986 | 919 | 49 | 968 | 297331 | -1.7163 |
| 214 | 884 | 987 | 1325 | 1065.333 | 103 | 338 | 441 | 53222.33 | 1.3518 |
| 215 | 504 | 1247 | 1345 | 1032 | 743 | 98 | 841 | 211489 | -1.644 |
| 216 | 833 | 1056 | 1345 | 1078 | 223 | 289 | 512 | 65899 | 0.3828 |
| 217 | 561 | 1240 | 1361 | 1054 | 679 | 121 | 800 | 185947 | -1.5799 |
| 218 | 840 | 1081 | 1369 | 1096.667 | 241 | 288 | 529 | 70144.33 | 0.2653 |
| 219 | 148 | 1365 | 1373 | 962 | 1217 | 8 | 1225 | 496963 | -1.7318 |
| 220 | 931 | 1020 | 1381 | 1110.667 | 89 | 361 | 450 | 56790.33 | 1.4643 |
| 221 | 296 | 1353 | 1385 | 1011.333 | 1057 | 32 | 1089 | 384032.3 | -1.7269 |
| 222 | 663 | 1216 | 1385 | 1088 | 553 | 169 | 722 | 142609 | -1.35 |
| 223 | 53 | 1404 | 1405 | 954 | 1351 | 1 | 1352 | 608851 | -1.732 |
| 224 | 444 | 1333 | 1405 | 1060.667 | 889 | 72 | 961 | 286504.3 | -1.6969 |
| 225 | 159 | 1400 | 1409 | 989.3333 | 1241 | 9 | 1250 | 517110.3 | -1.7317 |
| 226 | 265 | 1392 | 1417 | 1024.667 | 1127 | 25 | 1152 | 432976.3 | -1.7292 |
| 227 | 792 | 1175 | 1417 | 1128 | 383 | 242 | 625 | 99313 | -0.6562 |
| 228 | 371 | 1380 | 1429 | 1060 | 1009 | 49 | 1058 | 356641 | -1.7189 |
| 229 | 592 | 1305 | 1433 | 1110 | 713 | 128 | 841 | 205339 | -1.5779 |
| 230 | 76 | 1443 | 1445 | 988 | 1367 | 2 | 1369 | 623809 | -1.732 |
| 231 | 477 | 1364 | 1445 | 1095.333 | 887 | 81 | 968 | 288392.3 | -1.6878 |
| 232 | 228 | 1435 | 1453 | 1038.667 | 1207 | 18 | 1225 | 492966.3 | -1.7308 |
| 233 | 583 | 1344 | 1465 | 1130.667 | 761 | 121 | 882 | 228614.3 | -1.6081 |
| 234 | 936 | 1127 | 1465 | 1176 | 191 | 338 | 529 | 71761 | 0.7956 |
| 235 | 380 | 1419 | 1469 | 1089.333 | 1039 | 50 | 1089 | 377990.3 | -1.7192 |
| 236 | 740 | 1269 | 1469 | 1159.333 | 529 | 200 | 729 | 141880.3 | -1.1991 |
| 237 | 969 | 1120 | 1481 | 1190 | 151 | 361 | 512 | 69211 | 1.1126 |
| 238 | 689 | 1320 | 1489 | 1166 | 631 | 169 | 800 | 177787 | -1.4243 |
| 239 | 532 | 1395 | 1493 | 1140 | 863 | 98 | 961 | 279649 | -1.6654 |
| 240 | 55 | 1512 | 1513 | 1026.667 | 1457 | 1 | 1458 | 708102.3 | -1.732 |
| 241 | 888 | 1225 | 1513 | 1208.667 | 337 | 288 | 625 | 97856.33 | -0.2343 |
| 242 | 165 | 1508 | 1517 | 1063.333 | 1343 | 9 | 1352 | 605272.3 | -1.7318 |
| 243 | 795 | 1292 | 1517 | 1201.333 | 497 | 225 | 722 | 136486.3 | -1.0379 |
| 244 | 156 | 1517 | 1525 | 1066 | 1361 | 8 | 1369 | 621091 | -1.7319 |
| 245 | 684 | 1363 | 1525 | 1190.667 | 679 | 162 | 841 | 199094.3 | -1.4788 |
| 246 | 312 | 1505 | 1537 | 1118 | 1193 | 32 | 1225 | 487483 | -1.728 |
| 247 | 385 | 1488 | 1537 | 1136.667 | 1103 | 49 | 1152 | 424352.3 | -1.721 |
| 248 | 901 | 1260 | 1549 | 1236.667 | 359 | 289 | 648 | 105384.3 | -0.3218 |
| 249 | 495 | 1472 | 1553 | 1173.333 | 977 | 81 | 1058 | 346742.3 | -1.6953 |
| 250 | 836 | 1323 | 1565 | 1241.333 | 487 | 242 | 729 | 137862.3 | -0.9419 |
| 251 | 1036 | 1173 | 1565 | 1258 | 137 | 392 | 529 | 75379 | 1.2596 |
| 252 | 624 | 1457 | 1585 | 1222 | 833 | 128 | 961 | 272299 | -1.6155 |
| 253 | 1007 | 1224 | 1585 | 1272 | 217 | 361 | 578 | 85249 | 0.7198 |
| 254 | 715 | 1428 | 1597 | 1246.667 | 713 | 169 | 882 | 219142.3 | -1.4816 |
| 255 | 80 | 1599 | 1601 | 1093.333 | 1519 | 2 | 1521 | 770134.3 | -1.732 |
| 256 | 240 | 1591 | 1609 | 1146.667 | 1351 | 18 | 1369 | 616614.3 | -1.731 |
| 257 | 988 | 1275 | 1613 | 1292 | 287 | 338 | 625 | 97873 | 0.2438 |
| 258 | 780 | 1421 | 1621 | 1274 | 641 | 200 | 841 | 193027 | -1.3371 |
| 259 | 57 | 1624 | 1625 | 1102 | 1567 | 1 | 1568 | 819019 | -1.732 |
| 260 | 1113 | 1184 | 1625 | 1307.333 | 71 | 441 | 512 | 76944.33 | 1.6053 |
| 261 | 285 | 1612 | 1637 | 1178 | 1327 | 25 | 1352 | 598243 | -1.73 |
| 262 | 399 | 1600 | 1649 | 1216 | 1201 | 49 | 1250 | 501217 | -1.7227 |
| 263 | 560 | 1551 | 1649 | 1253.333 | 991 | 98 | 1089 | 362934.3 | -1.6806 |
| 264 | 935 | 1368 | 1657 | 1320 | 433 | 289 | 722 | 132049 | -0.584 |
| 265 | 1140 | 1219 | 1669 | 1342.667 | 79 | 450 | 529 | 81430.33 | 1.5839 |
| 266 | 720 | 1519 | 1681 | 1306.667 | 799 | 162 | 961 | 264694.3 | -1.5409 |
| 267 | 164 | 1677 | 1685 | 1175.333 | 1513 | 8 | 1521 | 767112.3 | -1.7319 |
| 268 | 627 | 1564 | 1685 | 1292 | 937 | 121 | 1058 | 335329 | -1.6474 |
| 269 | 1045 | 1332 | 1693 | 1356.667 | 287 | 361 | 648 | 105432.3 | 0.3399 |
| 270 | 328 | 1665 | 1697 | 1230 | 1337 | 32 | 1369 | 610459 | -1.7288 |
| 271 | 741 | 1540 | 1709 | 1330 | 799 | 169 | 968 | 267331 | -1.5262 |
| 272 | 492 | 1645 | 1717 | 1284.667 | 1153 | 72 | 1225 | 472536.3 | -1.7107 |
| 273 | 1092 | 1325 | 1717 | 1378 | 233 | 392 | 625 | 99763 | 0.7338 |
| 274 | 880 | 1479 | 1721 | 1360 | 599 | 242 | 841 | 187441 | -1.1434 |
| 275 | 1155 | 1292 | 1733 | 1393.333 | 137 | 441 | 578 | 91222.33 | 1.3398 |
| 276 | 59 | 1740 | 1741 | 1180 | 1681 | 1 | 1682 | 942481 | -1.732 |
| 277 | 177 | 1736 | 1745 | 1219.333 | 1559 | 9 | 1568 | 814864.3 | -1.7319 |
| 278 | 656 | 1617 | 1745 | 1339.333 | 961 | 128 | 1089 | 354304.3 | -1.6424 |
| 279 | 295 | 1728 | 1753 | 1258.667 | 1433 | 25 | 1458 | 696646.3 | -1.7303 |
| 280 | 84 | 1763 | 1765 | 1204 | 1679 | 2 | 1681 | 940801 | -1.732 |
| 281 | 413 | 1716 | 1765 | 1298 | 1303 | 49 | 1352 | 588019 | -1.7241 |
| 282 | 969 | 1480 | 1769 | 1406 | 511 | 289 | 800 | 164107 | -0.7946 |
| 283 | 1040 | 1431 | 1769 | 1413.333 | 391 | 338 | 729 | 133094.3 | -0.2174 |
| 284 | 1248 | 1265 | 1777 | 1430 | 17 | 512 | 529 | 90379 | 1.7258 |
| 285 | 531 | 1700 | 1781 | 1337.333 | 1169 | 81 | 1250 | 489270.3 | -1.706 |
| 286 | 820 | 1581 | 1781 | 1394 | 761 | 200 | 961 | 257107 | -1.4339 |
| 287 | 420 | 1739 | 1789 | 1316 | 1319 | 50 | 1369 | 602737 | -1.724 |
| 288 | 649 | 1680 | 1801 | 1376.667 | 1031 | 121 | 1152 | 400784.3 | -1.6611 |
| 289 | 767 | 1656 | 1825 | 1416 | 889 | 169 | 1058 | 323041 | -1.5614 |
| 290 | 984 | 1537 | 1825 | 1448.667 | 553 | 288 | 841 | 182672.3 | -0.8903 |
| 291 | 172 | 1845 | 1853 | 1290 | 1673 | 8 | 1681 | 937459 | -1.7319 |
| 292 | 885 | 1628 | 1853 | 1455.333 | 743 | 225 | 968 | 256616.3 | -1.3556 |
| 293 | 61 | 1860 | 1861 | 1260.667 | 1799 | 1 | 1800 | 1079400 | -1.732 |
| 294 | 183 | 1856 | 1865 | 1301.333 | 1673 | 9 | 1682 | 938022.3 | -1.7319 |
| 295 | 344 | 1833 | 1865 | 1347.333 | 1489 | 32 | 1521 | 755264.3 | -1.7294 |
| 296 | 305 | 1848 | 1873 | 1342 | 1543 | 25 | 1568 | 806683 | -1.7305 |
| 297 | 1148 | 1485 | 1877 | 1503.333 | 337 | 392 | 729 | 133112.3 | 0.2256 |
| 298 | 427 | 1836 | 1885 | 1382.667 | 1409 | 49 | 1458 | 685574.3 | -1.7252 |
| 299 | 516 | 1813 | 1885 | 1404.667 | 1297 | 72 | 1369 | 593592.3 | -1.715 |
| 300 | 924 | 1643 | 1885 | 1484 | 719 | 242 | 961 | 249841 | -1.2866 |
| 301 | 1003 | 1596 | 1885 | 1494.667 | 593 | 289 | 882 | 202182.3 | -0.9626 |
| 302 | 1311 | 1360 | 1889 | 1520 | 49 | 529 | 578 | 102721 | 1.6866 |
| 303 | 549 | 1820 | 1901 | 1423.333 | 1271 | 81 | 1352 | 574984.3 | -1.7098 |
| 304 | 688 | 1785 | 1913 | 1462 | 1097 | 128 | 1225 | 453403 | -1.6619 |
| 305 | 671 | 1800 | 1921 | 1464 | 1129 | 121 | 1250 | 475297 | -1.6722 |
| 306 | 1121 | 1560 | 1921 | 1534 | 439 | 361 | 800 | 160507 | -0.2908 |
| 307 | 1092 | 1595 | 1933 | 1540 | 503 | 338 | 841 | 179089 | -0.575 |
| 308 | 88 | 1935 | 1937 | 1320 | 1847 | 2 | 1849 | 1138369 | -1.732 |
| 309 | 1312 | 1425 | 1937 | 1558 | 113 | 512 | 625 | 110923 | 1.5104 |
| 310 | 264 | 1927 | 1945 | 1378.667 | 1663 | 18 | 1681 | 931942.3 | -1.7314 |
| 311 | 793 | 1776 | 1945 | 1504.667 | 983 | 169 | 1152 | 386992.3 | -1.5894 |
| 312 | 860 | 1749 | 1949 | 1519.333 | 889 | 200 | 1089 | 336040.3 | -1.503 |
| 313 | 440 | 1911 | 1961 | 1437.333 | 1471 | 50 | 1521 | 746630.3 | -1.7255 |
| 314 | 1239 | 1520 | 1961 | 1573.333 | 281 | 441 | 722 | 132454.3 | 0.6453 |
| 315 | 915 | 1748 | 1973 | 1545.333 | 833 | 225 | 1058 | 310646.3 | -1.4199 |
| 316 | 63 | 1984 | 1985 | 1344 | 1921 | 1 | 1922 | 1230721 | -1.732 |
| 317 | 616 | 1887 | 1985 | 1496 | 1271 | 98 | 1369 | 583201 | -1.7 |
| 318 | 1032 | 1705 | 1993 | 1576.667 | 673 | 288 | 961 | 243232.3 | -1.0917 |
| 319 | 315 | 1972 | 1997 | 1428 | 1657 | 25 | 1682 | 929233 | -1.7307 |
| 320 | 1037 | 1716 | 2005 | 1586 | 679 | 289 | 968 | 246931 | -1.0967 |
| 321 | 1357 | 1476 | 2005 | 1612.667 | 119 | 529 | 648 | 118984.3 | 1.503 |
| 322 | 792 | 1855 | 2017 | 1554.667 | 1063 | 162 | 1225 | 442806.3 | -1.6173 |
| 323 | 180 | 2021 | 2029 | 1410 | 1841 | 8 | 1849 | 1134691 | -1.7319 |
| 324 | 360 | 2009 | 2041 | 1470 | 1649 | 32 | 1681 | 924331 | -1.7299 |
| 325 | 1159 | 1680 | 2041 | 1626.667 | 521 | 361 | 882 | 196614.3 | -0.5334 |
| 326 | 693 | 1924 | 2045 | 1554 | 1231 | 121 | 1352 | 559651 | -1.6812 |
| 327 | 1204 | 1653 | 2045 | 1634 | 449 | 392 | 841 | 177091 | -0.2028 |
| 328 | 1428 | 1475 | 2053 | 1652 | 47 | 578 | 625 | 121153 | 1.6966 |
| 329 | 819 | 1900 | 2069 | 1596 | 1081 | 169 | 1250 | 459937 | -1.6118 |
| 330 | 1281 | 1640 | 2081 | 1667.333 | 359 | 441 | 800 | 160560.3 | 0.3055 |
| 331 | 720 | 1961 | 2089 | 1590 | 1241 | 128 | 1369 | 571771 | -1.6764 |
