# Supplementary material for: Datasets on the statistical and algebraic properties of primitive Pythagorean triples
Source: Data Brief. 2017 Sep 1;14:686–94. doi: 10.1016/j.dib.2017.08.021 (PMC5596336; doi:10.1016/j.dib.2017.08.021)
Supplement: Supplementary file 1 — Transparency document [file mmc2.zip › Supplementary Data 2.docx]

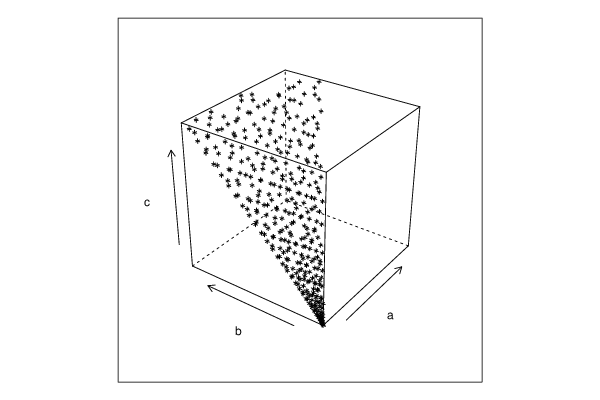
 **Figure 1:** 3-Dimensional scatter plots of a, b and c (1)


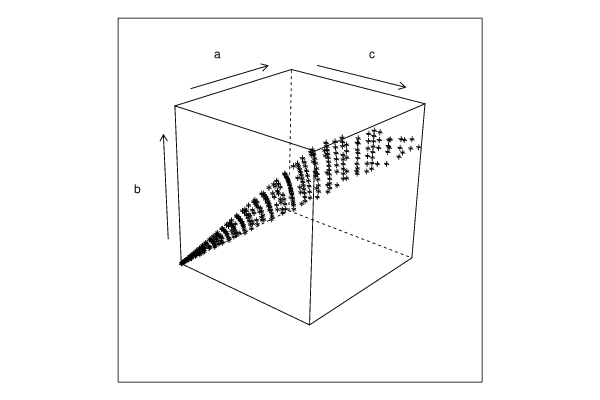


**Figure 2:** 3-Dimensional scatter plots of a, b and c (2)


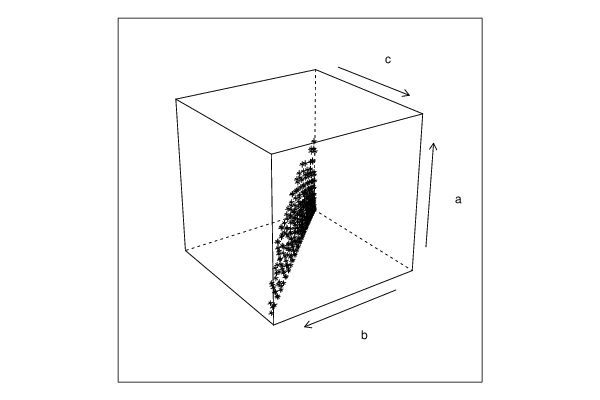
 **Figure 3:** 3-Dimensional scatter plots of a, b and c (3)
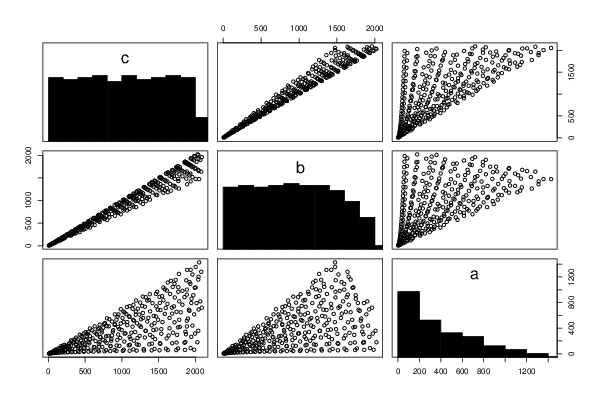


**Figure 4:** Summary of scatter plots of a, b and c.


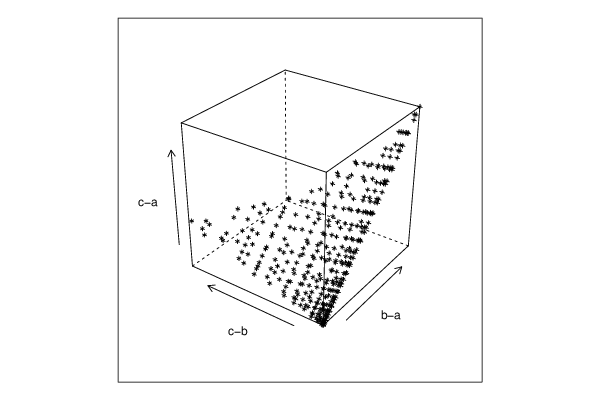
 **Figure 5:** 3-Dimensional scatter plots of b-a, c-b and c-a (1)
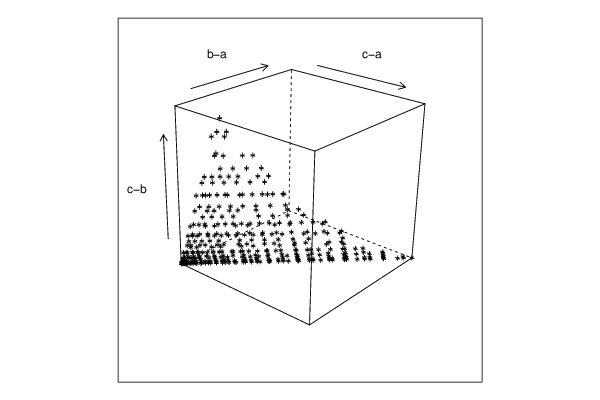


**Figure 6:** 3-Dimensional scatter plots of b-a, c-b and c-a (2)


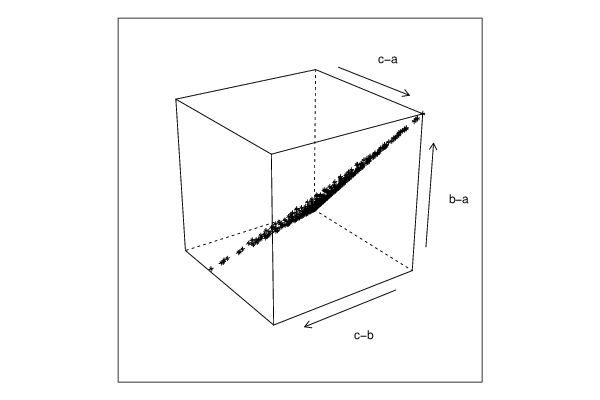
 **Figure 7:** 3-Dimensional scatter plots of b-a, c-b and c-a (3)


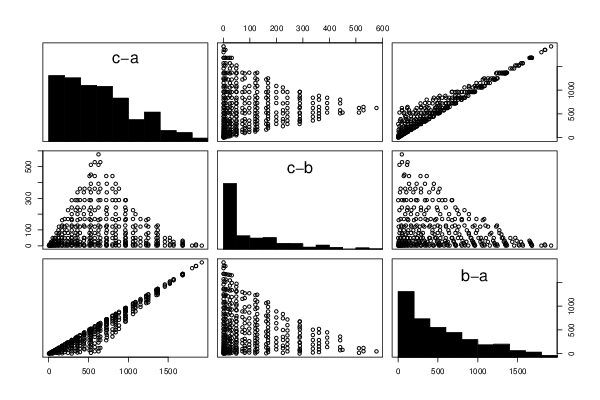


**Figure 8:** Summary of scatter plots of b-a, c-b and c-a.


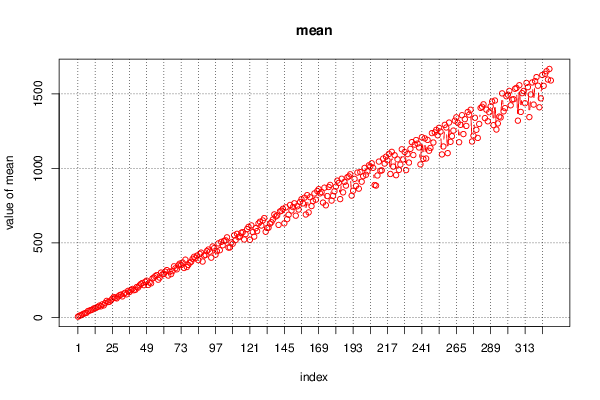


**Figure 9:** The line plot of the mean of the integers a, b, c for the primitive Pythagorean triples
