# Supplementary material for: Datasets on the statistical and algebraic properties of primitive Pythagorean triples
Source: Data Brief. 2017 Sep 1;14:686–94. doi: 10.1016/j.dib.2017.08.021 (PMC5596336; doi:10.1016/j.dib.2017.08.021)
Supplement: Supplementary file 1 — Transparency document [file mmc2.zip › Supplementary Data 3.docx]

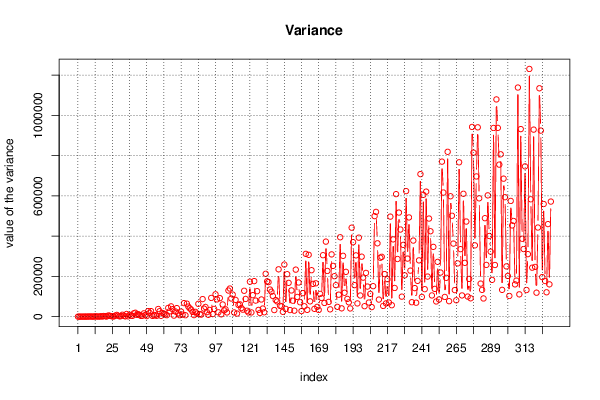
**Figure 10:** The line plot of the variance of the integers a, b, c for the Pythagorean triples
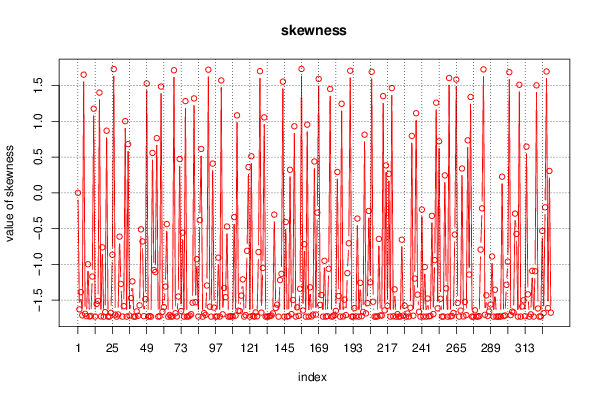
 **Figure 11:** The line plot of the skewness of the integers a, b, c for the Pythagorean triples
