# Supplementary material for: Datasets on the statistical and algebraic properties of primitive Pythagorean triples
Source: Data Brief. 2017 Sep 1;14:686–94. doi: 10.1016/j.dib.2017.08.021 (PMC5596336; doi:10.1016/j.dib.2017.08.021)
Supplement: Supplementary file 1 — Transparency document [file mmc2.zip › Supplementary Data 4.docx]

**Supplementary Data 4:** The tabulations of sine, cosine and tangent of a, b and c.

|  | sine a | sine b | sine c | cosine a | cosine b | cosine c | tan a | tan b | tan c |
| --- | --- | --- | --- | --- | --- | --- | --- | --- | --- |
| 1 | 0.0523 | 0.0698 | 0.0872 | 0.9986 | 0.9976 | 0.9962 | 0.0524 | 0.0699 | 0.0875 |
| 2 | 0.0872 | 0.2079 | 0.225 | 0.9962 | 0.9781 | 0.9744 | 0.0875 | 0.2126 | 0.2309 |
| 3 | 0.1392 | 0.2588 | 0.2924 | 0.9903 | 0.9659 | 0.9563 | 0.1405 | 0.2679 | 0.3057 |
| 4 | 0.1219 | 0.4067 | 0.4226 | 0.9925 | 0.9135 | 0.9063 | 0.1228 | 0.4452 | 0.4663 |
| 5 | 0.342 | 0.3584 | 0.4848 | 0.9397 | 0.9336 | 0.8746 | 0.364 | 0.3839 | 0.5543 |
| 6 | 0.2079 | 0.5736 | 0.6018 | 0.9781 | 0.8192 | 0.7986 | 0.2126 | 0.7002 | 0.7536 |
| 7 | 0.1564 | 0.6428 | 0.6561 | 0.9877 | 0.766 | 0.7547 | 0.1584 | 0.8391 | 0.8693 |
| 8 | 0.4695 | 0.7071 | 0.7986 | 0.8829 | 0.7071 | 0.6018 | 0.5317 | 1 | 1.327 |
| 9 | 0.1908 | 0.866 | 0.8746 | 0.9816 | 0.5 | 0.4848 | 0.1944 | 1.7321 | 1.804 |
| 10 | 0.2756 | 0.891 | 0.9063 | 0.9613 | 0.454 | 0.4226 | 0.2867 | 1.9626 | 2.1445 |
| 11 | 0.5446 | 0.829 | 0.9063 | 0.8387 | 0.5592 | 0.4226 | 0.6494 | 1.4826 | 2.1445 |
| 12 | 0.7431 | 0.8192 | 0.9563 | 0.6691 | 0.5736 | 0.2924 | 1.1106 | 1.4281 | 3.2709 |
| 13 | 0.225 | 0.9945 | 0.9962 | 0.9744 | 0.1045 | 0.0872 | 0.2309 | 9.5144 | 11.4301 |
| 14 | 0.5878 | 0.9744 | 0.9962 | 0.809 | 0.225 | 0.0872 | 0.7265 | 4.3315 | 11.4301 |
| 15 | 0.6293 | 0.9848 | 0.9998 | 0.7771 | 0.1736 | 0.0175 | 0.8098 | 5.6713 | 57.29 |
| 16 | 0.9063 | 0.9511 | 0.9925 | 0.4226 | 0.309 | -0.1219 | 2.1445 | 3.0777 | -8.1443 |
| 17 | 0.342 | 0.9877 | 0.9816 | 0.9397 | -0.1564 | -0.1908 | 0.364 | -6.3138 | -5.1446 |
| 18 | 0.866 | 0.9998 | 0.9455 | 0.5 | -0.0175 | -0.3256 | 1.7321 | -57.29 | -2.9042 |
| 19 | 0.2588 | 0.9272 | 0.9205 | 0.9659 | -0.3746 | -0.3907 | 0.2679 | -2.4751 | -2.3559 |
| 20 | 0.6947 | 0.891 | 0.8192 | 0.7193 | -0.454 | -0.5736 | 0.9657 | -1.9626 | -1.4281 |
| 21 | 0.9994 | 0.9659 | 0.682 | 0.0349 | -0.2588 | -0.7314 | 28.6363 | -3.7321 | -0.9325 |
| 22 | 0.2924 | 0.5878 | 0.5736 | 0.9563 | -0.809 | -0.8192 | 0.3057 | -0.7265 | -0.7002 |
| 23 | 0.4067 | 0.6018 | 0.5736 | 0.9135 | -0.7986 | -0.8192 | 0.4452 | -0.7536 | -0.7002 |
| 24 | 0.7771 | 0.6428 | 0.515 | 0.6293 | -0.766 | -0.8572 | 1.2349 | -0.8391 | -0.6009 |
| 25 | 0.9962 | 0.7431 | 0.3907 | 0.0872 | -0.6691 | -0.9205 | 11.4301 | -1.1106 | -0.4245 |
| 26 | 0.8746 | 0.866 | 0.1908 | -0.4848 | -0.5 | -0.9816 | -1.804 | -1.7321 | -0.1944 |
| 27 | 0.788 | 0.2588 | 0.1219 | 0.6157 | -0.9659 | -0.9925 | 1.2799 | -0.2679 | -0.1228 |
| 28 | 0.3256 | 0 | -0.0175 | 0.9455 | -1 | -0.9998 | 0.3443 | 0 | 0.0175 |
| 29 | 0.8387 | 0.0698 | -0.0872 | 0.5446 | -0.9976 | -0.9962 | 1.5399 | -0.0699 | 0.0875 |
| 30 | 0.9703 | 0.454 | -0.0872 | -0.2419 | -0.891 | -0.9962 | -4.0108 | -0.5095 | 0.0875 |
| 31 | 0.9962 | 0.2079 | -0.225 | -0.0872 | -0.9781 | -0.9744 | -11.4301 | -0.2126 | 0.2309 |
| 32 | 0.4695 | -0.2588 | -0.2924 | 0.8829 | -0.9659 | -0.9563 | 0.5317 | 0.2679 | 0.3057 |
| 33 | 0.9945 | -0.1219 | -0.4226 | 0.1045 | -0.9925 | -0.9063 | 9.5144 | 0.1228 | 0.4663 |
| 34 | 0.7314 | 0.4067 | -0.4226 | -0.682 | -0.9135 | -0.9063 | -1.0724 | -0.4452 | 0.4663 |
| 35 | 0.3584 | -0.6428 | -0.6561 | 0.9336 | -0.766 | -0.7547 | 0.3839 | 0.8391 | 0.8693 |
| 36 | 0.6428 | 0.1564 | -0.6561 | -0.766 | -0.9877 | -0.7547 | -0.8391 | -0.1584 | 0.8693 |
| 37 | 0.866 | -0.6561 | -0.7547 | 0.5 | -0.7547 | -0.6561 | 1.7321 | 0.8693 | 1.1504 |
| 38 | 0.9659 | -0.4695 | -0.7986 | -0.2588 | -0.8829 | -0.6018 | -3.7321 | 0.5317 | 1.327 |
| 39 | 0.866 | -0.4848 | -0.8746 | -0.5 | -0.8746 | -0.4848 | -1.7321 | 0.5543 | 1.804 |
| 40 | 0.5299 | -0.9659 | -0.9744 | 0.848 | -0.2588 | -0.225 | 0.6249 | 3.7321 | 4.3315 |
| 41 | 0.3907 | -0.9945 | -0.9962 | 0.9205 | -0.1045 | -0.0872 | 0.4245 | 9.5144 | 11.4301 |
| 42 | 0.9945 | -0.9205 | -0.9962 | -0.1045 | -0.3907 | -0.0872 | -9.5144 | 2.3559 | 11.4301 |
| 43 | 0.9336 | -0.9848 | -0.9998 | 0.3584 | -0.1736 | -0.0175 | 2.6051 | 5.6713 | 57.29 |
| 44 | 0.9063 | -0.9511 | -0.9925 | -0.4226 | -0.309 | 0.1219 | -2.1445 | 3.0777 | -8.1443 |
| 45 | 0.342 | -0.7771 | -0.9816 | -0.9397 | -0.6293 | 0.1908 | -0.364 | 1.2349 | -5.1446 |
| 46 | 0.3256 | -0.866 | -0.9455 | -0.9455 | -0.5 | 0.3256 | -0.3443 | 1.7321 | -2.9042 |
| 47 | 0.9272 | -0.9659 | -0.9205 | 0.3746 | 0.2588 | 0.3907 | 2.4751 | -3.7321 | -2.3559 |
| 48 | 0.6947 | -0.9986 | -0.8192 | -0.7193 | 0.0523 | 0.5736 | -0.9657 | -19.0811 | -1.4281 |
| 49 | -0.454 | -0.6947 | -0.8192 | -0.891 | -0.7193 | 0.5736 | 0.5095 | 0.9657 | -1.4281 |
| 50 | 0.4226 | -0.7431 | -0.7314 | 0.9063 | 0.6691 | 0.682 | 0.4663 | -1.1106 | -1.0724 |
| 51 | 0.9659 | -0.788 | -0.682 | 0.2588 | 0.6157 | 0.7314 | 3.7321 | -1.2799 | -0.9325 |
| 52 | 0.5878 | -0.6018 | -0.5736 | 0.809 | 0.7986 | 0.8192 | 0.7265 | -0.7536 | -0.7002 |
| 53 | -0.4067 | -0.9563 | -0.5736 | -0.9135 | -0.2924 | 0.8192 | 0.4452 | 3.2709 | -0.7002 |
| 54 | 0.0872 | -0.9511 | -0.3907 | -0.9962 | 0.309 | 0.9205 | -0.0875 | -3.0777 | -0.4245 |
| 55 | 0 | -0.8746 | -0.1908 | -1 | 0.4848 | 0.9816 | 0 | -1.804 | -0.1944 |
| 56 | -0.7071 | -0.9994 | -0.1219 | -0.7071 | 0.0349 | 0.9925 | 1 | -28.6363 | -0.1228 |
| 57 | 0.454 | 0.0698 | 0.0872 | 0.891 | 0.9976 | 0.9962 | 0.5095 | 0.0699 | 0.0875 |
| 58 | 0.9703 | -0.0523 | 0.0872 | 0.2419 | 0.9986 | 0.9962 | 4.0108 | -0.0524 | 0.0875 |
| 59 | -0.9511 | -0.9962 | 0.225 | -0.309 | 0.0872 | 0.9744 | 3.0777 | -11.4301 | 0.2309 |
| 60 | 0.7071 | -0.1392 | 0.2924 | -0.7071 | 0.9903 | 0.9563 | -1 | -0.1405 | 0.3057 |
| 61 | 0.4695 | -0.2588 | 0.2924 | -0.8829 | 0.9659 | 0.9563 | -0.5317 | -0.2679 | 0.3057 |
| 62 | -0.1564 | -0.342 | 0.4848 | -0.9877 | 0.9397 | 0.8746 | 0.1584 | -0.364 | 0.5543 |
| 63 | -0.7431 | -0.5736 | 0.6018 | -0.6691 | 0.8192 | 0.7986 | 1.1106 | -0.7002 | 0.7536 |
| 64 | 0.6428 | 0.6293 | 0.6561 | 0.766 | 0.7771 | 0.7547 | 0.8391 | 0.8098 | 0.8693 |
| 65 | 0.866 | 0.515 | 0.7547 | -0.5 | 0.8572 | 0.6561 | -1.7321 | 0.6009 | 1.1504 |
| 66 | 0.4848 | 0.866 | 0.8746 | 0.8746 | 0.5 | 0.4848 | 0.5543 | 1.7321 | 1.804 |
| 67 | 0.9986 | 0.829 | 0.9063 | 0.0523 | 0.5592 | 0.4226 | 19.0811 | 1.4826 | 2.1445 |
| 68 | -0.891 | -0.829 | 0.9063 | 0.454 | 0.5592 | 0.4226 | -1.9626 | -1.4826 | 2.1445 |
| 69 | 0.5736 | 0.7431 | 0.9563 | -0.8192 | 0.6691 | 0.2924 | -0.7002 | 1.1106 | 3.2709 |
| 70 | 0.9945 | 0.9744 | 0.9962 | 0.1045 | 0.225 | 0.0872 | 9.5144 | 4.3315 | 11.4301 |
| 71 | -0.3907 | 0.5878 | 0.9962 | -0.9205 | 0.809 | 0.0872 | 0.4245 | 0.7265 | 11.4301 |
| 72 | -0.9848 | -0.1564 | 0.9998 | 0.1736 | 0.9877 | 0.0175 | -5.6713 | -0.1584 | 57.29 |
| 73 | 0.2079 | 0.9063 | 0.9925 | -0.9781 | 0.4226 | -0.1219 | -0.2126 | 2.1445 | -8.1443 |
| 74 | -0.9877 | 0.342 | 0.9816 | -0.1564 | 0.9397 | -0.1908 | 6.3138 | 0.364 | -5.1446 |
| 75 | 0.515 | 0.866 | 0.8572 | 0.8572 | -0.5 | -0.515 | 0.6009 | -1.7321 | -1.6643 |
| 76 | -0.6561 | 0 | 0.8572 | 0.7547 | 1 | -0.515 | -0.8693 | 0 | -1.6643 |
| 77 | 0.6947 | 0.8387 | 0.8192 | 0.7193 | -0.5446 | -0.5736 | 0.9657 | -1.5399 | -1.4281 |
| 78 | 0.9986 | 0.8988 | 0.8192 | -0.0523 | -0.4384 | -0.5736 | -19.0811 | -2.0503 | -1.4281 |
| 79 | 0.7431 | 0.9063 | 0.7314 | -0.6691 | -0.4226 | -0.682 | -1.1106 | -2.1445 | -1.0724 |
| 80 | 0.4226 | 0.9511 | 0.7314 | -0.9063 | -0.309 | -0.682 | -0.4663 | -3.0777 | -1.0724 |
| 81 | -0.6018 | 0.9945 | 0.5736 | -0.7986 | -0.1045 | -0.8192 | 0.7536 | -9.5144 | -0.7002 |
| 82 | -0.4067 | 0.2924 | 0.5736 | 0.9135 | 0.9563 | -0.8192 | -0.4452 | 0.3057 | -0.7002 |
| 83 | -0.6428 | 0.9877 | 0.515 | -0.766 | -0.1564 | -0.8572 | 0.8391 | -6.3138 | -0.6009 |
| 84 | -0.9877 | 0.9848 | 0.3256 | 0.1564 | 0.1736 | -0.9455 | -6.3138 | 5.6713 | -0.3443 |
| 85 | 0.9994 | 0.2588 | 0.1219 | -0.0349 | -0.9659 | -0.9925 | -28.6363 | -0.2679 | -0.1228 |
| 86 | -0.788 | 0.9659 | 0.1219 | 0.6157 | 0.2588 | -0.9925 | -1.2799 | 3.7321 | -0.1228 |
| 87 | -0.3256 | 0.866 | -0.0175 | 0.9455 | 0.5 | -0.9998 | -0.3443 | 1.7321 | 0.0175 |
| 88 | 0.5446 | -0.0698 | -0.0872 | 0.8387 | -0.9976 | -0.9962 | 0.6494 | 0.0699 | 0.0875 |
| 89 | -0.0698 | 0.454 | -0.0872 | -0.9976 | -0.891 | -0.9962 | 0.0699 | -0.5095 | 0.0875 |
| 90 | 0.2588 | 0.1392 | -0.2924 | -0.9659 | -0.9903 | -0.9563 | -0.2679 | -0.1405 | 0.3057 |
| 91 | -0.9945 | 0.7314 | -0.4226 | 0.1045 | -0.682 | -0.9063 | -9.5144 | -1.0724 | 0.4663 |
| 92 | 0.5878 | 0.682 | -0.4226 | 0.809 | 0.7314 | -0.9063 | 0.7265 | 0.9325 | 0.4663 |
| 93 | -0.7771 | 0.342 | -0.4848 | -0.6293 | -0.9397 | -0.8746 | 1.2349 | -0.364 | 0.5543 |
| 94 | 0.7431 | -0.5736 | -0.6018 | 0.6691 | -0.8192 | -0.7986 | 1.1106 | 0.7002 | 0.7536 |
| 95 | 0.1392 | 0.9659 | -0.7986 | 0.9903 | -0.2588 | -0.6018 | 0.1405 | -3.7321 | 1.327 |
| 96 | -0.866 | -0.1908 | -0.8746 | -0.5 | -0.9816 | -0.4848 | 1.7321 | 0.1944 | 1.804 |
| 97 | 0.5736 | -0.9511 | -0.9563 | 0.8192 | -0.309 | -0.2924 | 0.7002 | 3.0777 | 3.2709 |
| 98 | 0.9659 | -0.9272 | -0.9744 | -0.2588 | -0.3746 | -0.225 | -3.7321 | 2.4751 | 4.3315 |
| 99 | -0.4067 | 0.225 | -0.9962 | 0.9135 | -0.9744 | -0.0872 | -0.4452 | -0.2309 | 11.4301 |
| 100 | 0.9848 | -0.9877 | -0.9998 | -0.1736 | -0.1564 | -0.0175 | -5.6713 | 6.3138 | 57.29 |
| 101 | 0.9336 | 0.9848 | -0.9998 | 0.3584 | -0.1736 | -0.0175 | 2.6051 | -5.6713 | 57.29 |
| 102 | -0.342 | -0.9336 | -0.9816 | -0.9397 | -0.3584 | 0.1908 | 0.364 | 2.6051 | -5.1446 |
| 103 | -0.7071 | -0.5299 | -0.9205 | 0.7071 | -0.848 | 0.3907 | -1 | 0.6249 | -2.3559 |
| 104 | -0.866 | -0.7547 | -0.8572 | 0.5 | -0.6561 | 0.515 | -1.7321 | 1.1504 | -1.6643 |
| 105 | 0.4226 | -0.2079 | -0.7314 | 0.9063 | -0.9781 | 0.682 | 0.4663 | 0.2126 | -1.0724 |
| 106 | 0.788 | -0.7071 | -0.682 | 0.6157 | 0.7071 | 0.7314 | 1.2799 | -1 | -0.9325 |
| 107 | 0.6018 | -0.5878 | -0.5736 | 0.7986 | 0.809 | 0.8192 | 0.7536 | -0.7265 | -0.7002 |
| 108 | 0.4067 | -0.7986 | -0.5736 | -0.9135 | 0.6018 | 0.8192 | -0.4452 | -1.327 | -0.7002 |
| 109 | 0.9336 | -0.6428 | -0.515 | -0.3584 | 0.766 | 0.8572 | -2.6051 | -0.8391 | -0.6009 |
| 110 | 0.6428 | -0.3584 | -0.515 | 0.766 | -0.9336 | 0.8572 | 0.8391 | 0.3839 | -0.6009 |
| 111 | -0.0872 | -0.7431 | -0.3907 | -0.9962 | 0.6691 | 0.9205 | 0.0875 | -1.1106 | -0.4245 |
| 112 | 0.9962 | 0.2079 | -0.3907 | -0.0872 | -0.9781 | 0.9205 | -11.4301 | -0.2126 | -0.4245 |
| 113 | -0.9848 | -0.9336 | -0.3256 | -0.1736 | 0.3584 | 0.9455 | 5.6713 | -2.6051 | -0.3443 |
| 114 | -0.9816 | -0.866 | -0.1908 | -0.1908 | 0.5 | 0.9816 | 5.1446 | -1.7321 | -0.1944 |
| 115 | -0.454 | -0.9703 | 0.0872 | 0.891 | 0.2419 | 0.9962 | -0.5095 | -4.0108 | 0.0875 |
| 116 | 0.0698 | -0.9986 | 0.0872 | 0.9976 | -0.0523 | 0.9962 | 0.0699 | 19.0811 | 0.0875 |
| 117 | 0.9511 | 0.0872 | 0.225 | -0.309 | 0.9962 | 0.9744 | -3.0777 | 0.0875 | 0.2309 |
| 118 | -0.5878 | -0.1219 | 0.4226 | -0.809 | 0.9925 | 0.9063 | 0.7265 | -0.1228 | 0.4663 |
| 119 | 0.7314 | -0.9945 | 0.4226 | 0.682 | -0.1045 | 0.9063 | 1.0724 | 9.5144 | 0.4663 |
| 120 | 0.9511 | -0.8192 | 0.6018 | -0.309 | -0.5736 | 0.7986 | -3.0777 | 1.4281 | 0.7536 |
| 121 | 0.6293 | 0.6428 | 0.6561 | 0.7771 | 0.766 | 0.7547 | 0.8098 | 0.8391 | 0.8693 |
| 122 | 0.8572 | -0.866 | 0.7547 | -0.515 | -0.5 | 0.6561 | -1.6643 | 1.7321 | 1.1504 |
| 123 | -0.2588 | 0.4695 | 0.7986 | -0.9659 | 0.8829 | 0.6018 | 0.2679 | 0.5317 | 1.327 |
| 124 | 0.829 | 0.891 | 0.9063 | 0.5592 | 0.454 | 0.4226 | 1.4826 | 1.9626 | 2.1445 |
| 125 | -0.9986 | 0.2756 | 0.9063 | 0.0523 | 0.9613 | 0.4226 | -19.0811 | 0.2867 | 2.1445 |
| 126 | 0.2079 | 0.8192 | 0.9563 | -0.9781 | 0.5736 | 0.2924 | -0.2126 | 1.4281 | 3.2709 |
| 127 | 0.9511 | -0.8192 | 0.9563 | 0.309 | 0.5736 | 0.2924 | 3.0777 | -1.4281 | 3.2709 |
| 128 | -0.2588 | -0.5299 | 0.9744 | -0.9659 | -0.848 | 0.225 | 0.2679 | 0.6249 | 4.3315 |
| 129 | -0.9848 | 0.6293 | 0.9998 | 0.1736 | 0.7771 | 0.0175 | -5.6713 | 0.8098 | 57.29 |
| 130 | 0.9336 | -0.342 | 0.9816 | 0.3584 | 0.9397 | -0.1908 | 2.6051 | -0.364 | -5.1446 |
| 131 | 0 | -0.9998 | 0.9455 | -1 | -0.0175 | -0.3256 | 0 | 57.29 | -2.9042 |
| 132 | 0.6561 | 0.866 | 0.8572 | 0.7547 | -0.5 | -0.515 | 0.8693 | -1.7321 | -1.6643 |
| 133 | 0.8988 | 0.891 | 0.8192 | -0.4384 | -0.454 | -0.5736 | -2.0503 | -1.9626 | -1.4281 |
| 134 | 0.8387 | 0.8988 | 0.8192 | -0.5446 | -0.4384 | -0.5736 | -1.5399 | -2.0503 | -1.4281 |
| 135 | -0.4226 | 0.9511 | 0.7314 | -0.9063 | -0.309 | -0.682 | 0.4663 | -3.0777 | -1.0724 |
| 136 | -0.788 | 0.9659 | 0.682 | -0.6157 | -0.2588 | -0.7314 | 1.2799 | -3.7321 | -0.9325 |
| 137 | -0.9563 | 0.9945 | 0.5736 | 0.2924 | -0.1045 | -0.8192 | -3.2709 | -9.5144 | -0.7002 |
| 138 | 0.5878 | -0.2924 | 0.5736 | -0.809 | 0.9563 | -0.8192 | -0.7265 | -0.3057 | -0.7002 |
| 139 | -0.2079 | 0.9962 | 0.3907 | 0.9781 | 0.0872 | -0.9205 | -0.2126 | 11.4301 | -0.4245 |
| 140 | 0.1564 | 0.9848 | 0.3256 | 0.9877 | 0.1736 | -0.9455 | 0.1584 | 5.6713 | -0.3443 |
| 141 | 0.866 | 0.0175 | -0.0175 | 0.5 | -0.9998 | -0.9998 | 1.7321 | -0.0175 | 0.0175 |
| 142 | 0.9998 | 0.866 | -0.0175 | -0.0175 | 0.5 | -0.9998 | -57.29 | 1.7321 | 0.0175 |
| 143 | 0.9703 | 0.8387 | -0.0872 | -0.2419 | 0.5446 | -0.9962 | -4.0108 | 1.5399 | 0.0875 |
| 144 | -0.9703 | -0.8387 | -0.0872 | -0.2419 | 0.5446 | -0.9962 | 4.0108 | -1.5399 | 0.0875 |
| 145 | 0.682 | -0.4067 | -0.4226 | 0.7314 | -0.9135 | -0.9063 | 0.9325 | 0.4452 | 0.4663 |
| 146 | 0.1219 | 0.5878 | -0.4226 | -0.9925 | 0.809 | -0.9063 | -0.1228 | 0.7265 | 0.4663 |
| 147 | 0.7771 | -0.342 | -0.4848 | -0.6293 | -0.9397 | -0.8746 | -1.2349 | 0.364 | 0.5543 |
| 148 | -0.5736 | -0.2079 | -0.6018 | -0.8192 | -0.9781 | -0.7986 | 0.7002 | 0.2126 | 0.7536 |
| 149 | -0.6428 | 0.3584 | -0.6561 | -0.766 | 0.9336 | -0.7547 | 0.8391 | 0.3839 | 0.8693 |
| 150 | -0.8572 | 0 | -0.7547 | 0.515 | -1 | -0.6561 | -1.6643 | 0 | 1.1504 |
| 151 | 0.866 | 0.7547 | -0.7547 | 0.5 | -0.6561 | -0.6561 | 1.7321 | -1.1504 | 1.1504 |
| 152 | -0.9659 | 0.1392 | -0.7986 | -0.2588 | 0.9903 | -0.6018 | 3.7321 | 0.1405 | 1.327 |
| 153 | 0.829 | -0.8387 | -0.9063 | -0.5592 | -0.5446 | -0.4226 | -1.4826 | 1.5399 | 2.1445 |
| 154 | 0.454 | 0.2756 | -0.9063 | 0.891 | -0.9613 | -0.4226 | 0.5095 | -0.2867 | 2.1445 |
| 155 | -0.9272 | -0.7071 | -0.9744 | -0.3746 | -0.7071 | -0.225 | 2.4751 | 1 | 4.3315 |
| 156 | 0.9205 | 0.5878 | -0.9962 | -0.3907 | -0.809 | -0.0872 | -2.3559 | -0.7265 | 11.4301 |
| 157 | -0.4067 | -0.3907 | -0.9962 | 0.9135 | 0.9205 | -0.0872 | -0.4452 | -0.4245 | 11.4301 |
| 158 | 0.2079 | -0.4226 | -0.9925 | 0.9781 | -0.9063 | 0.1219 | 0.2126 | 0.4663 | -8.1443 |
| 159 | -0.3256 | 0.866 | -0.9455 | -0.9455 | -0.5 | 0.3256 | 0.3443 | -1.7321 | -2.9042 |
| 160 | 0.7071 | -0.9272 | -0.9205 | 0.7071 | 0.3746 | 0.3907 | 1 | -2.4751 | -2.3559 |
| 161 | -0.866 | 0.8572 | -0.8572 | 0.5 | 0.515 | 0.515 | -1.7321 | 1.6643 | -1.6643 |
| 162 | 0.8988 | -0.8387 | -0.8192 | 0.4384 | 0.5446 | 0.5736 | 2.0503 | -1.5399 | -1.4281 |
| 163 | 0.6947 | 0.0523 | -0.8192 | -0.7193 | -0.9986 | 0.5736 | -0.9657 | -0.0524 | -1.4281 |
| 164 | -0.2079 | -0.9063 | -0.7314 | -0.9781 | 0.4226 | 0.682 | 0.2126 | -2.1445 | -1.0724 |
| 165 | -0.7071 | -0.9994 | -0.682 | 0.7071 | -0.0349 | 0.7314 | -1 | 28.6363 | -0.9325 |
| 166 | -0.9659 | 0.9994 | -0.682 | 0.2588 | -0.0349 | 0.7314 | -3.7321 | -28.6363 | -0.9325 |
| 167 | -0.6428 | -0.9877 | -0.515 | 0.766 | 0.1564 | 0.8572 | -0.8391 | -6.3138 | -0.6009 |
| 168 | -0.9848 | 0.6293 | -0.3256 | -0.1736 | -0.7771 | 0.9455 | 5.6713 | -0.8098 | -0.3443 |
| 169 | 0.1908 | 0.866 | -0.1908 | 0.9816 | 0.5 | 0.9816 | 0.1944 | 1.7321 | -0.1944 |
| 170 | 0.9994 | -0.9659 | -0.1219 | 0.0349 | -0.2588 | 0.9925 | 28.6363 | 3.7321 | -0.1228 |
| 171 | 0.7071 | -0.788 | -0.1219 | -0.7071 | -0.6157 | 0.9925 | -1 | 1.2799 | -0.1228 |
| 172 | 0.7431 | 0.0872 | 0.225 | -0.6691 | 0.9962 | 0.9744 | -1.1106 | 0.0875 | 0.2309 |
| 173 | -0.7071 | -0.4695 | 0.2924 | -0.7071 | -0.8829 | 0.9563 | 1 | 0.5317 | 0.3057 |
| 174 | 0.7314 | 0.4067 | 0.4226 | 0.682 | 0.9135 | 0.9063 | 1.0724 | 0.4452 | 0.4663 |
| 175 | -0.9945 | -0.1219 | 0.4226 | -0.1045 | 0.9925 | 0.9063 | 9.5144 | -0.1228 | 0.4663 |
| 176 | -0.5878 | -0.682 | 0.4226 | -0.809 | -0.7314 | 0.9063 | 0.7265 | 0.9325 | 0.4663 |
| 177 | 0.4067 | 0.9925 | 0.4226 | 0.9135 | -0.1219 | 0.9063 | 0.4452 | -8.1443 | 0.4663 |
| 178 | 0.6293 | 0.342 | 0.4848 | -0.7771 | 0.9397 | 0.8746 | -0.8098 | 0.364 | 0.5543 |
| 179 | -0.8192 | 0.2079 | 0.6018 | -0.5736 | 0.9781 | 0.7986 | 1.4281 | 0.2126 | 0.7536 |
| 180 | -0.515 | 0 | 0.7547 | 0.8572 | 1 | 0.6561 | -0.6009 | 0 | 1.1504 |
| 181 | 0.891 | -0.2756 | 0.9063 | 0.454 | 0.9613 | 0.4226 | 1.9626 | -0.2867 | 2.1445 |
| 182 | -0.2756 | -0.0523 | 0.9063 | 0.9613 | -0.9986 | 0.4226 | -0.2867 | 0.0524 | 2.1445 |
| 183 | 0.2079 | -0.8192 | 0.9563 | -0.9781 | 0.5736 | 0.2924 | -0.2126 | -1.4281 | 3.2709 |
| 184 | 0.9272 | 0.9659 | 0.9744 | 0.3746 | 0.2588 | 0.225 | 2.4751 | 3.7321 | 4.3315 |
| 185 | 0.7071 | 0.5299 | 0.9744 | 0.7071 | -0.848 | 0.225 | 1 | -0.6249 | 4.3315 |
| 186 | -0.4067 | 0.9205 | 0.9962 | -0.9135 | 0.3907 | 0.0872 | 0.4452 | 2.3559 | 11.4301 |
| 187 | 0.3907 | -0.5878 | 0.9962 | -0.9205 | 0.809 | 0.0872 | -0.4245 | -0.7265 | 11.4301 |
| 188 | -0.342 | 0.7771 | 0.9816 | 0.9397 | 0.6293 | -0.1908 | -0.364 | 1.2349 | -5.1446 |
| 189 | -0.9455 | -0.866 | 0.9455 | -0.3256 | 0.5 | -0.3256 | 2.9042 | -1.7321 | -2.9042 |
| 190 | -0.866 | -0.9998 | 0.9455 | 0.5 | -0.0175 | -0.3256 | -1.7321 | 57.29 | -2.9042 |
| 191 | 0.9272 | 0.7071 | 0.9205 | -0.3746 | -0.7071 | -0.3907 | -2.4751 | -1 | -2.3559 |
| 192 | 0.7547 | 0.866 | 0.8572 | 0.6561 | -0.5 | -0.515 | 1.1504 | -1.7321 | -1.6643 |
| 193 | 0.5446 | 0.8988 | 0.8192 | -0.8387 | -0.4384 | -0.5736 | -0.6494 | -2.0503 | -1.4281 |
| 194 | 0.8988 | 0.454 | 0.8192 | -0.4384 | 0.891 | -0.5736 | -2.0503 | 0.5095 | -1.4281 |
| 195 | -0.9063 | 0.9511 | 0.7314 | -0.4226 | -0.309 | -0.682 | 2.1445 | -3.0777 | -1.0724 |
| 196 | -0.2588 | -0.9994 | 0.682 | 0.9659 | 0.0349 | -0.7314 | -0.2679 | -28.6363 | -0.9325 |
| 197 | 0.6428 | 0.6293 | 0.515 | -0.766 | -0.7771 | -0.8572 | -0.8391 | -0.8098 | -0.6009 |
| 198 | -0.9511 | -0.0872 | 0.3907 | -0.309 | 0.9962 | -0.9205 | 3.0777 | -0.0875 | -0.4245 |
| 199 | -0.9848 | 0.7771 | 0.3256 | 0.1736 | -0.6293 | -0.9455 | -5.6713 | -1.2349 | -0.3443 |
| 200 | 0.9877 | 0.9848 | 0.3256 | 0.1564 | 0.1736 | -0.9455 | 6.3138 | 5.6713 | -0.3443 |
| 201 | 0.9816 | -0.866 | 0.1908 | 0.1908 | -0.5 | -0.9816 | 5.1446 | 1.7321 | -0.1944 |
| 202 | 0.866 | 0.9455 | -0.0175 | 0.5 | -0.3256 | -0.9998 | 1.7321 | -2.9042 | 0.0175 |
| 203 | 0.0175 | 0.866 | -0.0175 | -0.9998 | 0.5 | -0.9998 | -0.0175 | 1.7321 | 0.0175 |
| 204 | 0.4695 | -0.7071 | -0.2924 | 0.8829 | 0.7071 | -0.9563 | 0.5317 | -1 | 0.3057 |
| 205 | -0.9925 | 0.5878 | -0.4226 | 0.1219 | 0.809 | -0.9063 | -8.1443 | 0.7265 | 0.4663 |
| 206 | 0.1219 | -0.4067 | -0.4226 | -0.9925 | -0.9135 | -0.9063 | -0.1228 | 0.4452 | 0.4663 |
| 207 | -0.342 | 0.9877 | -0.4848 | -0.9397 | 0.1564 | -0.8746 | 0.364 | 6.3138 | 0.5543 |
| 208 | 0.9511 | -0.5736 | -0.6018 | 0.309 | -0.8192 | -0.7986 | 3.0777 | 0.7002 | 0.7536 |
| 209 | 0.7771 | -0.6428 | -0.6561 | 0.6293 | -0.766 | -0.7547 | 1.2349 | 0.8391 | 0.8693 |
| 210 | -0.9659 | -0.4695 | -0.7986 | -0.2588 | -0.8829 | -0.6018 | 3.7321 | 0.5317 | 1.327 |
| 211 | 0.2588 | 0.1392 | -0.7986 | 0.9659 | 0.9903 | -0.6018 | 0.2679 | 0.1405 | 1.327 |
| 212 | 0 | -0.1908 | -0.8746 | 1 | -0.9816 | -0.4848 | 0 | 0.1944 | 1.804 |
| 213 | -0.0523 | -0.2756 | -0.9063 | 0.9986 | -0.9613 | -0.4226 | -0.0524 | 0.2867 | 2.1445 |
| 214 | 0.2756 | -0.9986 | -0.9063 | -0.9613 | -0.0523 | -0.4226 | -0.2867 | 19.0811 | 2.1445 |
| 215 | 0.5878 | 0.225 | -0.9962 | -0.809 | -0.9744 | -0.0872 | -0.7265 | -0.2309 | 11.4301 |
| 216 | 0.9205 | -0.4067 | -0.9962 | -0.3907 | 0.9135 | -0.0872 | -2.3559 | -0.4452 | 11.4301 |
| 217 | -0.3584 | 0.342 | -0.9816 | -0.9336 | -0.9397 | 0.1908 | 0.3839 | -0.364 | -5.1446 |
| 218 | 0.866 | 0.0175 | -0.9455 | -0.5 | 0.9998 | 0.3256 | -1.7321 | 0.0175 | -2.9042 |
| 219 | 0.5299 | -0.9659 | -0.9205 | -0.848 | 0.2588 | 0.3907 | -0.6249 | -3.7321 | -2.3559 |
| 220 | -0.515 | -0.866 | -0.8572 | -0.8572 | 0.5 | 0.515 | 0.6009 | -1.7321 | -1.6643 |
| 221 | -0.8988 | -0.9986 | -0.8192 | 0.4384 | 0.0523 | 0.5736 | -2.0503 | -19.0811 | -1.4281 |
| 222 | -0.8387 | 0.6947 | -0.8192 | 0.5446 | -0.7193 | 0.5736 | -1.5399 | -0.9657 | -1.4281 |
| 223 | 0.7986 | -0.5878 | -0.5736 | 0.6018 | 0.809 | 0.8192 | 1.327 | -0.7265 | -0.7002 |
| 224 | 0.9945 | -0.9563 | -0.5736 | 0.1045 | -0.2924 | 0.8192 | 9.5144 | 3.2709 | -0.7002 |
| 225 | 0.3584 | -0.6428 | -0.515 | -0.9336 | 0.766 | 0.8572 | -0.3839 | -0.8391 | -0.6009 |
| 226 | -0.9962 | -0.7431 | -0.3907 | -0.0872 | 0.6691 | 0.9205 | 11.4301 | -1.1106 | -0.4245 |
| 227 | 0.9511 | 0.9962 | -0.3907 | 0.309 | -0.0872 | 0.9205 | 3.0777 | -11.4301 | -0.4245 |
| 228 | 0.1908 | -0.866 | -0.1908 | 0.9816 | 0.5 | 0.9816 | 0.1944 | -1.7321 | -0.1944 |
| 229 | -0.788 | -0.7071 | -0.1219 | -0.6157 | -0.7071 | 0.9925 | 1.2799 | 1 | -0.1228 |
| 230 | 0.9703 | 0.0523 | 0.0872 | 0.2419 | 0.9986 | 0.9962 | 4.0108 | 0.0524 | 0.0875 |
| 231 | 0.891 | -0.9703 | 0.0872 | -0.454 | 0.2419 | 0.9962 | -1.9626 | -4.0108 | 0.0875 |
| 232 | -0.7431 | -0.0872 | 0.225 | -0.6691 | 0.9962 | 0.9744 | 1.1106 | -0.0875 | 0.2309 |
| 233 | -0.682 | -0.9945 | 0.4226 | -0.7314 | -0.1045 | 0.9063 | 0.9325 | 9.5144 | 0.4663 |
| 234 | -0.5878 | 0.7314 | 0.4226 | -0.809 | 0.682 | 0.9063 | 0.7265 | 1.0724 | 0.4663 |
| 235 | 0.342 | -0.3584 | 0.4848 | 0.9397 | 0.9336 | 0.8746 | 0.364 | -0.3839 | 0.5543 |
| 236 | 0.342 | -0.1564 | 0.4848 | 0.9397 | -0.9877 | 0.8746 | 0.364 | 0.1584 | 0.5543 |
| 237 | -0.9336 | 0.6428 | 0.6561 | -0.3584 | 0.766 | 0.7547 | 2.6051 | 0.8391 | 0.8693 |
| 238 | -0.515 | -0.866 | 0.7547 | 0.8572 | -0.5 | 0.6561 | -0.6009 | 1.7321 | 1.1504 |
| 239 | 0.1392 | -0.7071 | 0.7986 | -0.9903 | 0.7071 | 0.6018 | -0.1405 | -1 | 1.327 |
| 240 | 0.8192 | 0.9511 | 0.9563 | 0.5736 | 0.309 | 0.2924 | 1.4281 | 3.0777 | 3.2709 |
| 241 | 0.2079 | 0.5736 | 0.9563 | -0.9781 | -0.8192 | 0.2924 | -0.2126 | -0.7002 | 3.2709 |
| 242 | 0.2588 | 0.9272 | 0.9744 | -0.9659 | 0.3746 | 0.225 | -0.2679 | 2.4751 | 4.3315 |
| 243 | 0.9659 | -0.5299 | 0.9744 | 0.2588 | -0.848 | 0.225 | 3.7321 | 0.6249 | 4.3315 |
| 244 | 0.4067 | 0.9744 | 0.9962 | -0.9135 | 0.225 | 0.0872 | -0.4452 | 4.3315 | 11.4301 |
| 245 | -0.5878 | -0.9744 | 0.9962 | 0.809 | 0.225 | 0.0872 | -0.7265 | -4.3315 | 11.4301 |
| 246 | -0.7431 | 0.9063 | 0.9925 | 0.6691 | 0.4226 | -0.1219 | -1.1106 | 2.1445 | -8.1443 |
| 247 | 0.4226 | 0.7431 | 0.9925 | 0.9063 | 0.6691 | -0.1219 | 0.4663 | 1.1106 | -8.1443 |
| 248 | -0.0175 | 0 | 0.9455 | -0.9998 | -1 | -0.3256 | 0.0175 | 0 | -2.9042 |
| 249 | 0.7071 | 0.5299 | 0.9205 | -0.7071 | 0.848 | -0.3907 | -1 | 0.6249 | -2.3559 |
| 250 | 0.8988 | -0.891 | 0.8192 | -0.4384 | -0.454 | -0.5736 | -2.0503 | 1.9626 | -1.4281 |
| 251 | -0.6947 | 0.9986 | 0.8192 | 0.7193 | -0.0523 | -0.5736 | -0.9657 | -19.0811 | -1.4281 |
| 252 | -0.9945 | 0.2924 | 0.5736 | -0.1045 | 0.9563 | -0.8192 | 9.5144 | 0.3057 | -0.7002 |
| 253 | -0.9563 | 0.5878 | 0.5736 | 0.2924 | -0.809 | -0.8192 | -3.2709 | -0.7265 | -0.7002 |
| 254 | -0.0872 | -0.2079 | 0.3907 | 0.9962 | 0.9781 | -0.9205 | -0.0875 | -0.2126 | -0.4245 |
| 255 | 0.9848 | 0.3584 | 0.3256 | 0.1736 | -0.9336 | -0.9455 | 5.6713 | -0.3839 | -0.3443 |
| 256 | -0.866 | 0.4848 | 0.1908 | -0.5 | -0.8746 | -0.9816 | 1.7321 | -0.5543 | -0.1944 |
| 257 | -0.9994 | -0.2588 | 0.1219 | -0.0349 | -0.9659 | -0.9925 | 28.6363 | 0.2679 | -0.1228 |
| 258 | 0.866 | -0.3256 | -0.0175 | 0.5 | 0.9455 | -0.9998 | 1.7321 | -0.3443 | 0.0175 |
| 259 | 0.8387 | -0.0698 | -0.0872 | 0.5446 | -0.9976 | -0.9962 | 1.5399 | 0.0699 | 0.0875 |
| 260 | 0.5446 | 0.9703 | -0.0872 | 0.8387 | -0.2419 | -0.9962 | 0.6494 | -4.0108 | 0.0875 |
| 261 | -0.9659 | 0.1392 | -0.2924 | 0.2588 | -0.9903 | -0.9563 | -3.7321 | -0.1405 | 0.3057 |
| 262 | 0.6293 | 0.342 | -0.4848 | 0.7771 | -0.9397 | -0.8746 | 0.8098 | -0.364 | 0.5543 |
| 263 | -0.342 | 0.9336 | -0.4848 | -0.9397 | -0.3584 | -0.8746 | 0.364 | -2.6051 | 0.5543 |
| 264 | -0.5736 | -0.9511 | -0.6018 | -0.8192 | 0.309 | -0.7986 | 0.7002 | -3.0777 | 0.7536 |
| 265 | 0.866 | 0.6561 | -0.7547 | 0.5 | -0.7547 | -0.6561 | 1.7321 | -0.8693 | 1.1504 |
| 266 | 0 | 0.9816 | -0.8746 | 1 | 0.1908 | -0.4848 | 0 | 5.1446 | 1.804 |
| 267 | 0.2756 | -0.8387 | -0.9063 | -0.9613 | -0.5446 | -0.4226 | -0.2867 | 1.5399 | 2.1445 |
| 268 | -0.9986 | 0.829 | -0.9063 | -0.0523 | -0.5592 | -0.4226 | 19.0811 | -1.4826 | 2.1445 |
| 269 | -0.5736 | -0.9511 | -0.9563 | 0.8192 | -0.309 | -0.2924 | -0.7002 | 3.0777 | 3.2709 |
| 270 | -0.5299 | -0.7071 | -0.9744 | 0.848 | -0.7071 | -0.225 | -0.6249 | 1 | 4.3315 |
| 271 | 0.3584 | 0.9848 | -0.9998 | 0.9336 | -0.1736 | -0.0175 | 0.3839 | -5.6713 | 57.29 |
| 272 | 0.7431 | -0.4226 | -0.9925 | -0.6691 | -0.9063 | 0.1219 | -1.1106 | 0.4663 | -8.1443 |
| 273 | 0.2079 | -0.9063 | -0.9925 | 0.9781 | -0.4226 | 0.1219 | 0.2126 | 2.1445 | -8.1443 |
| 274 | 0.342 | 0.6293 | -0.9816 | -0.9397 | 0.7771 | 0.1908 | -0.364 | 0.8098 | -5.1446 |
| 275 | 0.9659 | -0.5299 | -0.9205 | 0.2588 | -0.848 | 0.3907 | 3.7321 | 0.6249 | -2.3559 |
| 276 | 0.8572 | -0.866 | -0.8572 | 0.515 | 0.5 | 0.515 | 1.6643 | -1.7321 | -1.6643 |
| 277 | 0.0523 | -0.8988 | -0.8192 | -0.9986 | 0.4384 | 0.5736 | -0.0524 | -2.0503 | -1.4281 |
| 278 | -0.8988 | 0.0523 | -0.8192 | 0.4384 | -0.9986 | 0.5736 | -2.0503 | -0.0524 | -1.4281 |
| 279 | -0.9063 | -0.9511 | -0.7314 | 0.4226 | 0.309 | 0.682 | -2.1445 | -3.0777 | -1.0724 |
| 280 | 0.9945 | -0.6018 | -0.5736 | 0.1045 | 0.7986 | 0.8192 | 9.5144 | -0.7536 | -0.7002 |
| 281 | 0.7986 | -0.9945 | -0.5736 | 0.6018 | 0.1045 | 0.8192 | 1.327 | -9.5144 | -0.7002 |
| 282 | -0.9336 | 0.6428 | -0.515 | -0.3584 | 0.766 | 0.8572 | 2.6051 | 0.8391 | -0.6009 |
| 283 | -0.6428 | -0.1564 | -0.515 | 0.766 | 0.9877 | 0.8572 | -0.8391 | -0.1584 | -0.6009 |
| 284 | 0.2079 | -0.0872 | -0.3907 | -0.9781 | -0.9962 | 0.9205 | -0.2126 | 0.0875 | -0.4245 |
| 285 | 0.1564 | -0.9848 | -0.3256 | -0.9877 | -0.1736 | 0.9455 | -0.1584 | 5.6713 | -0.3443 |
| 286 | 0.9848 | 0.6293 | -0.3256 | -0.1736 | -0.7771 | 0.9455 | -5.6713 | -0.8098 | -0.3443 |
| 287 | 0.866 | -0.8746 | -0.1908 | 0.5 | 0.4848 | 0.9816 | 1.7321 | -1.804 | -0.1944 |
| 288 | -0.9455 | -0.866 | 0.0175 | 0.3256 | -0.5 | 0.9998 | -2.9042 | 1.7321 | 0.0175 |
| 289 | 0.7314 | -0.5878 | 0.4226 | 0.682 | -0.809 | 0.9063 | 1.0724 | 0.7265 | 0.4663 |
| 290 | -0.9945 | 0.9925 | 0.4226 | -0.1045 | -0.1219 | 0.9063 | 9.5144 | -8.1443 | 0.4663 |
| 291 | 0.1392 | 0.7071 | 0.7986 | -0.9903 | 0.7071 | 0.6018 | -0.1405 | 1 | 1.327 |
| 292 | 0.2588 | -0.1392 | 0.7986 | -0.9659 | -0.9903 | 0.6018 | -0.2679 | 0.1405 | 1.327 |
| 293 | 0.8746 | 0.866 | 0.8746 | 0.4848 | 0.5 | 0.4848 | 1.804 | 1.7321 | 1.804 |
| 294 | -0.0523 | 0.829 | 0.9063 | -0.9986 | 0.5592 | 0.4226 | 0.0524 | 1.4826 | 2.1445 |
| 295 | -0.2756 | 0.5446 | 0.9063 | 0.9613 | 0.8387 | 0.4226 | -0.2867 | 0.6494 | 2.1445 |
| 296 | -0.8192 | 0.7431 | 0.9563 | 0.5736 | 0.6691 | 0.2924 | -1.4281 | 1.1106 | 3.2709 |
| 297 | 0.9272 | 0.7071 | 0.9744 | 0.3746 | 0.7071 | 0.225 | 2.4751 | 1 | 4.3315 |
| 298 | 0.9205 | 0.5878 | 0.9962 | 0.3907 | 0.809 | 0.0872 | 2.3559 | 0.7265 | 11.4301 |
| 299 | 0.4067 | 0.225 | 0.9962 | -0.9135 | 0.9744 | 0.0872 | -0.4452 | 0.2309 | 11.4301 |
| 300 | -0.4067 | -0.3907 | 0.9962 | -0.9135 | -0.9205 | 0.0872 | 0.4452 | 0.4245 | 11.4301 |
| 301 | -0.9744 | 0.4067 | 0.9962 | 0.225 | -0.9135 | 0.0872 | -4.3315 | -0.4452 | 11.4301 |
| 302 | -0.7771 | -0.9848 | 0.9998 | -0.6293 | 0.1736 | 0.0175 | 1.2349 | -5.6713 | 57.29 |
| 303 | -0.1564 | 0.342 | 0.9816 | -0.9877 | 0.9397 | -0.1908 | 0.1584 | 0.364 | -5.1446 |
| 304 | -0.5299 | -0.2588 | 0.9205 | 0.848 | 0.9659 | -0.3907 | -0.6249 | -0.2679 | -2.3559 |
| 305 | -0.7547 | 0 | 0.8572 | 0.6561 | 1 | -0.515 | -1.1504 | 0 | -1.6643 |
| 306 | 0.6561 | 0.866 | 0.8572 | 0.7547 | -0.5 | -0.515 | 0.8693 | -1.7321 | -1.6643 |
| 307 | 0.2079 | 0.4226 | 0.7314 | 0.9781 | -0.9063 | -0.682 | 0.2126 | -0.4663 | -1.0724 |
| 308 | 0.9994 | 0.7071 | 0.682 | 0.0349 | -0.7071 | -0.7314 | 28.6363 | -1 | -0.9325 |
| 309 | -0.788 | -0.2588 | 0.682 | -0.6157 | 0.9659 | -0.7314 | 1.2799 | -0.2679 | -0.9325 |
| 310 | -0.9945 | 0.7986 | 0.5736 | -0.1045 | -0.6018 | -0.8192 | 9.5144 | -1.327 | -0.7002 |
| 311 | 0.9563 | -0.4067 | 0.5736 | 0.2924 | 0.9135 | -0.8192 | 3.2709 | -0.4452 | -0.7002 |
| 312 | 0.6428 | -0.7771 | 0.515 | -0.766 | 0.6293 | -0.8572 | -0.8391 | -1.2349 | -0.6009 |
| 313 | 0.9848 | 0.9336 | 0.3256 | 0.1736 | -0.3584 | -0.9455 | 5.6713 | -2.6051 | -0.3443 |
| 314 | 0.3584 | 0.9848 | 0.3256 | -0.9336 | 0.1736 | -0.9455 | -0.3839 | 5.6713 | -0.3443 |
| 315 | -0.2588 | -0.788 | 0.1219 | -0.9659 | 0.6157 | -0.9925 | 0.2679 | -1.2799 | -0.1228 |
| 316 | 0.891 | -0.0698 | -0.0872 | 0.454 | -0.9976 | -0.9962 | 1.9626 | 0.0699 | 0.0875 |
| 317 | -0.9703 | 0.9986 | -0.0872 | -0.2419 | 0.0523 | -0.9962 | 4.0108 | 19.0811 | 0.0875 |
| 318 | -0.7431 | -0.9962 | -0.225 | 0.6691 | -0.0872 | -0.9744 | -1.1106 | 11.4301 | 0.2309 |
| 319 | -0.7071 | 0.1392 | -0.2924 | 0.7071 | -0.9903 | -0.9563 | -1 | -0.1405 | 0.3057 |
| 320 | -0.682 | -0.9945 | -0.4226 | 0.7314 | 0.1045 | -0.9063 | -0.9325 | -9.5144 | 0.4663 |
| 321 | -0.9925 | 0.5878 | -0.4226 | 0.1219 | 0.809 | -0.9063 | -8.1443 | 0.7265 | 0.4663 |
| 322 | 0.9511 | 0.8192 | -0.6018 | 0.309 | 0.5736 | -0.7986 | 3.0777 | 1.4281 | 0.7536 |
| 323 | 0 | -0.6561 | -0.7547 | -1 | -0.7547 | -0.6561 | 0 | 0.8693 | 1.1504 |
| 324 | 0 | -0.4848 | -0.8746 | 1 | -0.8746 | -0.4848 | 0 | 0.5543 | 1.804 |
| 325 | 0.9816 | -0.866 | -0.8746 | 0.1908 | -0.5 | -0.4848 | 5.1446 | 1.7321 | 1.804 |
| 326 | -0.454 | 0.829 | -0.9063 | 0.891 | -0.5592 | -0.4226 | -0.5095 | -1.4826 | 2.1445 |
| 327 | 0.829 | -0.5446 | -0.9063 | -0.5592 | -0.8387 | -0.4226 | -1.4826 | 0.6494 | 2.1445 |
| 328 | -0.2079 | 0.5736 | -0.9563 | 0.9781 | 0.8192 | -0.2924 | -0.2126 | 0.7002 | 3.2709 |
| 329 | 0.9877 | 0.9848 | -0.9998 | -0.1564 | -0.1736 | -0.0175 | -6.3138 | -5.6713 | 57.29 |
| 330 | -0.3584 | -0.342 | -0.9816 | -0.9336 | -0.9397 | 0.1908 | 0.3839 | 0.364 | -5.1446 |
| 331 | 0 | 0.3256 | -0.9455 | 1 | -0.9455 | 0.3256 | 0 | -0.3443 | -2.9042 |
