# Supplementary material for: Datasets on the statistical and algebraic properties of primitive Pythagorean triples
Source: Data Brief. 2017 Sep 1;14:686–94. doi: 10.1016/j.dib.2017.08.021 (PMC5596336; doi:10.1016/j.dib.2017.08.021)
Supplement: Supplementary file 1 — Transparency document [file mmc2.zip › Supplementary Data 5.docx]

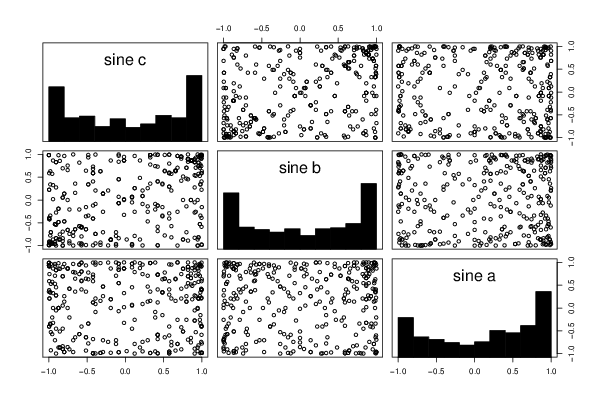


**Figure 12:** Summary of scatter plots of sine a, sine b and sine c


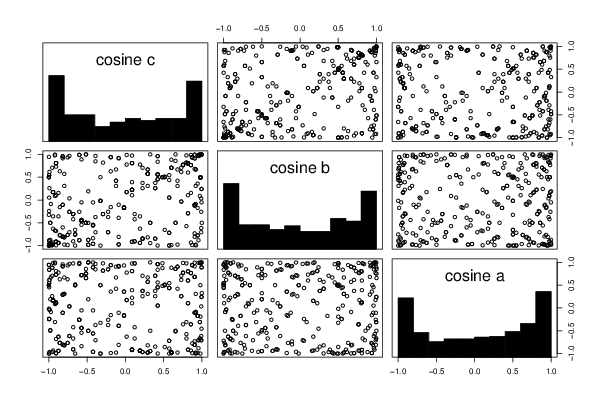


**Figure 13:** Summary of scatter plots of cosine a, cosine b and cosine c


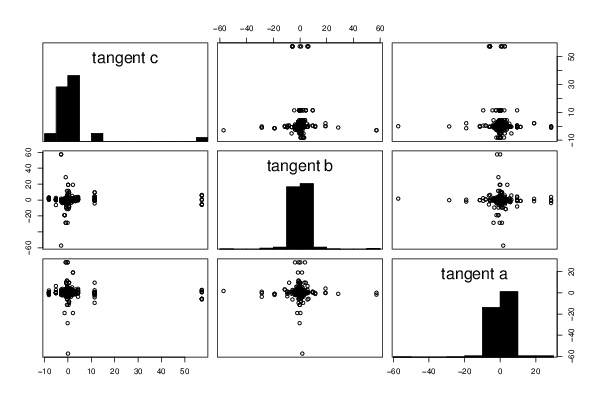


**Figure 14:** Summary of scatter plots of tangent a, tangent b and tangent c
