# Supplementary material for: Datasets on the statistical and algebraic properties of primitive Pythagorean triples
Source: Data Brief. 2017 Sep 1;14:686–94. doi: 10.1016/j.dib.2017.08.021 (PMC5596336; doi:10.1016/j.dib.2017.08.021)
Supplement: Supplementary file 1 — Transparency document [file mmc2.zip › Supplementary Data 6.docx]

**Supplementary Data 6:**  The tabulations of sinh, cosh and tanh of a, b and c.

|  | sinh a | sinh b | sinh c | cosh a | cosh b | cosh c | tanh a | tanh b | tanh c |
| --- | --- | --- | --- | --- | --- | --- | --- | --- | --- |
| 1 | 0.052384 | 0.06987 | 0.087377 | 1.001371 | 1.002438 | 1.00381 | 0.052312 | 0.0697 | 0.087046 |
| 2 | 0.087377 | 0.210974 | 0.228845 | 1.00381 | 1.022013 | 1.025851 | 0.087046 | 0.20643 | 0.223078 |
| 3 | 0.14008 | 0.2648 | 0.301079 | 1.009764 | 1.034466 | 1.044341 | 0.138726 | 0.255978 | 0.288295 |
| 4 | 0.122477 | 0.431236 | 0.45031 | 1.007472 | 1.08902 | 1.096713 | 0.121569 | 0.395986 | 0.4106 |
| 5 | 0.356198 | 0.374781 | 0.528035 | 1.061545 | 1.067923 | 1.13085 | 0.335547 | 0.350943 | 0.466937 |
| 6 | 0.210974 | 0.649572 | 0.6916 | 1.022013 | 1.192453 | 1.215858 | 0.20643 | 0.544736 | 0.568817 |
| 7 | 0.157726 | 0.75624 | 0.778238 | 1.012362 | 1.253754 | 1.267144 | 0.1558 | 0.603181 | 0.614167 |
| 8 | 0.508377 | 0.868671 | 1.062704 | 1.121805 | 1.324609 | 1.459226 | 0.453178 | 0.655794 | 0.728266 |
| 9 | 0.193168 | 1.249367 | 1.277489 | 1.018486 | 1.600287 | 1.622337 | 0.189662 | 0.780714 | 0.787437 |
| 10 | 0.282896 | 1.334909 | 1.393956 | 1.039245 | 1.667928 | 1.71555 | 0.272213 | 0.80034 | 0.812542 |
| 11 | 0.608335 | 1.140601 | 1.393956 | 1.1705 | 1.516895 | 1.71555 | 0.519722 | 0.751931 | 0.812542 |
| 12 | 0.93925 | 1.114299 | 1.647881 | 1.37193 | 1.497218 | 1.927566 | 0.68462 | 0.744246 | 0.854902 |
| 13 | 0.228845 | 2.050688 | 2.090822 | 1.025851 | 2.281517 | 2.317658 | 0.223078 | 0.898826 | 0.902127 |
| 14 | 0.670484 | 1.786577 | 2.090822 | 1.203972 | 2.047403 | 2.317658 | 0.556893 | 0.872607 | 0.902127 |
| 15 | 0.734472 | 1.896278 | 2.257854 | 1.240745 | 2.143798 | 2.469393 | 0.59196 | 0.884541 | 0.914335 |
| 16 | 1.393956 | 1.614488 | 2.625812 | 1.71555 | 1.899098 | 2.809785 | 0.812542 | 0.850134 | 0.934524 |
| 17 | 0.356198 | 2.725512 | 2.828533 | 1.061545 | 2.903174 | 3.0001 | 0.335547 | 0.938804 | 0.942813 |
| 18 | 1.249367 | 2.345445 | 3.276405 | 1.600287 | 2.549728 | 3.425614 | 0.780714 | 0.919881 | 0.956443 |
| 19 | 0.2648 | 3.460344 | 3.52374 | 1.034466 | 3.601941 | 3.662888 | 0.255978 | 0.960689 | 0.962012 |
| 20 | 0.845683 | 3.788256 | 4.374082 | 1.309649 | 3.918021 | 4.486936 | 0.645733 | 0.96688 | 0.974848 |
| 21 | 2.215096 | 3.045046 | 5.416994 | 2.430361 | 3.205043 | 5.508523 | 0.911427 | 0.950079 | 0.983384 |
| 22 | 0.301079 | 6.132141 | 6.24152 | 1.044341 | 6.213143 | 6.321121 | 0.288295 | 0.986963 | 0.987407 |
| 23 | 0.431236 | 6.024629 | 6.24152 | 1.08902 | 6.107058 | 6.321121 | 0.395986 | 0.986503 | 0.987407 |
| 24 | 1.012405 | 5.712979 | 6.698392 | 1.423012 | 5.799838 | 6.772626 | 0.711452 | 0.985024 | 0.989039 |
| 25 | 2.090822 | 4.956314 | 7.712505 | 2.317658 | 5.056189 | 7.777065 | 0.902127 | 0.980247 | 0.991699 |
| 26 | 3.927357 | 3.998691 | 9.523038 | 4.05267 | 4.121836 | 9.575398 | 0.969079 | 0.970124 | 0.994532 |
| 27 | 1.037396 | 8.877222 | 10.21529 | 1.4409 | 8.933369 | 10.26412 | 0.719964 | 0.993715 | 0.995243 |
| 28 | 0.337724 | 11.54874 | 11.75283 | 1.055489 | 11.59195 | 11.79529 | 0.319969 | 0.996272 | 0.9964 |
| 29 | 1.167251 | 10.76697 | 12.60561 | 1.537034 | 10.8133 | 12.64522 | 0.759418 | 0.995715 | 0.996868 |
| 30 | 2.989568 | 7.187925 | 12.60561 | 3.152383 | 7.257153 | 12.64522 | 0.948352 | 0.990461 | 0.996868 |
| 31 | 2.529312 | 9.357358 | 14.50004 | 2.71982 | 9.41064 | 14.53448 | 0.929956 | 0.994338 | 0.99763 |
| 32 | 0.508377 | 15.01633 | 15.55091 | 1.121805 | 15.04959 | 15.58303 | 0.453178 | 0.99779 | 0.997939 |
| 33 | 2.050688 | 13.05479 | 17.88562 | 2.281517 | 13.09303 | 17.91356 | 0.898826 | 0.997079 | 0.998441 |
| 34 | 5.04532 | 7.577938 | 17.88562 | 5.143468 | 7.643634 | 17.91356 | 0.980918 | 0.991405 | 0.998441 |
| 35 | 0.374781 | 23.24558 | 23.65522 | 1.067923 | 23.26708 | 23.67635 | 0.350943 | 0.999076 | 0.999108 |
| 36 | 5.712979 | 9.863152 | 23.65522 | 5.799838 | 9.913717 | 23.67635 | 0.985024 | 0.9949 | 0.999108 |
| 37 | 1.249367 | 23.65522 | 27.20278 | 1.600287 | 23.67635 | 27.22115 | 0.780714 | 0.999108 | 0.999325 |
| 38 | 3.045046 | 18.84853 | 29.17104 | 3.205043 | 18.87503 | 29.18817 | 0.950079 | 0.998596 | 0.999413 |
| 39 | 3.998691 | 19.18084 | 33.54454 | 4.121836 | 19.20689 | 33.55945 | 0.970124 | 0.998644 | 0.999556 |
| 40 | 0.587997 | 42.83289 | 44.35484 | 1.160061 | 42.84456 | 44.36612 | 0.506868 | 0.999728 | 0.999746 |
| 41 | 0.412294 | 50.12012 | 51.00273 | 1.081659 | 50.1301 | 51.01254 | 0.381168 | 0.999801 | 0.999808 |
| 42 | 2.57717 | 37.2494 | 51.00273 | 2.764381 | 37.26282 | 51.01254 | 0.932277 | 0.99964 | 0.999808 |
| 43 | 1.51722 | 46.73973 | 54.69132 | 1.817128 | 46.75042 | 54.70046 | 0.834955 | 0.999771 | 0.999833 |
| 44 | 3.653772 | 40.64725 | 62.88777 | 3.788146 | 40.65955 | 62.89572 | 0.964528 | 0.999698 | 0.999874 |
| 45 | 8.130472 | 28.16974 | 67.4356 | 8.191738 | 28.18749 | 67.44301 | 0.992521 | 0.999371 | 0.99989 |
| 46 | 8.274691 | 32.9639 | 77.54146 | 8.334897 | 32.97906 | 77.54791 | 0.992777 | 0.99954 | 0.999917 |
| 47 | 1.485734 | 72.31224 | 83.14877 | 1.790923 | 72.31915 | 83.15478 | 0.829591 | 0.999904 | 0.999928 |
| 48 | 5.321672 | 58.64656 | 102.5226 | 5.414813 | 58.65509 | 102.5275 | 0.982799 | 0.999855 | 0.999952 |
| 49 | 18.52195 | 24.92792 | 102.5226 | 18.54892 | 24.94797 | 102.5275 | 0.998546 | 0.999196 | 0.999952 |
| 50 | 0.45031 | 115.846 | 117.8857 | 1.096713 | 115.8503 | 117.8899 | 0.4106 | 0.999963 | 0.999964 |
| 51 | 1.716184 | 108.0339 | 126.41 | 1.986274 | 108.0386 | 126.414 | 0.864021 | 0.999957 | 0.999969 |
| 52 | 0.670484 | 140.366 | 145.3524 | 1.203972 | 140.3696 | 145.3558 | 0.556893 | 0.999975 | 0.999976 |
| 53 | 17.57568 | 41.36312 | 145.3524 | 17.60411 | 41.37521 | 145.3558 | 0.998385 | 0.999708 | 0.999976 |
| 54 | 10.57987 | 76.19974 | 179.2183 | 10.62702 | 76.2063 | 179.2211 | 0.995563 | 0.999914 | 0.999984 |
| 55 | 11.54874 | 92.32897 | 220.9744 | 11.59195 | 92.33439 | 220.9766 | 0.996272 | 0.999941 | 0.99999 |
| 56 | 25.36716 | 57.63172 | 236.9527 | 25.38686 | 57.6404 | 236.9548 | 0.999224 | 0.999849 | 0.999991 |
| 57 | 0.488875 | 287.1051 | 292.16 | 1.113103 | 287.1068 | 292.1617 | 0.4392 | 0.999994 | 0.999994 |
| 58 | 1.751114 | 254.0864 | 292.16 | 2.016532 | 254.0884 | 292.1617 | 0.868379 | 0.999992 | 0.999994 |
| 59 | 40.64725 | 60.73016 | 335.9387 | 40.65955 | 60.7384 | 335.9402 | 0.999698 | 0.999864 | 0.999996 |
| 60 | 5.227972 | 232.853 | 360.2298 | 5.322752 | 232.8551 | 360.2312 | 0.982193 | 0.999991 | 0.999996 |
| 61 | 7.062352 | 206.0735 | 360.2298 | 7.132799 | 206.0759 | 360.2312 | 0.990124 | 0.999988 | 0.999996 |
| 62 | 13.51987 | 188.8523 | 444.1589 | 13.5568 | 188.855 | 444.16 | 0.997276 | 0.999986 | 0.999997 |
| 63 | 26.7318 | 145.3524 | 510.7136 | 26.7505 | 145.3558 | 510.7146 | 0.999301 | 0.999976 | 0.999998 |
| 64 | 0.75624 | 528.8557 | 547.6423 | 1.253754 | 528.8567 | 547.6432 | 0.603181 | 0.999998 | 0.999998 |
| 65 | 3.998691 | 459.9368 | 629.7033 | 4.121836 | 459.9379 | 629.7041 | 0.970124 | 0.999998 | 0.999999 |
| 66 | 0.528035 | 762.9826 | 776.4161 | 1.13085 | 762.9833 | 776.4167 | 0.466937 | 0.999999 | 0.999999 |
| 67 | 2.173014 | 711.5332 | 832.5571 | 2.392068 | 711.5339 | 832.5577 | 0.908425 | 0.999999 | 0.999999 |
| 68 | 89.16149 | 100.7487 | 832.5571 | 89.1671 | 100.7536 | 832.5577 | 0.999937 | 0.999951 | 0.999999 |
| 69 | 6.24152 | 618.8083 | 957.3109 | 6.321121 | 618.8091 | 957.3114 | 0.987407 | 0.999999 | 0.999999 |
| 70 | 2.050688 | 1026.532 | 1180.352 | 2.281517 | 1026.532 | 1180.352 | 0.898826 | 1 | 1 |
| 71 | 17.27109 | 501.8773 | 1180.352 | 17.30002 | 501.8783 | 1180.352 | 0.998328 | 0.999998 | 1 |
| 72 | 66.26871 | 228.8241 | 1265.701 | 66.27625 | 228.8263 | 1265.701 | 0.999886 | 0.99999 | 1 |
| 73 | 9.357358 | 832.5571 | 1455.358 | 9.41064 | 832.5577 | 1455.359 | 0.994338 | 0.999999 | 1 |
| 74 | 47.56283 | 379.594 | 1560.592 | 47.57335 | 379.5953 | 1560.592 | 0.999779 | 0.999997 | 1 |
| 75 | 0.567839 | 2174.237 | 2212.518 | 1.149974 | 2174.237 | 2212.518 | 0.493784 | 1 | 1 |
| 76 | 130.9006 | 267.7449 | 2212.518 | 130.9045 | 267.7468 | 2212.518 | 0.999971 | 0.999993 | 1 |
| 77 | 0.845683 | 2291.113 | 2372.5 | 1.309649 | 2291.113 | 2372.5 | 0.645733 | 1 | 1 |
| 78 | 2.435895 | 2027.624 | 2372.5 | 2.63317 | 2027.624 | 2372.5 | 0.925081 | 1 | 1 |
| 79 | 4.956314 | 1992.542 | 2728.005 | 5.056189 | 1992.543 | 2728.006 | 0.980247 | 1 | 1 |
| 80 | 7.445679 | 1763.39 | 2728.005 | 7.512532 | 1763.391 | 2728.006 | 0.991101 | 1 | 1 |
| 81 | 22.05863 | 1430.178 | 3363.595 | 22.08129 | 1430.178 | 3363.595 | 0.998974 | 1 | 1 |
| 82 | 176.1174 | 360.2298 | 3363.595 | 176.1203 | 360.2312 | 3363.595 | 0.999984 | 0.999996 | 1 |
| 83 | 23.24558 | 1507.057 | 3606.809 | 23.26708 | 1507.057 | 3606.809 | 0.999076 | 1 | 1 |
| 84 | 65.122 | 1081.713 | 4447.148 | 65.12968 | 1081.714 | 4447.148 | 0.999882 | 1 | 1 |
| 85 | 2.390306 | 4768.711 | 5483.274 | 2.591054 | 4768.711 | 5483.275 | 0.922522 | 1 | 1 |
| 86 | 108.0339 | 991.3174 | 5483.274 | 108.0386 | 991.3179 | 5483.275 | 0.999957 | 0.999999 | 1 |
| 87 | 192.1774 | 762.9826 | 6304.911 | 192.18 | 762.9833 | 6304.911 | 0.999986 | 0.999999 | 1 |
| 88 | 0.608335 | 6643.83 | 6760.805 | 1.1705 | 6643.83 | 6760.805 | 0.519722 | 1 | 1 |
| 89 | 12.38682 | 3867.609 | 6760.805 | 12.42712 | 3867.61 | 6760.805 | 0.996757 | 1 | 1 |
| 90 | 8.877222 | 5388.404 | 8335.983 | 8.933369 | 5388.404 | 8335.983 | 0.993715 | 1 | 1 |
| 91 | 61.79955 | 2728.005 | 9585.081 | 61.80764 | 2728.006 | 9585.081 | 0.999869 | 1 | 1 |
| 92 | 501.8773 | 567.0962 | 9585.081 | 501.8783 | 567.0971 | 9585.081 | 0.999998 | 0.999998 | 1 |
| 93 | 28.16974 | 4370.204 | 10278.16 | 28.18749 | 4370.204 | 10278.16 | 0.999371 | 1 | 1 |
| 94 | 0.93925 | 11412.86 | 11818.28 | 1.37193 | 11412.86 | 11818.28 | 0.68462 | 1 | 1 |
| 95 | 307.8651 | 1673.435 | 15625.43 | 307.8668 | 1673.435 | 15625.43 | 0.999995 | 1 | 1 |
| 96 | 32.9639 | 7507.194 | 17966.81 | 32.97906 | 7507.194 | 17966.81 | 0.99954 | 1 | 1 |
| 97 | 0.649572 | 21769.56 | 22152.84 | 1.192453 | 21769.56 | 22152.84 | 0.544736 | 1 | 1 |
| 98 | 3.045046 | 20301.59 | 23754.67 | 3.205043 | 20301.59 | 23754.67 | 0.950079 | 1 | 1 |
| 99 | 176.1174 | 4938.11 | 27314.16 | 176.1203 | 4938.11 | 27314.16 | 0.999984 | 1 | 1 |
| 100 | 2.7766 | 25472.32 | 29289.19 | 2.951187 | 25472.32 | 29289.19 | 0.940842 | 1 | 1 |
| 101 | 892.7575 | 1533.591 | 29289.19 | 892.7581 | 1533.591 | 29289.19 | 0.999999 | 1 | 1 |
| 102 | 16.38853 | 20659.03 | 36113.19 | 16.41901 | 20659.03 | 36113.19 | 0.998144 | 1 | 1 |
| 103 | 122.0735 | 10830.66 | 44527.08 | 122.0776 | 10830.66 | 44527.08 | 0.999966 | 1 | 1 |
| 104 | 93.95465 | 14571.78 | 51199.2 | 93.95998 | 14571.78 | 51199.2 | 0.999943 | 1 | 1 |
| 105 | 414.2083 | 7639.369 | 63127.94 | 414.2095 | 7639.369 | 63127.94 | 0.999997 | 1 | 1 |
| 106 | 1.037396 | 65370.43 | 67692.58 | 1.4409 | 65370.43 | 67692.58 | 0.719964 | 1 | 1 |
| 107 | 0.6916 | 76489.21 | 77835.91 | 1.215858 | 76489.21 | 77835.91 | 0.568817 | 1 | 1 |
| 108 | 7.577938 | 56851.56 | 77835.91 | 7.643634 | 56851.56 | 77835.91 | 0.991405 | 1 | 1 |
| 109 | 3.398002 | 71331.39 | 83464.06 | 3.542093 | 71331.39 | 83464.06 | 0.959321 | 1 | 1 |
| 110 | 538.167 | 8938.74 | 83464.06 | 538.168 | 8938.74 | 83464.06 | 0.999998 | 1 | 1 |
| 111 | 12.60561 | 62035.71 | 95970.65 | 12.64522 | 62035.71 | 95970.65 | 0.996868 | 1 | 1 |
| 112 | 1405.433 | 5025.053 | 95970.65 | 1405.433 | 5025.053 | 95970.65 | 1 | 1 | 1 |
| 113 | 46.73973 | 42999.61 | 102910.1 | 46.75042 | 42999.61 | 102910.1 | 0.999771 | 1 | 1 |
| 114 | 45.93085 | 50313.36 | 118330.5 | 45.94174 | 50313.36 | 118330.5 | 0.999763 | 1 | 1 |
| 115 | 167.1331 | 38054.45 | 156449.7 | 167.1361 | 38054.45 | 156449.7 | 0.999982 | 1 | 1 |
| 116 | 287.1051 | 28284.44 | 156449.7 | 287.1068 | 28284.44 | 156449.7 | 0.999994 | 1 | 1 |
| 117 | 3.217113 | 156449.7 | 179892.8 | 3.368949 | 156449.7 | 179892.8 | 0.954931 | 1 | 1 |
| 118 | 21.67658 | 126886.8 | 221805.4 | 21.69963 | 126886.8 | 221805.4 | 0.998938 | 1 | 1 |
| 119 | 608.1017 | 26841.58 | 221805.4 | 608.1026 | 26841.58 | 221805.4 | 0.999999 | 1 | 1 |
| 120 | 1763.39 | 16180.5 | 273483.1 | 1763.391 | 16180.5 | 273483.1 | 1 | 1 | 1 |
| 121 | 0.734472 | 288184.2 | 293258.1 | 1.240745 | 288184.2 | 293258.1 | 0.59196 | 1 | 1 |
| 122 | 2212.518 | 17655.95 | 337201.1 | 2212.518 | 17655.95 | 337201.1 | 1 | 1 | 1 |
| 123 | 15.01633 | 233728.5 | 361583.4 | 15.04959 | 233728.5 | 361583.4 | 0.99779 | 1 | 1 |
| 124 | 1.140601 | 430533.7 | 445827.5 | 1.516895 | 430533.7 | 445827.5 | 0.751931 | 1 | 1 |
| 125 | 58.64656 | 189562.9 | 445827.5 | 58.65509 | 189562.9 | 445827.5 | 0.999855 | 1 | 1 |
| 126 | 9.357358 | 374427.9 | 512632.1 | 9.41064 | 374427.9 | 512632.1 | 0.994338 | 1 | 1 |
| 127 | 940.7476 | 54901.3 | 512632.1 | 940.7482 | 54901.3 | 512632.1 | 0.999999 | 1 | 1 |
| 128 | 8050.022 | 10830.66 | 549699.5 | 8050.023 | 10830.66 | 549699.5 | 1 | 1 | 1 |
| 129 | 66.26871 | 283198.1 | 677772.2 | 66.27625 | 283198.1 | 677772.2 | 0.999886 | 1 | 1 |
| 130 | 892.7575 | 101129.5 | 835684.1 | 892.7581 | 101129.5 | 835684.1 | 0.999999 | 1 | 1 |
| 131 | 6195.824 | 29289.19 | 960906.4 | 6195.824 | 29289.19 | 960906.4 | 1 | 1 | 1 |
| 132 | 0.778238 | 1164286 | 1184785 | 1.267144 | 1164286 | 1184785 | 0.614167 | 1 | 1 |
| 133 | 3.720448 | 1104893 | 1270454 | 3.852497 | 1104893 | 1270454 | 0.965724 | 1 | 1 |
| 134 | 4.220091 | 1085776 | 1270454 | 4.336954 | 1085776 | 1270454 | 0.973054 | 1 | 1 |
| 135 | 17.88562 | 944281 | 1460824 | 17.91356 | 944281 | 1460824 | 0.998441 | 1 | 1 |
| 136 | 28.66602 | 896110.6 | 1566453 | 28.68346 | 896110.6 | 1566453 | 0.999392 | 1 | 1 |
| 137 | 74.88122 | 765848.4 | 1801177 | 74.8879 | 765848.4 | 1801177 | 0.999911 | 1 | 1 |
| 138 | 3305.398 | 106565.8 | 1801177 | 3305.398 | 106565.8 | 1801177 | 1 | 1 | 1 |
| 139 | 217.1511 | 632068.7 | 2220827 | 217.1534 | 632068.7 | 2220827 | 0.999989 | 1 | 1 |
| 140 | 313.2856 | 579248.5 | 2381411 | 313.2872 | 579248.5 | 2381411 | 0.999995 | 1 | 1 |
| 141 | 1.249367 | 3260408 | 3376227 | 1.600287 | 3260408 | 3376227 | 0.780714 | 1 | 1 |
| 142 | 1310.662 | 408571 | 3376227 | 1310.662 | 408571 | 3376227 | 1 | 1 | 1 |
| 143 | 1644.482 | 387728.7 | 3620355 | 1644.482 | 387728.7 | 3620355 | 1 | 1 | 1 |
| 144 | 23343.67 | 53017.95 | 3620355 | 23343.67 | 53017.95 | 3620355 | 1 | 1 | 1 |
| 145 | 0.822953 | 5043925 | 5132731 | 1.295088 | 5043925 | 5132731 | 0.635442 | 1 | 1 |
| 146 | 5483.274 | 268751.4 | 5132731 | 5483.275 | 268751.4 | 5132731 | 1 | 1 | 1 |
| 147 | 4.698247 | 4703803 | 5503867 | 4.803491 | 4703803 | 5503867 | 0.97809 | 1 | 1 |
| 148 | 21.30113 | 4090818 | 6328589 | 21.32459 | 4090818 | 6328589 | 0.9989 | 1 | 1 |
| 149 | 12453.57 | 206848.6 | 6786196 | 12453.57 | 206848.6 | 6786196 | 1 | 1 | 1 |
| 150 | 95.60896 | 3317812 | 7803067 | 95.61419 | 3317812 | 7803067 | 0.999945 | 1 | 1 |
| 151 | 762.9826 | 1410712 | 7803067 | 762.9833 | 1410712 | 7803067 | 0.999999 | 1 | 1 |
| 152 | 22939.78 | 164859.6 | 8367290 | 22939.78 | 164859.6 | 8367290 | 1 | 1 | 1 |
| 153 | 4.296432 | 8972310 | 10316758 | 4.411273 | 8972310 | 10316758 | 0.973967 | 1 | 1 |
| 154 | 428.9222 | 2509423 | 10316758 | 428.9234 | 2509423 | 10316758 | 0.999997 | 1 | 1 |
| 155 | 37.90547 | 7276891 | 12720426 | 37.91866 | 7276891 | 12720426 | 0.999652 | 1 | 1 |
| 156 | 1924.189 | 1770013 | 14626506 | 1924.19 | 1770013 | 14626506 | 1 | 1 | 1 |
| 157 | 94310.18 | 95970.65 | 14626506 | 94310.18 | 95970.65 | 14626506 | 1 | 1 | 1 |
| 158 | 330.1263 | 5132731 | 18034289 | 330.1278 | 5132731 | 18034289 | 0.999995 | 1 | 1 |
| 159 | 8632.102 | 1164286 | 22236040 | 8632.102 | 1164286 | 22236040 | 1 | 1 | 1 |
| 160 | 0.868671 | 23431336 | 23843880 | 1.324609 | 23431336 | 23843880 | 0.655794 | 1 | 1 |
| 161 | 50313.36 | 401502 | 27416744 | 50313.36 | 401502 | 27416744 | 1 | 1 | 1 |
| 162 | 1.364225 | 28390668 | 29399189 | 1.691481 | 28390668 | 29399189 | 0.806527 | 1 | 1 |
| 163 | 2874.649 | 3148561 | 29399189 | 2874.649 | 3148561 | 29399189 | 1 | 1 | 1 |
| 164 | 14.24856 | 24690886 | 33804483 | 14.28361 | 24690886 | 33804483 | 0.997546 | 1 | 1 |
| 165 | 122.0735 | 15412753 | 36248812 | 122.0776 | 15412753 | 36248812 | 0.999966 | 1 | 1 |
| 166 | 38724.45 | 714205.7 | 36248812 | 38724.45 | 714205.7 | 36248812 | 1 | 1 | 1 |
| 167 | 133.2054 | 18674921 | 44694306 | 133.2092 | 18674921 | 44694306 | 0.999972 | 1 | 1 |
| 168 | 25031.6 | 1679720 | 55107489 | 25031.6 | 1679720 | 55107489 | 1 | 1 | 1 |
| 169 | 173721.7 | 408571 | 63365019 | 173721.7 | 408571 | 63365019 | 1 | 1 | 1 |
| 170 | 1243.802 | 12284060 | 67946806 | 1243.802 | 12284060 | 67946806 | 1 | 1 | 1 |
| 171 | 2824.912 | 8222520 | 67946806 | 2824.912 | 8222520 | 67946806 | 1 | 1 | 1 |
| 172 | 4.956314 | 83777514 | 96331078 | 5.056189 | 83777514 | 96331078 | 0.980247 | 1 | 1 |
| 173 | 13589.18 | 5408640 | 1.03E+08 | 13589.18 | 5408640 | 1.03E+08 | 1 | 1 | 1 |
| 174 | 0.915447 | 1.17E+08 | 1.19E+08 | 1.355745 | 1.17E+08 | 1.19E+08 | 0.675236 | 1 | 1 |
| 175 | 50.12012 | 67946806 | 1.19E+08 | 50.1301 | 67946806 | 1.19E+08 | 0.999801 | 1 | 1 |
| 176 | 11613.8 | 7027262 | 1.19E+08 | 11613.8 | 7027262 | 1.19E+08 | 1 | 1 | 1 |
| 177 | 217967.8 | 779332.3 | 1.19E+08 | 217967.8 | 779332.3 | 1.19E+08 | 1 | 1 | 1 |
| 178 | 5.81508 | 1.09E+08 | 1.27E+08 | 5.900437 | 1.09E+08 | 1.27E+08 | 0.985534 | 1 | 1 |
| 179 | 30.20788 | 94664370 | 1.46E+08 | 30.22442 | 94664370 | 1.46E+08 | 0.999453 | 1 | 1 |
| 180 | 155.8627 | 76776468 | 1.81E+08 | 155.866 | 76776468 | 1.81E+08 | 0.999979 | 1 | 1 |
| 181 | 803.9967 | 58069786 | 2.39E+08 | 803.9973 | 58069786 | 2.39E+08 | 0.999999 | 1 | 1 |
| 182 | 108442 | 3496161 | 2.39E+08 | 108442 | 3496161 | 2.39E+08 | 1 | 1 | 1 |
| 183 | 5025.053 | 29399189 | 2.75E+08 | 5025.053 | 29399189 | 2.75E+08 | 1 | 1 | 1 |
| 184 | 1.485734 | 2.84E+08 | 2.94E+08 | 1.790923 | 2.84E+08 | 2.94E+08 | 0.829591 | 1 | 1 |
| 185 | 314463 | 1897999 | 2.94E+08 | 314463 | 1897999 | 2.94E+08 | 1 | 1 | 1 |
| 186 | 17.57568 | 2.47E+08 | 3.38E+08 | 17.60411 | 2.47E+08 | 3.38E+08 | 0.998385 | 1 | 1 |
| 187 | 4147.268 | 40959331 | 3.38E+08 | 4147.268 | 40959331 | 3.38E+08 | 1 | 1 | 1 |
| 188 | 188.8523 | 1.87E+08 | 4.48E+08 | 188.855 | 1.87E+08 | 4.48E+08 | 0.999986 | 1 | 1 |
| 189 | 21392.9 | 26942383 | 5.15E+08 | 21392.9 | 26942383 | 5.15E+08 | 1 | 1 | 1 |
| 190 | 50313.36 | 15684117 | 5.15E+08 | 50313.36 | 15684117 | 5.15E+08 | 1 | 1 | 1 |
| 191 | 1012560 | 1512717 | 5.52E+08 | 1012560 | 1512717 | 5.52E+08 | 1 | 1 | 1 |
| 192 | 0.963339 | 6.23E+08 | 6.34E+08 | 1.388532 | 6.23E+08 | 6.34E+08 | 0.693782 | 1 | 1 |
| 193 | 6.466016 | 5.81E+08 | 6.8E+08 | 6.542887 | 5.81E+08 | 6.8E+08 | 0.988251 | 1 | 1 |
| 194 | 2027.624 | 1.23E+08 | 6.8E+08 | 2027.624 | 1.23E+08 | 6.8E+08 | 1 | 1 | 1 |
| 195 | 35.97112 | 5.06E+08 | 7.82E+08 | 35.98502 | 5.06E+08 | 7.82E+08 | 0.999614 | 1 | 1 |
| 196 | 110351.3 | 16527215 | 8.39E+08 | 110351.3 | 16527215 | 8.39E+08 | 1 | 1 | 1 |
| 197 | 5.712979 | 8.99E+08 | 1.03E+09 | 5.799838 | 8.99E+08 | 1.03E+09 | 0.985024 | 1 | 1 |
| 198 | 21769.56 | 70360478 | 1.19E+09 | 21769.56 | 70360478 | 1.19E+09 | 1 | 1 | 1 |
| 199 | 66.26871 | 7.3E+08 | 1.28E+09 | 66.27625 | 7.3E+08 | 1.28E+09 | 0.999886 | 1 | 1 |
| 200 | 1100.758 | 3.1E+08 | 1.28E+09 | 1100.759 | 3.1E+08 | 1.28E+09 | 1 | 1 | 1 |
| 201 | 569226.4 | 9454616 | 1.47E+09 | 569226.4 | 9454616 | 1.47E+09 | 1 | 1 | 1 |
| 202 | 762.9826 | 5.15E+08 | 1.81E+09 | 762.9833 | 5.15E+08 | 1.81E+09 | 0.999999 | 1 | 1 |
| 203 | 6088.625 | 2.19E+08 | 1.81E+09 | 6088.625 | 2.19E+08 | 1.81E+09 | 1 | 1 | 1 |
| 204 | 233728.5 | 35005319 | 2.39E+09 | 233728.5 | 35005319 | 2.39E+09 | 1 | 1 | 1 |
| 205 | 33678 | 1.44E+08 | 2.75E+09 | 33678 | 1.44E+08 | 2.75E+09 | 1 | 1 | 1 |
| 206 | 2936248 | 5043925 | 2.75E+09 | 2936248 | 5043925 | 2.75E+09 | 1 | 1 | 1 |
| 207 | 8784.083 | 3.16E+08 | 2.95E+09 | 8784.083 | 3.16E+08 | 2.95E+09 | 1 | 1 | 1 |
| 208 | 1.614488 | 3.27E+09 | 3.39E+09 | 1.899098 | 3.27E+09 | 3.39E+09 | 0.850134 | 1 | 1 |
| 209 | 1.012405 | 3.57E+09 | 3.63E+09 | 1.423012 | 3.57E+09 | 3.63E+09 | 0.711452 | 1 | 1 |
| 210 | 42.83289 | 2.9E+09 | 4.48E+09 | 42.84456 | 2.9E+09 | 4.48E+09 | 0.999728 | 1 | 1 |
| 211 | 186283.1 | 88280966 | 4.48E+09 | 186283.1 | 88280966 | 4.48E+09 | 1 | 1 | 1 |
| 212 | 267.7449 | 2.15E+09 | 5.15E+09 | 267.7468 | 2.15E+09 | 5.15E+09 | 0.999993 | 1 | 1 |
| 213 | 254.0864 | 2.35E+09 | 5.52E+09 | 254.0884 | 2.35E+09 | 5.52E+09 | 0.999992 | 1 | 1 |
| 214 | 2509423 | 15146083 | 5.52E+09 | 2509423 | 15146083 | 5.52E+09 | 1 | 1 | 1 |
| 215 | 3305.398 | 1.42E+09 | 7.83E+09 | 3305.398 | 1.42E+09 | 7.83E+09 | 1 | 1 | 1 |
| 216 | 1030387 | 50502314 | 7.83E+09 | 1030387 | 50502314 | 7.83E+09 | 1 | 1 | 1 |
| 217 | 8938.74 | 1.25E+09 | 1.04E+10 | 8938.74 | 1.25E+09 | 1.04E+10 | 1 | 1 | 1 |
| 218 | 1164286 | 78128232 | 1.19E+10 | 1164286 | 78128232 | 1.19E+10 | 1 | 1 | 1 |
| 219 | 6.581202 | 1.11E+10 | 1.28E+10 | 6.656742 | 1.11E+10 | 1.28E+10 | 0.988652 | 1 | 1 |
| 220 | 5699381 | 26942383 | 1.47E+10 | 5699381 | 26942383 | 1.47E+10 | 1 | 1 | 1 |
| 221 | 87.61873 | 9.01E+09 | 1.57E+10 | 87.62444 | 9.01E+09 | 1.57E+10 | 0.999935 | 1 | 1 |
| 222 | 53017.95 | 8.24E+08 | 1.57E+10 | 53017.95 | 8.24E+08 | 1.57E+10 | 1 | 1 | 1 |
| 223 | 1.062704 | 2.19E+10 | 2.23E+10 | 1.459226 | 2.19E+10 | 2.23E+10 | 0.728266 | 1 | 1 |
| 224 | 1159.93 | 6.35E+09 | 2.23E+10 | 1159.93 | 6.35E+09 | 2.23E+10 | 1 | 1 | 1 |
| 225 | 7.988731 | 2.05E+10 | 2.39E+10 | 8.051075 | 2.05E+10 | 2.39E+10 | 0.992256 | 1 | 1 |
| 226 | 51.00273 | 1.78E+10 | 2.75E+10 | 51.01254 | 1.78E+10 | 2.75E+10 | 0.999808 | 1 | 1 |
| 227 | 503762.6 | 4.03E+08 | 2.75E+10 | 503762.6 | 4.03E+08 | 2.75E+10 | 1 | 1 | 1 |
| 228 | 324.4145 | 1.44E+10 | 3.39E+10 | 324.416 | 1.44E+10 | 3.39E+10 | 0.999995 | 1 | 1 |
| 229 | 15355.09 | 3.9E+09 | 3.64E+10 | 15355.09 | 3.9E+09 | 3.64E+10 | 1 | 1 | 1 |
| 230 | 1.751114 | 4.33E+10 | 4.49E+10 | 2.016532 | 4.33E+10 | 4.49E+10 | 0.868379 | 1 | 1 |
| 231 | 2063.323 | 1.09E+10 | 4.49E+10 | 2063.324 | 1.09E+10 | 4.49E+10 | 1 | 1 | 1 |
| 232 | 26.7318 | 3.77E+10 | 5.16E+10 | 26.7505 | 3.77E+10 | 5.16E+10 | 0.999301 | 1 | 1 |
| 233 | 13123.01 | 7.7E+09 | 6.36E+10 | 13123.01 | 7.7E+09 | 6.36E+10 | 1 | 1 | 1 |
| 234 | 6219093 | 1.74E+08 | 6.36E+10 | 6219093 | 1.74E+08 | 6.36E+10 | 1 | 1 | 1 |
| 235 | 379.594 | 2.85E+10 | 6.82E+10 | 379.5953 | 2.85E+10 | 6.82E+10 | 0.999997 | 1 | 1 |
| 236 | 203269.8 | 2.08E+09 | 6.82E+10 | 203269.8 | 2.08E+09 | 6.82E+10 | 1 | 1 | 1 |
| 237 | 11062740 | 1.54E+08 | 8.41E+10 | 11062740 | 1.54E+08 | 8.41E+10 | 1 | 1 | 1 |
| 238 | 83464.06 | 5.06E+09 | 9.67E+10 | 83464.06 | 5.06E+09 | 9.67E+10 | 1 | 1 | 1 |
| 239 | 5388.404 | 1.87E+10 | 1.04E+11 | 5388.404 | 1.87E+10 | 1.04E+11 | 1 | 1 | 1 |
| 240 | 1.114299 | 1.44E+11 | 1.47E+11 | 1.497218 | 1.44E+11 | 1.47E+11 | 0.744246 | 1 | 1 |
| 241 | 2690874 | 9.65E+08 | 1.47E+11 | 2690874 | 9.65E+08 | 1.47E+11 | 1 | 1 | 1 |
| 242 | 8.877222 | 1.35E+11 | 1.58E+11 | 8.933369 | 1.35E+11 | 1.58E+11 | 0.993715 | 1 | 1 |
| 243 | 530842.4 | 3.11E+09 | 1.58E+11 | 530842.4 | 3.11E+09 | 1.58E+11 | 1 | 1 | 1 |
| 244 | 7.577938 | 1.58E+11 | 1.81E+11 | 7.643634 | 1.58E+11 | 1.81E+11 | 0.991405 | 1 | 1 |
| 245 | 76489.21 | 1.07E+10 | 1.81E+11 | 76489.21 | 1.07E+10 | 1.81E+11 | 1 | 1 | 1 |
| 246 | 115.846 | 1.28E+11 | 2.23E+11 | 115.8503 | 1.28E+11 | 2.23E+11 | 0.999963 | 1 | 1 |
| 247 | 414.2083 | 9.5E+10 | 2.23E+11 | 414.2095 | 9.5E+10 | 2.23E+11 | 0.999997 | 1 | 1 |
| 248 | 3376227 | 1.78E+09 | 2.76E+11 | 3376227 | 1.78E+09 | 2.76E+11 | 1 | 1 | 1 |
| 249 | 2824.912 | 7.19E+10 | 2.95E+11 | 2824.912 | 7.19E+10 | 2.95E+11 | 1 | 1 | 1 |
| 250 | 1085776 | 5.34E+09 | 3.64E+11 | 1085776 | 5.34E+09 | 3.64E+11 | 1 | 1 | 1 |
| 251 | 35621640 | 3.89E+08 | 3.64E+11 | 35621640 | 3.89E+08 | 3.64E+11 | 1 | 1 | 1 |
| 252 | 26841.58 | 5.53E+10 | 5.16E+11 | 26841.58 | 5.53E+10 | 5.16E+11 | 1 | 1 | 1 |
| 253 | 21473247 | 9.48E+08 | 5.16E+11 | 21473247 | 9.48E+08 | 5.16E+11 | 1 | 1 | 1 |
| 254 | 131394.2 | 3.33E+10 | 6.37E+11 | 131394.2 | 3.33E+10 | 6.37E+11 | 1 | 1 | 1 |
| 255 | 1.896278 | 6.59E+11 | 6.83E+11 | 2.143798 | 6.59E+11 | 6.83E+11 | 0.884541 | 1 | 1 |
| 256 | 32.9639 | 5.74E+11 | 7.85E+11 | 32.97906 | 5.74E+11 | 7.85E+11 | 0.99954 | 1 | 1 |
| 257 | 15412753 | 2.31E+09 | 8.42E+11 | 15412753 | 2.31E+09 | 8.42E+11 | 1 | 1 | 1 |
| 258 | 408571 | 2.95E+10 | 9.68E+11 | 408571 | 2.95E+10 | 9.68E+11 | 1 | 1 | 1 |
| 259 | 1.167251 | 1.02E+12 | 1.04E+12 | 1.537034 | 1.02E+12 | 1.04E+12 | 0.759418 | 1 | 1 |
| 260 | 1.37E+08 | 4.72E+08 | 1.04E+12 | 1.37E+08 | 4.72E+08 | 1.04E+12 | 1 | 1 | 1 |
| 261 | 72.31224 | 8.27E+11 | 1.28E+12 | 72.31915 | 8.27E+11 | 1.28E+12 | 0.999904 | 1 | 1 |
| 262 | 528.8557 | 6.71E+11 | 1.58E+12 | 528.8567 | 6.71E+11 | 1.58E+12 | 0.999998 | 1 | 1 |
| 263 | 8784.083 | 2.85E+11 | 1.58E+12 | 8784.083 | 2.85E+11 | 1.58E+12 | 1 | 1 | 1 |
| 264 | 6111491 | 1.17E+10 | 1.81E+12 | 6111491 | 1.17E+10 | 1.81E+12 | 1 | 1 | 1 |
| 265 | 2.19E+08 | 8.69E+08 | 2.24E+12 | 2.19E+08 | 8.69E+08 | 2.24E+12 | 1 | 1 | 1 |
| 266 | 143375.7 | 1.63E+11 | 2.76E+12 | 143375.7 | 1.63E+11 | 2.76E+12 | 1 | 1 | 1 |
| 267 | 8.72265 | 2.57E+12 | 2.96E+12 | 8.779785 | 2.57E+12 | 2.96E+12 | 0.993492 | 1 | 1 |
| 268 | 28284.44 | 3.58E+11 | 2.96E+12 | 28284.44 | 3.58E+11 | 2.96E+12 | 1 | 1 | 1 |
| 269 | 41680481 | 6.24E+09 | 3.4E+12 | 41680481 | 6.24E+09 | 3.4E+12 | 1 | 1 | 1 |
| 270 | 153.166 | 2.09E+12 | 3.65E+12 | 153.1692 | 2.09E+12 | 3.65E+12 | 0.999979 | 1 | 1 |
| 271 | 206848.6 | 2.35E+11 | 4.5E+12 | 206848.6 | 2.35E+11 | 4.5E+12 | 1 | 1 | 1 |
| 272 | 2680.806 | 1.47E+12 | 5.17E+12 | 2680.806 | 1.47E+12 | 5.17E+12 | 1 | 1 | 1 |
| 273 | 94664370 | 5.52E+09 | 5.17E+12 | 94664370 | 5.52E+09 | 5.17E+12 | 1 | 1 | 1 |
| 274 | 2340208 | 8.12E+10 | 5.55E+12 | 2340208 | 8.12E+10 | 5.55E+12 | 1 | 1 | 1 |
| 275 | 2.84E+08 | 3.11E+09 | 6.84E+12 | 2.84E+08 | 3.11E+09 | 6.84E+12 | 1 | 1 | 1 |
| 276 | 1.221626 | 7.73E+12 | 7.86E+12 | 1.578724 | 7.73E+12 | 7.86E+12 | 0.773806 | 1 | 1 |
| 277 | 10.95734 | 7.2E+12 | 8.43E+12 | 11.00288 | 7.2E+12 | 8.43E+12 | 0.995861 | 1 | 1 |
| 278 | 46920.63 | 9.03E+11 | 8.43E+12 | 46920.63 | 9.03E+11 | 8.43E+12 | 1 | 1 | 1 |
| 279 | 86.10267 | 6.27E+12 | 9.69E+12 | 86.10847 | 6.27E+12 | 9.69E+12 | 0.999933 | 1 | 1 |
| 280 | 2.050688 | 1.15E+13 | 1.2E+13 | 2.281517 | 1.15E+13 | 1.2E+13 | 0.898826 | 1 | 1 |
| 281 | 675.2359 | 5.08E+12 | 1.2E+13 | 675.2366 | 5.08E+12 | 1.2E+13 | 0.999999 | 1 | 1 |
| 282 | 11062740 | 8.26E+10 | 1.28E+13 | 11062740 | 8.26E+10 | 1.28E+13 | 1 | 1 | 1 |
| 283 | 38197363 | 3.51E+10 | 1.28E+13 | 38197363 | 3.51E+10 | 1.28E+13 | 1 | 1 | 1 |
| 284 | 1.44E+09 | 1.94E+09 | 1.47E+13 | 1.44E+09 | 1.94E+09 | 1.47E+13 | 1 | 1 | 1 |
| 285 | 5295.174 | 3.84E+12 | 1.58E+13 | 5295.174 | 3.84E+12 | 1.58E+13 | 1 | 1 | 1 |
| 286 | 821225.2 | 4.82E+11 | 1.58E+13 | 821225.2 | 4.82E+11 | 1.58E+13 | 1 | 1 | 1 |
| 287 | 762.9826 | 7.59E+12 | 1.82E+13 | 762.9833 | 7.59E+12 | 1.82E+13 | 0.999999 | 1 | 1 |
| 288 | 41524.53 | 2.71E+12 | 2.24E+13 | 41524.53 | 2.71E+12 | 2.24E+13 | 1 | 1 | 1 |
| 289 | 325633.6 | 1.78E+12 | 3.41E+13 | 325633.6 | 1.78E+12 | 3.41E+13 | 1 | 1 | 1 |
| 290 | 14373441 | 2.23E+11 | 3.41E+13 | 14373441 | 2.23E+11 | 3.41E+13 | 1 | 1 | 1 |
| 291 | 10.03769 | 4.83E+13 | 5.55E+13 | 10.08738 | 4.83E+13 | 5.55E+13 | 0.995074 | 1 | 1 |
| 292 | 2553605 | 1.09E+12 | 5.55E+13 | 2553605 | 1.09E+12 | 5.55E+13 | 1 | 1 | 1 |
| 293 | 1.277489 | 6.27E+13 | 6.38E+13 | 1.622337 | 6.27E+13 | 6.38E+13 | 0.787437 | 1 | 1 |
| 294 | 12.1718 | 5.85E+13 | 6.85E+13 | 12.21281 | 5.85E+13 | 6.85E+13 | 0.996642 | 1 | 1 |
| 295 | 202.508 | 3.92E+13 | 6.85E+13 | 202.5105 | 3.92E+13 | 6.85E+13 | 0.999988 | 1 | 1 |
| 296 | 102.5226 | 5.09E+13 | 7.87E+13 | 102.5275 | 5.09E+13 | 7.87E+13 | 0.999952 | 1 | 1 |
| 297 | 2.52E+08 | 9.02E+10 | 8.44E+13 | 2.52E+08 | 9.02E+10 | 8.44E+13 | 1 | 1 | 1 |
| 298 | 862.132 | 4.13E+13 | 9.71E+13 | 862.1326 | 4.13E+13 | 9.71E+13 | 0.999999 | 1 | 1 |
| 299 | 4075.512 | 2.76E+13 | 9.71E+13 | 4075.512 | 2.76E+13 | 9.71E+13 | 1 | 1 | 1 |
| 300 | 5043925 | 1.42E+12 | 9.71E+13 | 5043925 | 1.42E+12 | 9.71E+13 | 1 | 1 | 1 |
| 301 | 20025264 | 6.26E+11 | 9.71E+13 | 20025264 | 6.26E+11 | 9.71E+13 | 1 | 1 | 1 |
| 302 | 4.33E+09 | 1.02E+10 | 1.04E+14 | 4.33E+09 | 1.02E+10 | 1.04E+14 | 1 | 1 | 1 |
| 303 | 7249.664 | 3.12E+13 | 1.28E+14 | 7249.664 | 3.12E+13 | 1.28E+14 | 1 | 1 | 1 |
| 304 | 82019.97 | 1.69E+13 | 1.58E+14 | 82019.97 | 1.69E+13 | 1.58E+14 | 1 | 1 | 1 |
| 305 | 60962.37 | 2.2E+13 | 1.82E+14 | 60962.37 | 2.2E+13 | 1.82E+14 | 1 | 1 | 1 |
| 306 | 1.57E+08 | 3.34E+11 | 1.82E+14 | 1.57E+08 | 3.34E+11 | 1.82E+14 | 1 | 1 | 1 |
| 307 | 94664370 | 6.15E+11 | 2.24E+14 | 94664370 | 6.15E+11 | 2.24E+14 | 1 | 1 | 1 |
| 308 | 2.215096 | 2.32E+14 | 2.41E+14 | 2.430361 | 2.32E+14 | 2.41E+14 | 0.911427 | 1 | 1 |
| 309 | 4.4E+09 | 3.16E+10 | 2.41E+14 | 4.4E+09 | 3.16E+10 | 2.41E+14 | 1 | 1 | 1 |
| 310 | 50.12012 | 2.02E+14 | 2.77E+14 | 50.1301 | 2.02E+14 | 2.77E+14 | 0.999801 | 1 | 1 |
| 311 | 512632.1 | 1.45E+13 | 2.77E+14 | 512632.1 | 1.45E+13 | 2.77E+14 | 1 | 1 | 1 |
| 312 | 1650658 | 9.04E+12 | 2.97E+14 | 1650658 | 9.04E+12 | 2.97E+14 | 1 | 1 | 1 |
| 313 | 1081.713 | 1.53E+14 | 3.66E+14 | 1081.714 | 1.53E+14 | 3.66E+14 | 1 | 1 | 1 |
| 314 | 1.23E+09 | 1.66E+11 | 3.66E+14 | 1.23E+09 | 1.66E+11 | 3.66E+14 | 1 | 1 | 1 |
| 315 | 4310720 | 8.88E+12 | 4.51E+14 | 4310720 | 8.88E+12 | 4.51E+14 | 1 | 1 | 1 |
| 316 | 1.334909 | 5.46E+14 | 5.56E+14 | 1.667928 | 5.46E+14 | 5.56E+14 | 0.80034 | 1 | 1 |
| 317 | 23343.67 | 1.01E+14 | 5.56E+14 | 23343.67 | 1.01E+14 | 5.56E+14 | 1 | 1 | 1 |
| 318 | 33219603 | 4.19E+12 | 6.39E+14 | 33219603 | 4.19E+12 | 6.39E+14 | 1 | 1 | 1 |
| 319 | 122.0735 | 4.43E+14 | 6.85E+14 | 122.0776 | 4.43E+14 | 6.85E+14 | 0.999966 | 1 | 1 |
| 320 | 36248812 | 5.08E+12 | 7.88E+14 | 36248812 | 5.08E+12 | 7.88E+14 | 1 | 1 | 1 |
| 321 | 9.66E+09 | 7.71E+10 | 7.88E+14 | 9.66E+09 | 7.71E+10 | 7.88E+14 | 1 | 1 | 1 |
| 322 | 503762.6 | 5.75E+13 | 9.72E+14 | 503762.6 | 5.75E+13 | 9.72E+14 | 1 | 1 | 1 |
| 323 | 11.54874 | 1.04E+15 | 1.2E+15 | 11.59195 | 1.04E+15 | 1.2E+15 | 0.996272 | 1 | 1 |
| 324 | 267.7449 | 8.45E+14 | 1.48E+15 | 267.7468 | 8.45E+14 | 1.48E+15 | 0.999993 | 1 | 1 |
| 325 | 3.05E+08 | 2.71E+12 | 1.48E+15 | 3.05E+08 | 2.71E+12 | 1.48E+15 | 1 | 1 | 1 |
| 326 | 89499.16 | 1.92E+14 | 1.58E+15 | 89499.16 | 1.92E+14 | 1.58E+15 | 1 | 1 | 1 |
| 327 | 6.69E+08 | 1.69E+12 | 1.58E+15 | 6.69E+08 | 1.69E+12 | 1.58E+15 | 1 | 1 | 1 |
| 328 | 3.33E+10 | 7.57E+10 | 1.82E+15 | 3.33E+10 | 7.57E+10 | 1.82E+15 | 1 | 1 | 1 |
| 329 | 807016.5 | 1.26E+14 | 2.41E+15 | 807016.5 | 1.26E+14 | 2.41E+15 | 1 | 1 | 1 |
| 330 | 2.56E+09 | 1.35E+12 | 2.97E+15 | 2.56E+09 | 1.35E+12 | 2.97E+15 | 1 | 1 | 1 |
| 331 | 143375.7 | 3.66E+14 | 3.41E+15 | 143375.7 | 3.66E+14 | 3.41E+15 | 1 | 1 | 1 |
